# Optimized spirooxindole-pyrazole hybrids targeting the p53-MDM2 interplay induce apoptosis and synergize with doxorubicin in A549 cells

**DOI:** 10.1038/s41598-023-31209-3

**Published:** 2023-05-08

**Authors:** Mohammad Shahidul Islam, Abdullah Mohammed Al-Majid, Essam Nageh Sholkamy, Assem Barakat, Maurizio Viale, Paola Menichini, Andrea Speciale, Fabrizio Loiacono, Mohammad Azam, Ved Prakash Verma, Sammer Yousuf, Mohamed Teleb

**Affiliations:** 1 Department of Chemistry, College of Science, King Saud University, P.O. Box 2455, Riyadh, 11451 Saudi Arabia; 2 Department of Botany and Microbiology, College of Science, King Saud University, P.O. Box 2455, Riyadh, 11451 Saudi Arabia; 3 U.O.C. Bioterapie, IRCCS Ospedale Policlinico San Martino, Largo R. Benzi 10, 16132 Genova, Italy; 4 U.O.C. Mutagenesi e Prevenzione Oncologica, IRCCS Ospedale Policlinico San Martino, Largo R. Benzi 10, 16132 Genova, Italy; 5 U.O.C. Immunologia, IRCCS Ospedale Policlinico San Martino, Largo R. Benzi 10, 16132 Genova, Italy; 6 Department of Chemistry, Banasthali Vidyapith, Banasthali, 304022 Rajasthan India; 7 H.E.J. Research Institute of Chemistry, International Centre for Chemical and Biological Sciences, University of Karachi, Karachi, 75270 Pakistan; 8 Department of Pharmaceutical Chemistry, Faculty of Pharmacy, Alexandria University, Alexandria, 21521 Egypt

**Keywords:** Cancer, Drug discovery

## Abstract

Recently, cancer research protocols have introduced clinical-stage spirooxindole-based MDM2 inhibitors. However, several studies reported tumor resistance to the treatment. This directed efforts to invest in designing various combinatorial libraries of spirooxindoles. Herein, we introduce new series of spirooxindoles via hybridization of the chemically stable core spiro[3*H*-indole-3,2′-pyrrolidin]-2(1*H*)-one and the pyrazole motif inspired by lead pyrazole-based p53 activators, the MDM2 inhibitor BI-0252 and promising molecules previously reported by our group. Single crystal X-ray diffraction analysis confirmed the chemical identity of a representative derivative. Fifteen derivatives were screened for cytotoxic activities via MTT assay against a panel of four cancer cell lines expressing wild-type p53 (A2780, A549, HepG2) and mutant p53 (MDA-MB-453). The hits were **8h** against A2780 (IC_50_ = 10.3 µM) and HepG2 (IC_50_ = 18.6 µM), **8m** against A549 (IC_50_ = 17.7 µM), and **8k** against MDA-MB-453 (IC_50_ = 21.4 µM). Further MTT experiments showed that **8h** and **8j** potentiated doxorubicin activity and reduced its IC_50_ by at least 25% in combinations. Western blot analysis demonstrated that **8k** and **8m** downmodulated MDM2 in A549 cells. Their possible binding mode with MDM2 were simulated by docking analysis.

## Introduction

**Figure 1:**
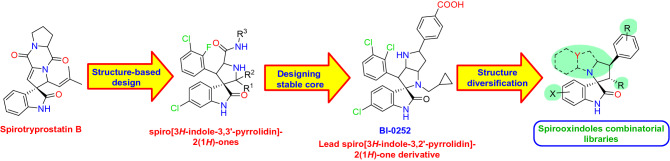
Development and optimization of spiro[3*H*-indole-3-2′-pyrrolidin]-2(1*H*)-ones MDM2 inhibitors.

The WHO 2020 report addressed the up-to-date global burden posed by cancer with more than 18.1 million cases estimated annually, expected to double by 2040^1^. Continuous drug discovery studies have been conducted to introduce novel medications targeting cancer hallmarks^2,3^. Evasion of apoptosis, being one of the main carcinogenesis hallmarks, has received considerable interest^4^. In this approach, the fundamental regulatory role of the tumor suppressor protein p53^5,6^ has been mirrored by various protocols for drugging p53 pathway to harness its apoptosis-inducing functions^7^. p53, the genome’s guardian, is a transcription factor normally regulating various pivotal genes responsible for DNA repair apoptosis and senescence^5,6^. Mechanistically, p53 primarily activates the pro-apoptotic proteins; PMAIP1 and PUMA that can also inhibit the mitochondrial anti-apoptotic proteins family, Mcl1 and Bcl2, thus inducing the apoptotic cascade^8,9^. Besides, direct p53 translocation to the mitochondria induces Bcl2 family proteins and triggers apoptosis^10,11^. Moreover, p53 induces caspase-independent programmed cell death^12^, cell senescence via p21, and associated opsonization signals^13^. p53 signals also activate caspases through mitochondrial cytochrome c release^14^. All these lines of evidence highlight the vital role of p53 in tumorigenesis. The levels of p53 and its intranuclear transcription activities are tightly controlled by its endogenous suppressor, E3 ubiquitin protein ligase MDM2 that normally conceals p53 transactivation domain^15^, inducing its degradation via ubiquitylation^16–18^, and transporting p53 to the cytoplasm. Consistent with this role, MDM2 is oncogenic when overexpressed^19^. In nearly 50% of tumors, p53 is mutated or deleted, whereas wild type p53 tumors loss their p53 functions due to MDM2 overexpression^20,21^ or amplification^22^. Accordingly, targeting the p53-MDM2 interplay for harnessing the apoptotic induction capacity of p53 has been proposed as an attractive therapeutic approach. Over the last decade, various drug discovery protocols have focused on validating the druggability of p53-MDM2 axis inhibition with substantial investment in direct MDM2 inhibition^23^. Initial hit-finding studies introduced about twenty classes of potent MDM2 inhibitors, of which seven optimized inhibitors were approved for clinical trials^24–32^. Among the promising clinical-stage inhibitors with optimized potency and pharmacokinetics, are spirooxindoles. This class was pioneered by Wang et al*.*^33,34^ via structure-based de novo design strategy, where the Trp23 indole of p53 that fills a hydrophobic cleft forming a key hydrogen bond interaction with the Leu54 residue of MDM2 was replaced by the oxindole ring. Then, an additional valency was introduced at the oxindole C3 for installing a spiro ring inspired by the natural anticancer spirotryprostatin B. Hence, the designed spiro[3*H*-indole-3,3′- pyrrolidin]-2(1*H*)-ones offered suitable vectors that address the remaining lipophilic MDM2 cavities (Phe19 and Leu26) in the p53 binding site. Several clinical-stage molecules were then developed e.g. Sanofi-Aventis SAR405838^35^, Hoffmann-La Roche RO2468^36^, and Daiichi Sankyo DS-3032b^37^. Modification of Wang’s scaffold by Boehringer Ingelheim introduced the chemically stable spiro[3*H*-indole-3,2′-pyrrolidin]-2(1*H*)-ones derivatives that aren’t prone to the epimerization observed in the spiro[3*H*-indole-3,3′-pyrrolidin]-2(1*H*)-ones with chiral C2^38^. Continuous optimization studies have led to the selective and orally bioavailable BI-0252 via incorporating fused systems inspired by natural products (Fig. 1)^39^.

Despite the advances in developing efficient clinical stage MDM2 inhibitors, some studies reported tumor resistance to the treatment^40–43^. This directed research protocols to invest more in designing various combinatorial libraries of spirooxindole-based MDM2 inhibitors, where the spirooxindole core has been modified at almost all possible positions^24,44–54^.

## Design rationale

**Figure 2:**
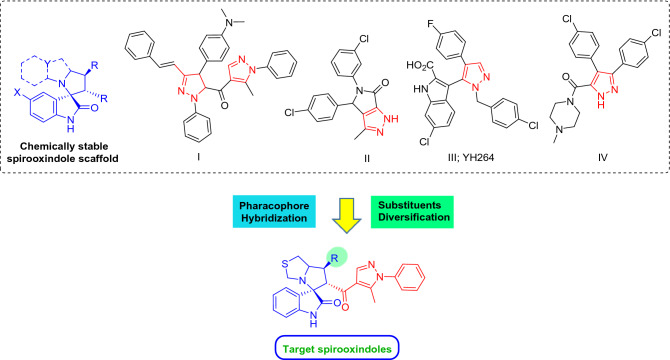
Design strategy of the target hybrid spirooxindoles.

In continuation to our previous studies^44–46,49–51^, we set our design strategy to synthesize new series of spirooxindole-based p53 activators via pharmacophoric hybridization approach. Herein, we utilized the spiro[3*H*-indole-3,2′-pyrrolidin]-2(1*H*)-one scaffold as promising chemically stable core for installing a pyrazole motif via a carbonyl spacer inspired by the potent lead pyrazole-based p53 activators^55–58^ (Fig. 2). The spiro ring was rationalized as tetrahydro-*1H,3H*-pyrrolo[1,2-*c*]thiazole to mimic that of the lead spirooxindole BI-0252 (Fig. 1) as well as other promising derivatives synthesized in our laboratory ^45^. Various substituents were introduced to the spiro ring to enrich the structure–activity relationship of the target series.

The target derivatives were synthesized via [3 + 2] cycloaddition of olefins, isatin, and the secondary amines, then preliminarily screened for their potential antiproliferative and apoptotic activities via MTT and annexin-V assays against human cancer cell lines expressing wild type or mutant p53. It is worth mentioning that the selected cancers are reported among the leading causes of cancer death^1^. The studied spirooxindoles were subjected to combination studies with doxorubicin, then western blot assays to evaluate their potential to modulate p53, and its targets MDM2, p21 and bax. Docking simulations of the studied derivatives were conducted into the MDM2 active site to predict their most probable binding modes and highlight their structural determinants of activity.

## Results and discussion

### Chemistry

**Figure 3:**
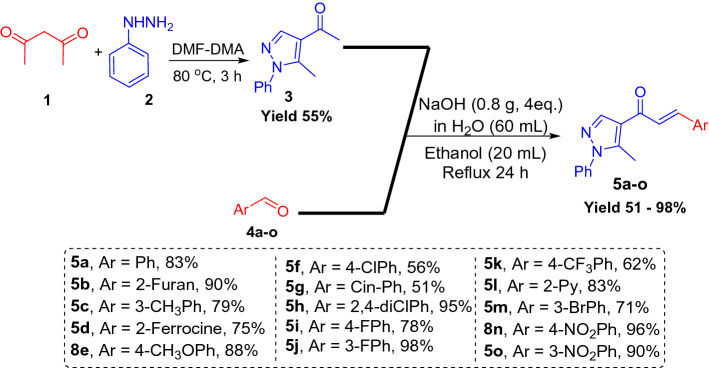
Chalcones synthesis-based pyrazole motif **5a–o.**

**Figure 4:**
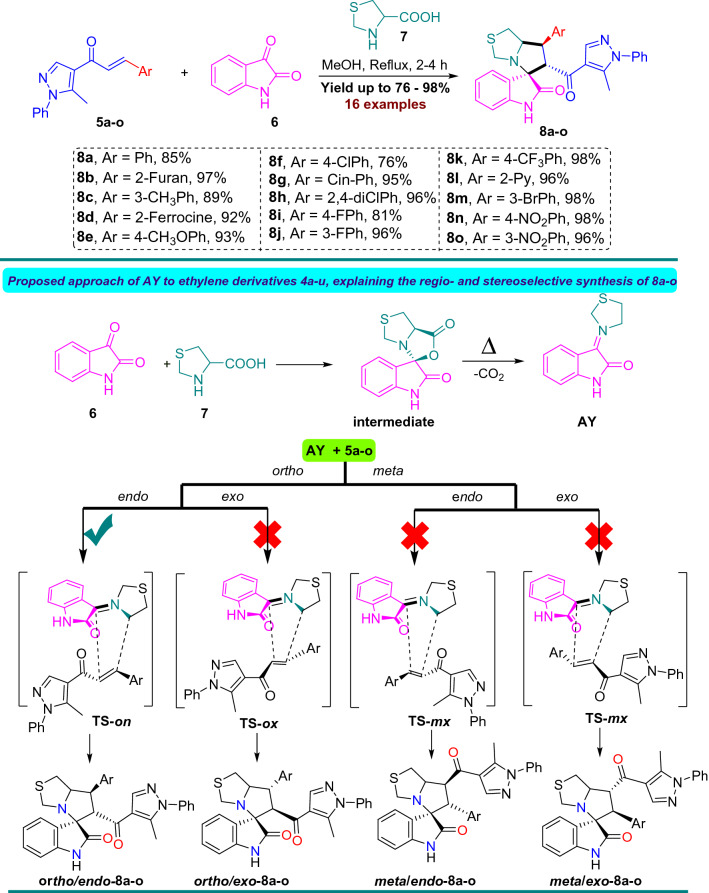
Synthesis and plausible mechanism route for spirooxindoles based pyrazole motif **8a–o**.

**Figure 5:**
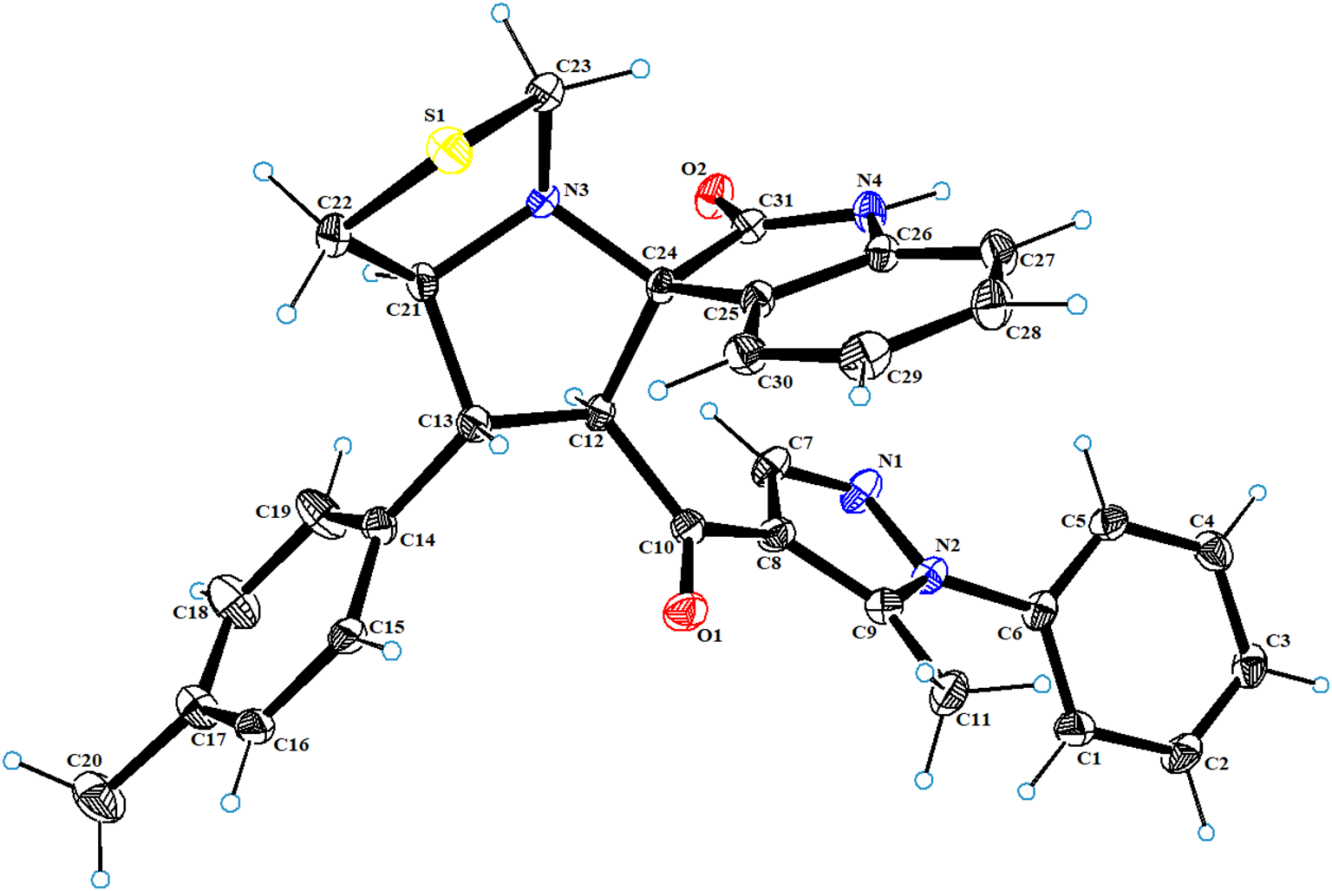
X-ray Crystal structure of** 8p.**

The new series of spirooxindole-based p53 activators via pharmacophoric hybridization approach were synthesized via one pot-multi component reaction including the dipolarphile (chalcone based pyrazole motif; Fig. 3) with the in situ generated azomethine yilde by reacting the isatin and l-thioproline in methanol under reflux for 2–4 h to afford the desired pharmacophoric spirooxindoles **8a–p** in high chemical yield 76–98% (Fig. 4). 16 examples of the pharmacophoric spirooxindoles having different electronic effects either electron donating or electron with releasing effects as well as heteroaryl rings and metallocene scaffold. The chemical structure is assigned based a set of spectrophotometric tools including 1D-NMR; MS and 2D-NMR analysis (spectral data provided in ESI; Figs. S1–S41). The absolute configuration of the pharmacophoric spirooxindoles was assigned based on single crystal X-ray diffraction analysis techniques. Based on the X-ray diffraction analysis approved that the reaction is stereoselective and diastereoselective which confirmed the plausible mechanistic pathway via* ortho/endo* [3 + 2] cycloaddition reaction experimentally favored. The molecular structure of **8p** (Fig. 5; Fig. S42 and molecular packing as indicated in Fig. S43; ESI) and crystal data provided in Tables S1 and S2 (ESI) confirmed the absolute configuration of the desired spirooxindoles.

### Anticancer evaluation

#### Antiproliferative activity

Among the synthesized series, the top spirooxindole derivatives were selected based on their records in preliminary filtration process employing docking of the compounds into the proposed target (MDM2 active site). Accordingly, **8a–o** were analyzed for their antiproliferative activity against a panel of four human cancer cell lines expressing wild-type p53, namely A2780 (ovarian), A549 (lung), HepG2 (liver) and mutant p53, namely MDA-MB-453 (breast; p53fs-c.991_993 + 189del), utilizing the MTT assay.

In general, our results (Table 1) showed that A2780 (IC_50_ = 10.3—47.3 µM, mean = 21.0 ± 8.2 µM, p < 0.05) and A549 (IC_50_ = 17.7–47.6 µM, mean = 23.2 ± 8.8 µM, p < 0.05) were on average more sensitive to the evaluated compounds than MDA-MB-453 (IC_50_ = 21.4–75.8 µM, mean = 35.4 ± 18.7 µM), while HepG2 cells showed only a trend (IC_50_ = 18.6–57.0 µM, mean = 24.1 ± 10.7 µM, p < 0.10). The presence of a mutant p53 protein in MDA-MB-453 cells likely confers a lower sensitivity to antiproliferative treatments.

**Table 1:** MTT assay of the spirooxindole derivatives **8a–o.**

Compound no.	A2780	A549	MDA-MB-453	HepG2
	IC_50_ (µM)^a^			
	18.6 ± 0.8^a^	21.5 ± 1.7	30.2 ± 3.2	19.8 ± 1.6
	29.4 ± 3.5	40.9 ± 10.7	22.4 ± 1.8	41.6 ± 6.7
	18.8 ± 1.5	19.4 ± 2.5	22.4 ± 1.4	20.3 ± 1.7
	21.7 ± 1.8	18.1 ± 1.8	22.4 ± 1.9	22.7 ± 2.5
	20.0 ± 0.9	24.4 ± 1.0	75.8 ± 23.5	21.5 ± 1.9
	19.8 ± 2.1	20.3 ± 1.1	24.8 ± 2.6	20.6 ± 1.3
	18.4 ± 1.3	18.2 ± 1.9	25.9 ± 2.6	18.8 ± 1.9
	10.3 ± 1.1	21.7 ± 1.9	23.8 ± 1.4	18.6 ± 2.4
	19.9 ± 1.4	18.6 ± 3.3	22.4 ± 1.1	19.9 ± 1.8
	17.9 ± 2.1	19.8 ± 0.8	22.4 ± 1.2	21.1 ± 2.0
	18.8 ± 0.8	19.3 ± 1.2	22.4 ± 1.3	19.4 ± 1.7
	47.3 ± 4.9	47.6 ± 5.1	22.4 ± 1.7	57.0 ± 8.7
	16.9 ± 1.1	17.7 ± 2.7	22.4 ± 1.4	18.9 ± 1.5
	18.0 ± 1.0	21.0 ± 0.9	22.4 ± 1.5	21.9 ± 1.8
	18.7 ± 1.5	19.5 ± 1.7	22.4 ± 1.6	20.1 ± 0.6

^a^Data represent the mean ± SD of 5–6 data.

Among the molecules under study, the 7'-pyridin-2-yl **(8l)** and furan-2yl **(8b)** spirooxindole derivatives had a very low antiproliferative activity in all cell lines, with their IC_50_ values being always higher (or equal) than 30 μM, that is the arbitrary value here considered to define the antiproliferative activity of a compound as pharmacologically relevant.

Considering the activity of our molecules within each cell line, the 7'-(2,4-dichlorophenyl) substituted spirooxindole derivative **8h** exhibited the highest antiproliferative activity against A2780 (IC_50_ = 10.3 ± 1.1 µM) and HepG2 cells (18.6 ± 2.4 µM). The 3-bromophenyl derivative **8m** was the more effective molecule on A549 (17.7 ± 2.7 µM) while the 4-(trifluoromethyl)phenyl derivative **8k** expresses the higher activity against MDA-MB-453 (21.4 ± 1.4 µM). Nearly all compounds recorded comparable IC_100_ (around 40 µM) except for the least active derivatives **8l, 8b**, and **8d** (range IC_100_: 46.6 ± 1.4–135.1 ± 5.3 µM). In Table S3 of ESI we reported for comparison the IC_50_ values for the standard drug (doxorubicin) used as control in the MTT assay.

**Figure 6:**
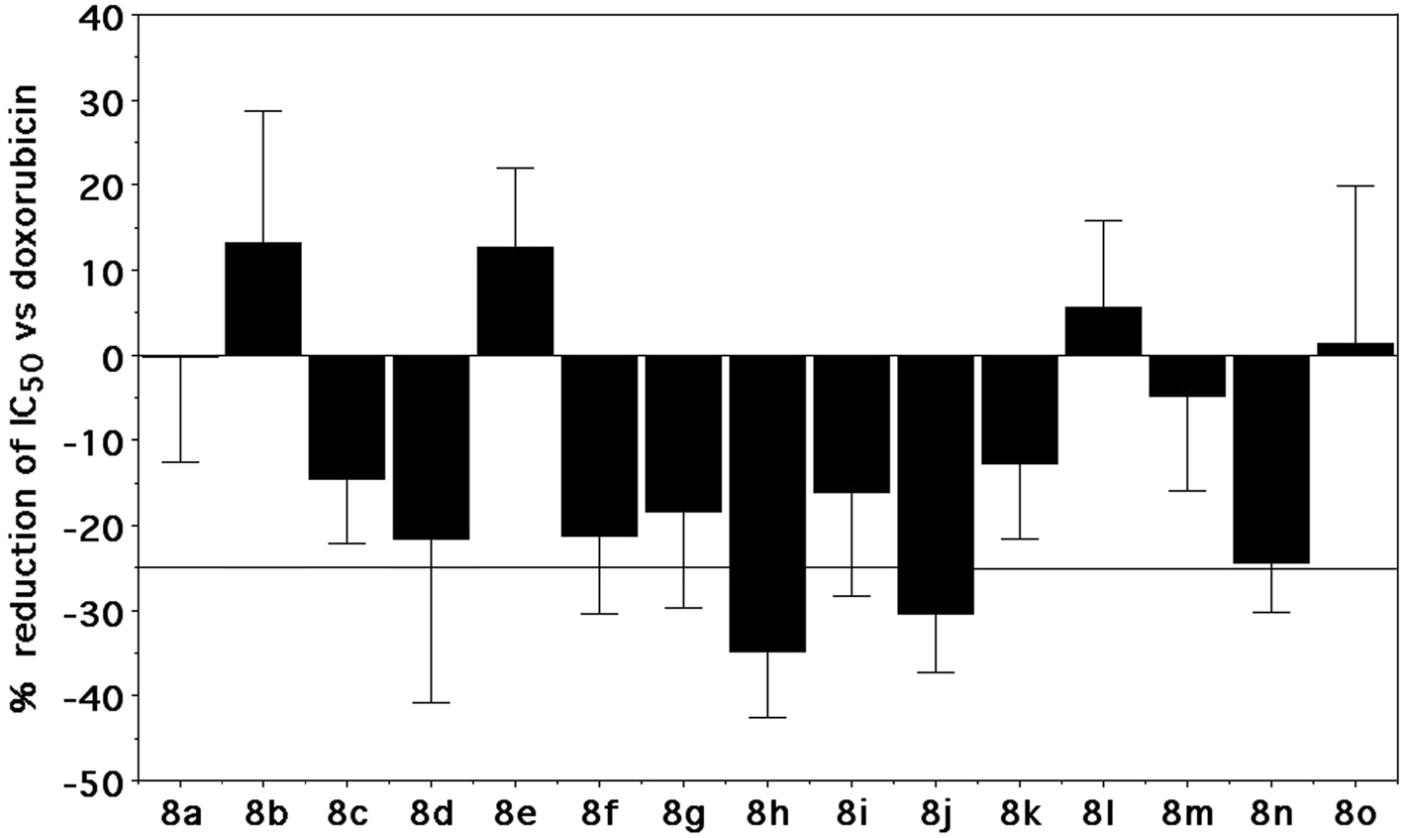
Antiproliferative activity after treatment of A549 cells with 6 h spirooxindoles followed by 72 h doxorubicin. Bars represent the mean ± SD of 3–7 data.

We also evaluated the antiproliferative activity when compounds were administered to A549 cells at 0.2 μM 6 h before doxorubicin treatment (see “M&M”). As showed in Fig. 6, only the 2,4-dichlorophenyl (**8h)** (p < 0.01) and 3-fluorophenyl (**8j)** (p < 0.01) derivatives potentiated doxorubicin antiproliferative activity, significantly reducing its IC_50_ by at least 25% (limit arbitrarily defined).

As for normal non-cancer cells, activated PBL (n = 3) were used and tested against the compounds most active in the two experimental model for the evaluation of antiproliferative activity, that is: **8h**, **8m**, **8k**, and **8j**. On average they show a lower antiproliferative activity against activated PBL with a mean IC_50_ of 54.7 ± 8.0.

On the whole our compounds **8h**, **8m**, **8k**, and **8j** were on average on activated PBL cells about 3.2, 2.7, 2.6, and 2.7 times, respectively, less active than on the tumor cell lines (mean IC_50_ values) used as target (Table 2).

**Table 2:** Antiproliferative activity of spirooxindole derivatives against normal PBL activated with PHA and recombinant IL-2.

Normal PBL	**8h**	**8m**	**8k**	**8j**
PBL A	53.6 ± 2.1^a^	48.8 ± 4.9	49.8 ± 3.2	54.0 ± 2.5
PBL B	57.4 ± 5.5	36.1 ± 14.7	55.3 ± 1.4	55.0 ± 3.3
PBL C	65.8 ± 23.2	68.1 ± 23.8	50.9 ± 1.2	61.2 ± 19.7

^a^Data representing the mean ± SD of 4–6 IC_50_ values (μM) obtained by the MTT assay.

#### Determination of apoptotic activity

**Figure 7:**
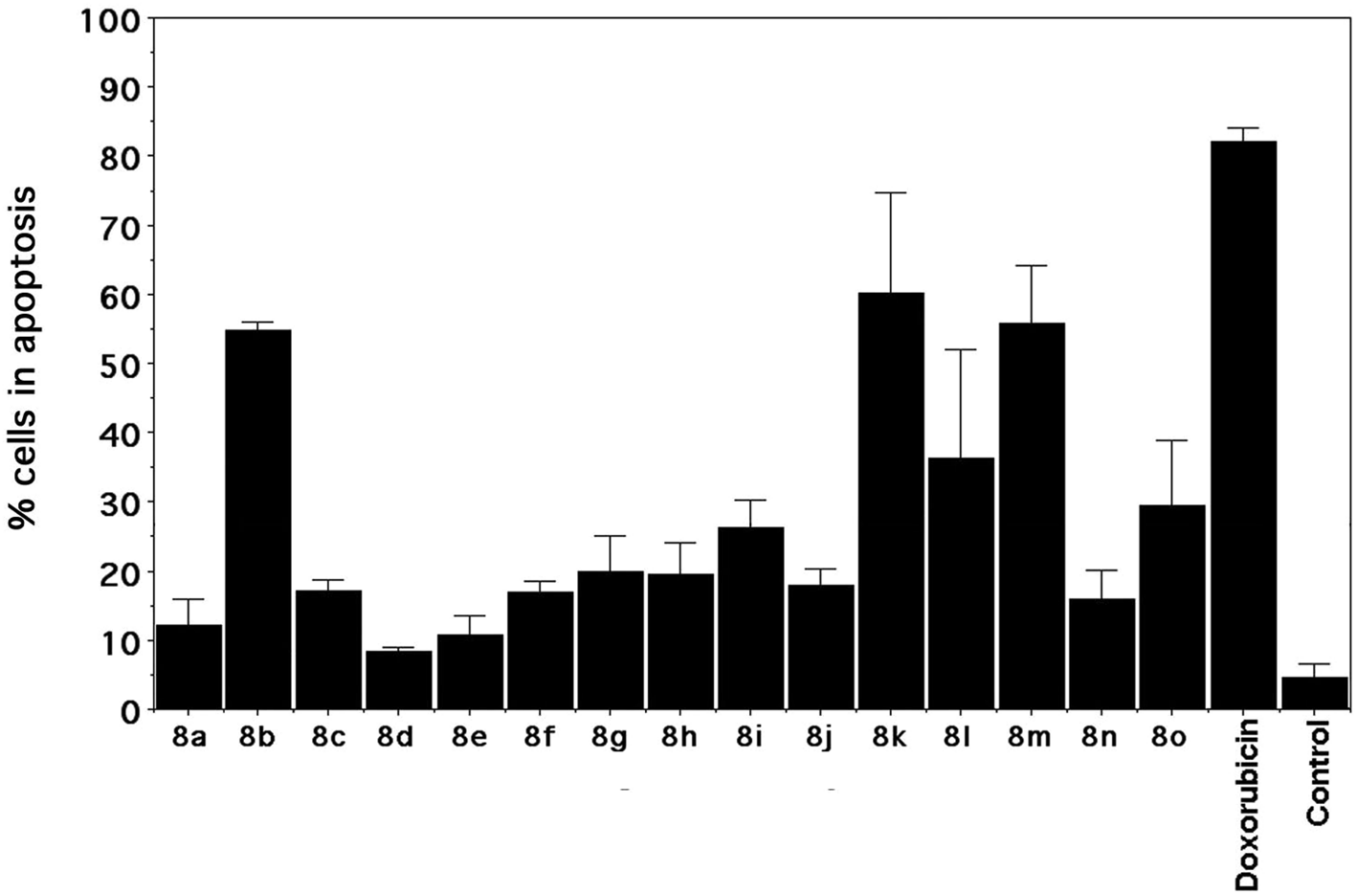
Bars represent the mean ± SD of 3 data. The induction of apoptosis was determined by double staining with Annexin-V-FITC and propidium iodide (PI).

In order to verify the triggering of apoptosis by the administration of our spirooxindole compounds we treated A549 cells with their specific IC_50_ values (compounds **8a**, **8c**, and **8e**), and IC_75_s (all compounds), as calculated by the MTT assay. As shown in Fig. 7 and Figure S44 ESI compounds **8t, 8k**, and **8m** displayed a highly significant apoptotic activity when administered at their specific IC_75_s, as waited for compounds able to inhibit MDM2 protein.

#### Expression of p53, MDM2, and p21

**Figure 8:**
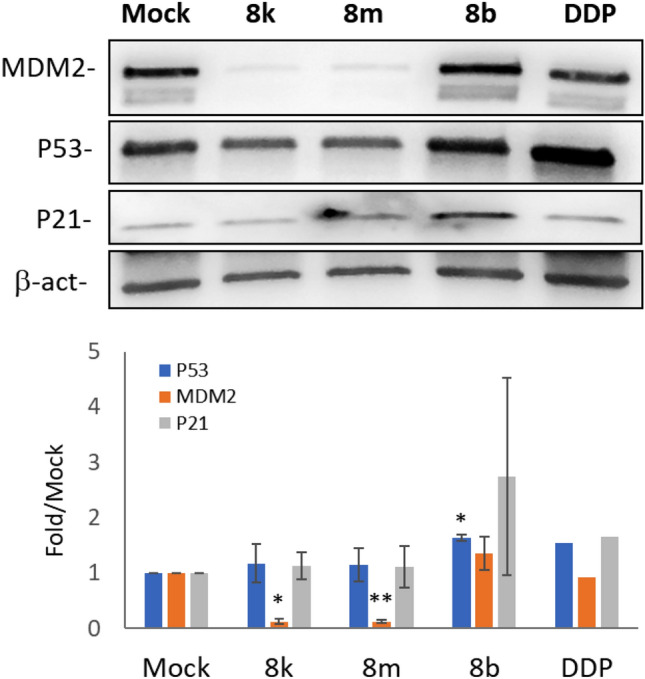
Modulation of p53 and relative targets following exposure to new spirooxindoles derivatives. A) Representative western blots showing the level of p53, MDM2, and P21 in A549 cells treated with 30 µM of **8k**, **8m**, **8b** and 14 µM DDP; β-actin was used to normalize; B) Histograms representing the amount of P53, MDM2 and P21 detected in A549. The levels of the different proteins were calculated as fold over the level of the same proteins found in Mock-treated cells (0.2% DMSO). Data from two independent experiments were obtained after chemiluminescence analysis of western blots and reported as mean ± SD (*p < 0.005; **p < 0.001, *t-*test).

Among the spirooxindoles under study, those showing the highest apoptotic activity (**8k**, **8m**, **8b**) were selected to evaluate their capacity to modulate p53 and its relative targets, namely p21 and MDM2. A549 cells carrying wild type p53 were treated at drug concentrations equivalent to IC_75_; cisplatin, was also employed as a control of p53 induction. Data on protein expression obtained by western blot (Fig. 8) showed that **8k** and **8m** did not induce any changes on p53 level but promoted a clear down modulation of MDM2 (all raw materials of immunoblots are provided in Figs. S45–S47 in ESI). On the contrary, the level of p53 significantly increased following **8b** exposure and, consequently, an increase of p21 and MDM2 proteins was observed. These data show that in A549 cells, **8b** possess some p53 activation capacity comparable to cisplatin; likely the apoptotic activity of **8k** and **8m** in this cell line is triggered by pathways not involving the activation of p53.

**Figure 9:**
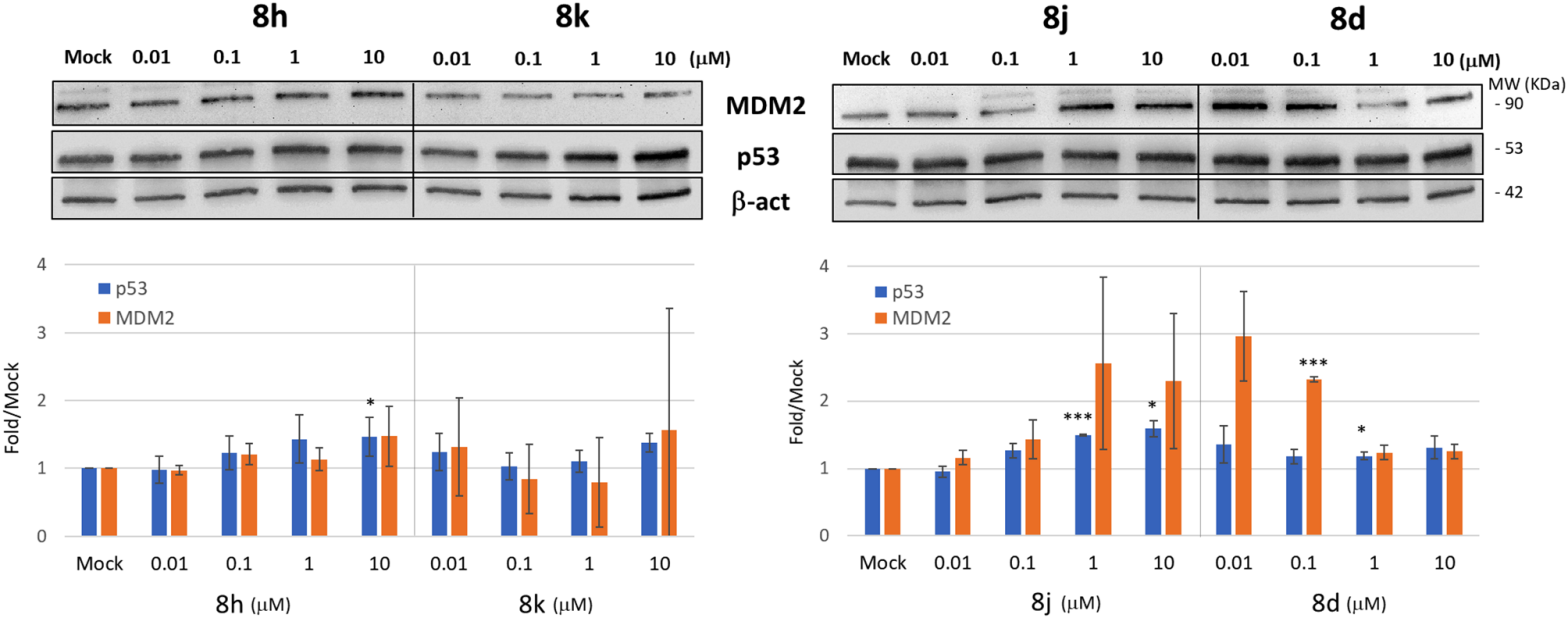
Modulation of p53 and MDM2 in A549 cells following exposure to spirooxindoles derivatives. (**A**) Representative western blot showing the level of p53 and MDM2 following treatment with increasing concentration of **8h, 8k, 8j** and **8d**, as indicated. Beta-actin was used to normalize. (**B**) Histograms representing the amount of P53 and MDM2 detected in A549 after treatment. The levels of the proteins were calculated as fold over the level of the same proteins found in Mock-treated cells (0.2% DMSO). Data were obtained after chemiluminescence analysis of western blots and reported as mean ± SD.

To investigate the effect of other molecules on p53 expression, we performed a dose–response experiment using a concentration range of 0.01, 0.1, 1, 10 µM for each derivative. Cell extracts from 24 h-treated A549 cells were prepared and analyzed by western blot (Fig. 9). In these conditions, **8h**, **8j** and **8d** were able to induce p53 only at high concentrations, while **8k** induced MDM2 down regulation at any concentration, confirming the previous observation (Fig. 8).

**Figure 10:**
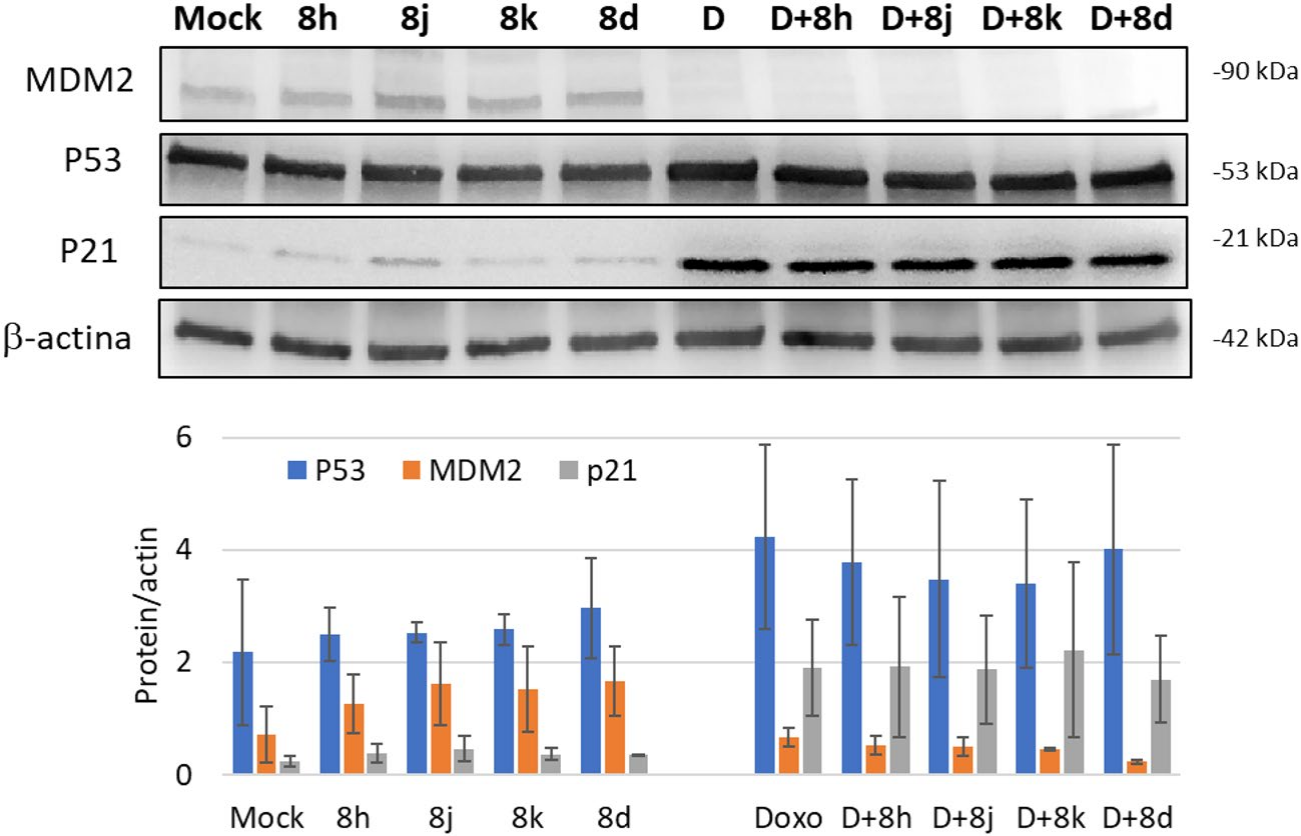
Representative western blot showing the level of p53, MDM2 and p21 following treatment with 0.2 µM of **8h, 8j, 8k, 8d** and 14 µM Doxorubicin alone or in combinations as indicated. Beta-actin was used to normalize. The histogram reports the values of proteins normalized with β-actin obtained from the chemiluminescence analysis of the western blot and reported as mean ± SD. D and Doxo stand for doxorubicin.

Next, we evaluated the levels of p53 and its targets in A549 cells treated with low concentration (0.2 µM) of selected derivatives **(8h, 8j, 8k** and **8d)** in combinations with doxorubicin (Fig. 10) in the conditions used as in Fig. 7. P53 and p21 did not increase in the presence of these compounds alone, while a slight but not significant MDM2 increase was observed, in particular for the **8j** molecule. Doxorubicin treatment alone clearly triggered the induction of p53 and p21, but not of MDM2, that instead strongly decreased. When compared to doxorubicin treatment alone, no further increases of p53, MDM2 and p21 proteins was observed in combined treatments (Fig. 10, lower panel). It has to be noted that the combined treatments were conducted at low drug concentrations, according to the experimental conditions used in antiproliferative and apoptotic activity assays. Treatments with a slightly higher drug concentration, but compatible with this experimental setting (0.4 μM), did not show difference. This suggests that likely high concentrations are needed to induce an effect on p53-MDM2 interplay.

The abundant increase of p21 after doxorubicin exposure is in keeping with the reported effect of doxorubicin on cell cycle arrest following an activation of p21 by p53 ^59,60^. However, the 6 h pre-treatment with **8h** and **8j** before doxorubicin administration, despite increasing the cytotoxicity compared to the single doxorubicin treatment (Fig. 6), did not trigger a further p53 and MDM2 increase, indicating that the MDM2-p53 axis is not involved in the cytotoxicity induced by combination treatment with **8h** or **8j** and doxorubicin.

Collectively, our data (summarized in Table 3) show that among the spirooxindoles here analyzed, **8h**, **8j**, **8k**, **8m** and **8b** appear the more promising, although their mode of action needs deeper investigation.

**Table 3:** Overall activity of selected spirooxindole derivatives.

Molecule	Cytotoxicity			Protein induction		
	MTT	Apoptosis	MTT combo	p53	MDM2	p21
**8h**	**	*	**	*	*	*
**8j**	*	*	**	*	*	*
**8k**	*	***				
**8m**	*	***				
**8b**		***		*	*	*
**8d**	*			*	*	

The molecules **8h** and **8j** which are able to exert antiproliferative activity, as well as to slightly trigger apoptosis, also induce p53, although not at high level and only at high concentrations. The **8b** is able to activate p53 and significantly trigger apoptosis. **8k** and **8m** did not induce p53; instead, they induced a down regulation of MDM2 and a considerable high level of apoptosis.

Thus, these results may suggest that **8h, 8j** and **8b** have some ability to act on the p53-MDM2 axis although further studies are necessary to define their mode of action. On the other hand, the pathways activated by the other molecules (i.e. **8k** and **8m**) are likely not strictly related to the inhibition of p53-MDM2 interaction. However, since they showed a high pro-apoptotic activity, may deserve further investigation.

### Docking simulations

**Figure 11:**
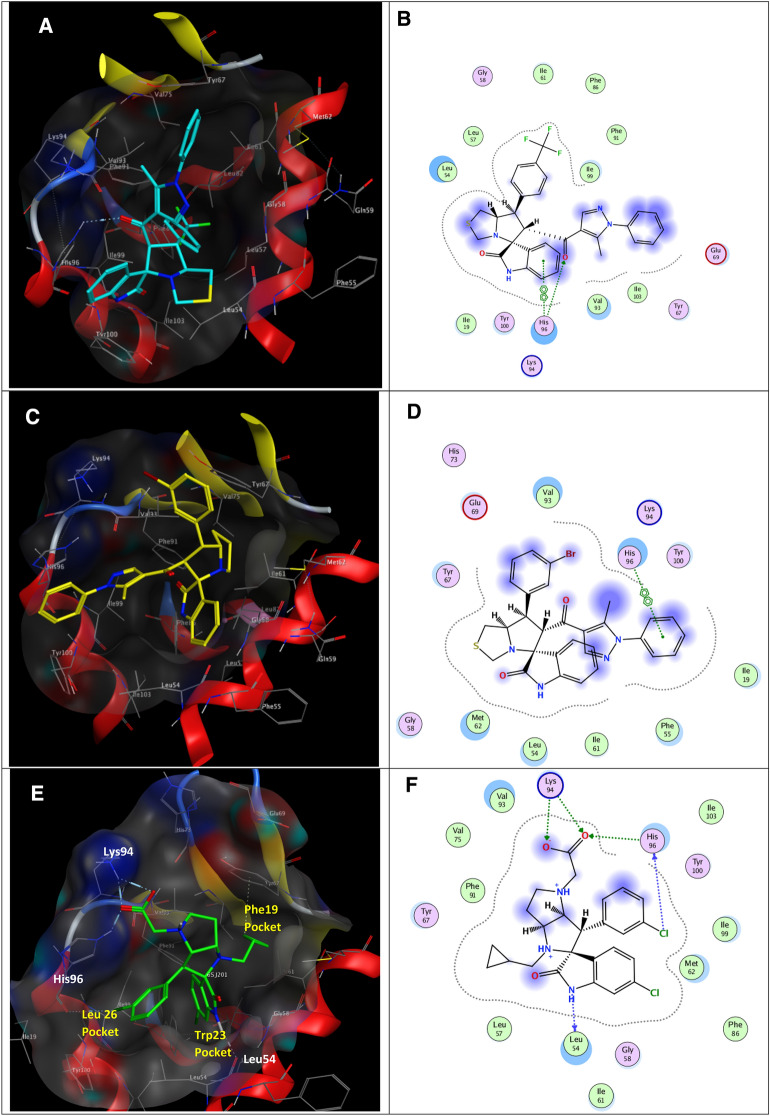
(**A**) 3D binding mode of **8k** (cyan sticks), (**B**) 2D binding mode of **8k**, (**C**) 3D binding mode of **8m** (yellow sticks), (**D**) 2D binding mode of **8m**, (**E**) 3D binding mode of the co-crystalized MDM2 inhibitor **6SJ** (green sticks), (**F**) 2D binding mode of **6SJ** in MDM2 (PBD ID: 5LAW^39^). The Leu26, Trp23, and Phe19 pockets of p53 are indicated in panel (**E**).

The X-ray crystal structure of E3 ubiquitin-protein ligase MDM2 co-crystallized with its reference spiro[3*H*-indole-3,2'-pyrrolidin]-2(1*H*)-one inhibitor **6SJ** was firstly retrieved from the RCSB PDB (PDB ID: 5LAW) ^39^ then prepared according to the default settings utilizing the “QuickPrep” MOE Version 2016.0802 module ^61^. The spirooxindole derivatives modulating MDM2 **(8k** and **8m)** were built in silico and energy minimized, then docked into the inhibitor’s binding site as previously reported ^44,45^. The docking simulations were conducted employing ‘Triangle Matcher’ as the placement method and ‘London dG’ scoring for calculating Gibbs energy for binding. The docking protocol was validated by redocking the co-crystallized inhibitor and reproducing the experimental interactions at acceptable RMSD. Docking results showed that the docked derivatives (Fig. 11) **8k** and **8m** accommodated into the MDM2 active site with binding scores (ΔG = −5.94, and −6.01 kcal/mol, respectively) comparable to the redocked reference inhibitor (ΔG = −7.80 kcal/mol). The best binding mode of **8k** displayed its indolinone ring posing π–π interactions with the active site key amino acid residue Lys94_(MDM2)_. Nevertheless, the carbonyl linking the heterocyclic core with the installed pyrazole appendage could accept hydrogen bond the same amino acid (Lys94_(MDM2)_). These interactions oriented the molecule so that the installed trifluoromethyl ring was obviously buried in the Phe19_(p53)_ pocket, the indolinone faced the His96_(MDM2)_ in the Leu26_(p53)_ pocket, and the spiro ring was close to Leu54_(MDM2)_ in the Trp23_(p53)_ pocket. On the other hand, **8m** occupied different spatial orientations, where the indolinone ring fitted into the Trp23_(p53)_ pocket resembling the reference’s core, directing the substituted spiro ring to the Phe19_(p53)_ pocket and extending the installed substituted pyrazole across the active site towards the His96_(MDM2)_ in the Leu26_(p53)_ pocket posing π–π interactions. In light of the results, it is postulated that the synthesized derivatives share some key interactions with the reference inhibitor. Installing substituted pyrazole did not hinder accommodation of the molecules into the active site when the spiro-indolinone core is appropriately substituted.

## Experimental

### Chemistry

The synthesis of chalcones **5a–p** required for the synthesis of the desired compounds have been provided in the supporting information.

### General procedure (GP1)

Chalcones **5a–p** (0.25 mmol), isatin **6** (0.25 mmol) and thioproline **7** (1.5 eq., 0.37 mmol) were dissolved in methanol (20 mL) and the reaction mixture was refluxed for 2–4 h. Finally, the products were isolated by flash column chromatography, using 1–3% MeOH/DCM to afford pyrazole spiro-oxindole **8a–p**.

#### 6'-(5-Methyl-1-phenyl-1*H*-pyrazole-4-carbonyl)-7'-phenyl-1',6',7',7a'-tetrahydro-3'*H*-spiro[indoline-3,5'-pyrrolo[1,2-c]thiazol]-2-one (8a)


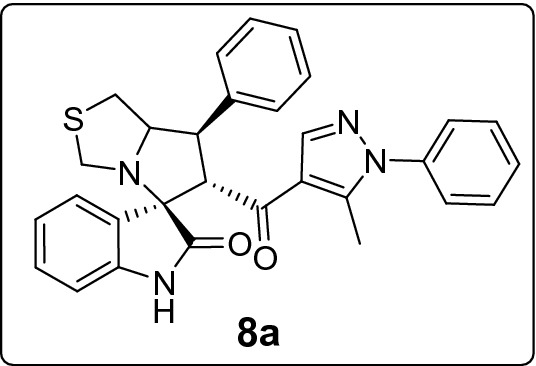
Following the general procedure (**GP1**), chalcone **5a** (72 mg, 0.25 mmol), isatin (**6)** (47 mg, 0.25 mmol) and thioproline (**7**) (50 mg, 0.37 mmol) in methanol (20 mL) were reacted to yield solid compound **8a**; Yield (108 mg, 85%); m.p: 229–230 ºC; ^1^H-NMR (700 MHz, DMSO_-_*d*_6_): *δ*(ppm) = 10.50 (s, 1H NH), 7.88 (s, 1H, Ar–H), 7.52 (d, *J* = 8.3 Hz, 2H, Ar–H), 7.50 (t, *J* = 7.1 Hz, 2H, Ar–H), 7.47–7.43 (m, 2H, Ar–H), 7.35 (t, *J* = 7.6 Hz, 2H, Ar–H), 7.32 (d, *J* = 7.5 Hz, 2H, Ar–H), 7.24 (t, *J* = 7.5 Hz, 1H, Ar–H), 7.14 (t, *J* = 7.7 Hz, 1H, Ar–H), 6.93 (t, *J* = 7.5 Hz, 1H, Ar–H), 6.63 (d, *J* = 7.7 Hz, 1H, Ar–H), 4.50 (d, *J* = 11.7 Hz, 1H, CHCO), 4.15–4.09 (m, 1H, NCH), 3.86 (t, *J* = 10.6 Hz, 1H, NCHCH), 3.73 (d, *J* = 10.2 Hz, 1H, NCH_2(a)_), 3.37 (d, *J* = 10.2 Hz, 1H, NCH_2(b)_), 3.06–2.97 (m, 2H, SCH_2_), 1.81 (s, 3H, CH_3_); ^13^C-NMR (176 MHz, DMSO_-_*d*_6_): δ(ppm) = 190.25 (CO), 178.66 (CO), 142.58, 142.20, 140.86, 139.53, 137.97, 129.70, 129.31, 128.80, 128.76, 128.21, 128.13, 127.10, 125.23, 123.30, 120.85, 120.29, 109.36, 74.62, 73.83, 64.16, 53.58, 50.29, 35.89, 11.07 (CH_3_); IR (KBr, cm^–1^) ν_max_ = 3260, 3058, 2873, 1733, 1700, 1665, 1616, 1596, 1540, 1502, 1469, 1453, 1396, 1326, 1287, 1224, 1188, 1170, 1122, 1069, 1026, 933, 812, 770, 753, 724, 700, 692, 598, 520; [Anal. Calcd. for C_30_H_26_N_4_O_2_S: C, 71.12; H, 5.17; N, 11.06; Found: C, 70.94; H, 5.09; N, 10.91]; LC/MS (ESI, *m/z*): found 507.3 [M + H]^+^, exact mass 506.18 for C_30_H_26_N_4_O_2_S.

#### 7'-(Furan-2-yl)-6'-(5-methyl-1-phenyl-1*H*-pyrazole-4-carbonyl)-3',6',7',7a'-tetrahydro-1'*H*-spiro[indoline-3,5'-pyrrolo[1,2-c]thiazol]-2-one (8b)


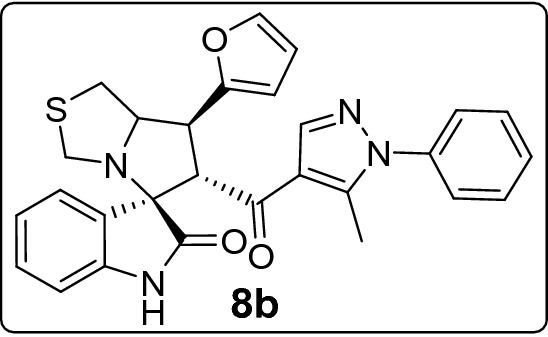
Following the general procedure (**GP1**), chalcone **5b** (70 mg, 0.25 mmol), isatin (**6)** (47 mg, 0.25 mmol) and thioproline (**7**) (50 mg, 0.37 mmol) in methanol (20 mL) were reacted to yield solid compound **8b**; Yield (121 mg, 97%); m.p: 227–228 ºC; ^1^H-NMR (400 MHz, DMSO_-_*d*_6_): δ(ppm) = 10.47 (s, 1H, NH), 7.87 (s, 1H, Ar–H), 7.61 (t, *J* = 1.3 Hz, 1H, Ar–H), 7.56–7.47 (m, 3H, Ar–H), 7.39–7.33 (m, 3H, Ar–H), 7.14 (td, *J* = 7.7, 1.2 Hz, 1H, Ar–H), 6.91 (td, *J* = 7.6, 1.1 Hz, 1H, Ar–H), 6.62 (d, *J* = 7.7 Hz, 1H, Ar–H), 6.40 (d, *J* = 1.4 Hz, 2H, Ar–H), 4.45 (d, *J* = 11.8 Hz, 1H, CHCO), 4.11 (dt, *J* = 9.3, 4.6 Hz, 1H, NCH), 3.95 (dd, *J* = 11.8, 9.4 Hz, 1H, NCHCH), 3.71 (d, *J* = 10.3 Hz, 1H, NCH_2(a)_), 3.35 (d, *J* = 10.3 Hz, 1H, NCH_2(b)_), 3.18 – 3.11 (m, 2H, SCH_2_), 1.84 (s, 3H, CH_3_); ^13^C-NMR (176 MHz, DMSO_-_*d*_6_): δ(ppm) = 189.94 (CO), 178.49 (CO), 152.75, 142.73, 142.45, 142.22, 140.86, 138.00, 129.87, 129.40, 128.90, 128.15, 125.29, 123.06, 120.91, 120.08, 110.62, 109.44, 106.61, 73.64, 71.86, 61.42, 53.71, 43.63, 36.36, 11.08 (CH_3_); IR (KBr, cm^–1^) ν_max_ = 3237, 2879, 2833, 1733, 1667, 1617, 1597, 1536, 1503, 1470, 1399, 1330, 1207, 1180, 1115, 1071, 1010, 925, 807, 695, 661, 599; [Anal. Calcd. for C_28_H_24_N_4_O_3_S: C, 67.72; H, 4.87; N, 11.28; Found: C, 67.79; H, 4.71; N, 11.23]; LC/MS (ESI, *m/z*): found 497.2 [M + H]^+^_;_ exact mass 496.16 for C_28_H_24_N_4_O_3_S.

#### 6'-(5-Methyl-1-phenyl-1*H*-pyrazole-4-carbonyl)-7'-(m-tolyl)-1',6',7',7a'-tetrahydro-3'*H*-spiro[indoline-3,5'-pyrrolo[1,2-c]thiazol]-2-one (8c)


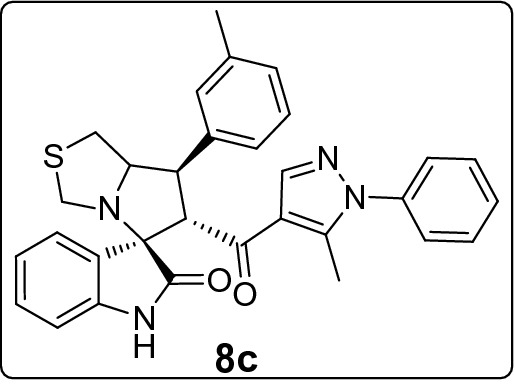
Following the general procedure (**GP1**), chalcone **5c** (76 mg, 0.25 mmol), isatin (**6)** (47 mg, 0.25 mmol) and thioproline (**7**) (50 mg, 0.37 mmol) in methanol (20 mL) were reacted to yield solid compound **8c**; Yield (116 mg, 89%); m.p: 128–130 ºC; ^1^H-NMR (700 MHz, DMSO_-_*d*_6_): δ(ppm) = 10.47 (s, 1H, NH), 7.87 (s, 1H, Ar–H), 7.51 (t, *J* = 7.5 Hz, 2H, Ar–H), 7.48–7.45 (t, *J* = 7.5 Hz, 1H, Ar–H), 7.44 (d, *J* = 7.6 Hz, 1H, Ar–H), 7.33 (d, *J* = 9.0 Hz, 3H, Ar–H), 7.30 (d, *J* = 7.8 Hz, 1H, Ar–H), 7.24 (t, *J* = 7.6 Hz, 1H, Ar–H), 7.14 (t, *J* = 7.7 Hz, 1H, Ar–H), 7.06 (d, *J* = 7.5 Hz, 1H, Ar–H), 6.92 (t, *J* = 7.6 Hz, 1H, Ar–H), 6.62 (d,* J* = 7.6 Hz, 1H, Ar–H), 4.47 (d, *J* = 11.8 Hz, 1H, CHCO), 4.09 (ddd, *J* = 9.4, 6.4, 3.0 Hz, 1H, NCH), 3.80 (dd, *J* = 11.8, 9.4 Hz, 1H, NCHCH), 3.72 (d, *J* = 10.2 Hz, 1H, NCH_2(a)_), 3.36 (d, *J* = 10.2 Hz, 1H, NCH_2(b)_), 3.02 (dd, *J* = 11.4, 6.4 Hz, 1H, SCH_2(a)_), 2.98 (dd, *J* = 11.4, 3.0 Hz, 1H, SCH_2(b)_), 2.32 (s, 3H, CH_3_), 1.80 (s, 3H, CH_3_); ^13^C-NMR (176 MHz, DMSO_-_*d*_6_): δ(ppm) = 190.31 (CO), 178.69 (CO), 142.56, 142.19, 140.91, 139.45, 137.99, 137.90, 129.72, 129.35, 128.83, 128.76, 128.66, 128.21, 127.80, 125.25, 125.18, 123.31, 120.85, 120.31, 109.36, 74.67, 73.88, 64.08, 53.65, 50.28, 35.92, 21.08 (CH_3_), 11.06 (CH_3_); IR (KBr, cm^–1^) ν_max_ = 3245, 3058, 2916, 1733, 1666, 1617, 1597, 1536, 1503, 1470, 1393, 1328, 1283, 1229, 1179, 1115, 1071, 978, 935, 838, 764, 725, 695, 659, 579; [Anal. Calcd. for C_31_H_28_N_4_O_2_S: C, 71.51; H, 5.42; N, 10.76; Found: C, 71.46; H, 5.49; N, 10.71]; LC/MS (ESI, *m/z*): found 521.3 [M + H]^+^, exact mass 520.19 for C_31_H_28_N_4_O_2_S.

#### 7'-(Ferrocin-2-yl)-6'-(5-methyl-1-phenyl-1*H*-pyrazole-4-carbonyl)-3',6',7',7a'-tetrahydro-1'*H*-spiro[indoline-3,5'-pyrrolo[1,2-c]thiazol]-2-one (8d)


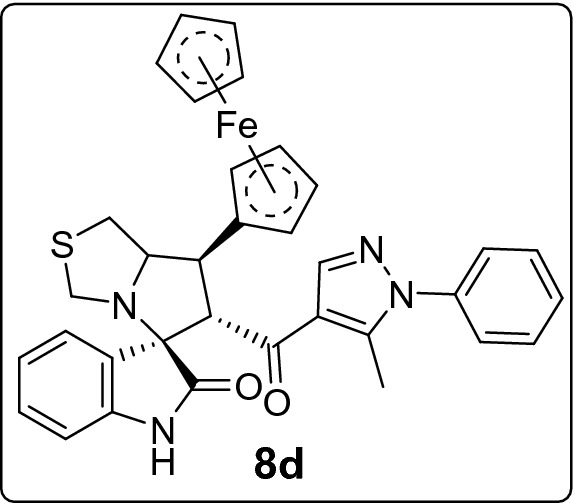
Following the general procedure (**GP1**), chalcone **5d** (107 mg, 0.25 mmol), isatin (**6)** (47 mg, 0.25 mmol) and thioproline (**7**) (50 mg, 0.37 mmol) in methanol (20 mL) were reacted to yield solid compound **8d**; Yield (148 mg, 92%); m.p: 152–153 ºC; ^1^H-NMR (400 MHz, DMSO_-_*d*_6_): δ(ppm) = 10.50 (s, 1H, NH), 7.87 (s, 1H, Ar–H), 7.58–7.49 (m, 3H, Ar–H), 7.41 (d, *J* = 1.5 Hz, 1H, Ar–H), 7.39 (dd, *J* = 1.9, 1.0 Hz, 1H, Ar–H), 7.25 (d, *J* = 7.4 Hz, 1H, Ar–H), 7.13 (td, *J* = 7.7, 1.2 Hz, 1H, Ar–H), 6.90 (td, *J* = 7.6, 1.1 Hz, 1H, Ar–H), 6.62 (d, *J* = 7.7 Hz, 1H, Ar–H), 4.52 (d, *J* = 10.7 Hz, 1H, CHCO), 4.34 (s, 5H, Cp-H), 4.33–4.29 (m, 1H, Cp-H), 4.21 (dp, *J* = 3.8, 1.2 Hz, 2H, Cp-H), 4.13 (td, *J* = 2.4, 1.3 Hz, 1H, NCH), 3.81–3.78 (m, 1H, Cp-H), 3.76 (d, *J* = 9.7 Hz, 1H, NCH_2(a)_), 3.59 (dd, *J* = 10.7, 7.9 Hz, 1H, NCHCH), 3.38 (dd, *J* = 10.9, 6.7 Hz, 1H, SCH_2(a)_), 3.33 (s, 1H, NCH_2(b)_), 3.10 (dd, *J* = 10.8, 4.7 Hz, 1H, SCH_2(b)_), 1.93 (s, 3H, CH_3_); ^13^C-NMR (176 MHz, DMSO_-_*d*_6_): δ(ppm) = 181.71 (CO), 179.43 (CO), 143.39, 142.36, 140.82, 138.21, 130.09, 129.87, 129.43, 128.68, 125.65, 123.82, 121.48, 120.66, 109.78, 90.14, 74.39, 74.25, 68.89, 67.93, 67.79, 67.66, 67.00, 65.89, 52.99, 42.89, 38.29, 11.52 (CH_3_); IR (KBr, cm^–1^) ν_max_ = 3251, 3089, 2835, 1733, 16,667, 1617, 1597, 1535, 1502, 1469, 1394, 1328, 1287, 1220, 1179, 1105, 1001, 935, 922, 811, 752, 695, 659, 488; [Anal. Calcd. for C_36_H_36_FeN_4_O_2_S: C, 67.08; H, 5.63; N, 8.69; Found: C, 66.94; H, 5.76; N, 8.54]; LC/MS (ESI, *m/z*): found 645.2 [M + H]^+^_;_ exact mass 644.19 for C_36_H_36_FeN_4_O_2_S.

#### 7'-(4-Methoxyphenyl)-6'-(5-methyl-1-phenyl-1*H*-pyrazole-4-carbonyl)-1',6',7',7a'-tetrahydro-3'*H*-spiro[indoline-3,5'-pyrrolo[1,2-c]thiazol]-2-one (8e)


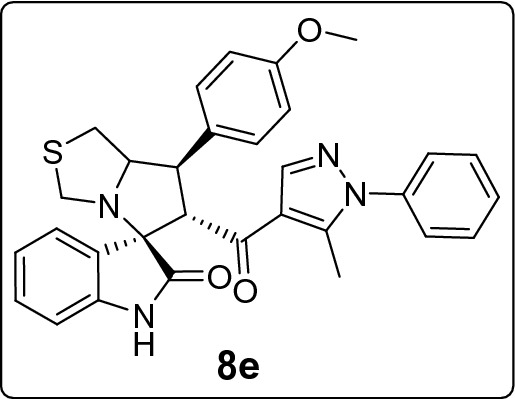
Following the general procedure (**GP1**), chalcone **5e** (80 mg, 0.25 mmol), isatin (**6)** (47 mg, 0.25 mmol) and thioproline (**7**) (50 mg, 0.37 mmol) in methanol (20 mL) were reacted to yield solid compound **8e**; Yield (125 mg, 93%); m.p: 109–110 ºC; ^1^H-NMR (400 MHz, DMSO_-_*d*_6_): δ(ppm) = 10.46 (s, 1H, NH), 7.86 (s, 1H, Ar–H), 7.54–7.47 (m, 3H, Ar–H), 7.46–7.41 (m, 3H, Ar–H), 7.36–7.31 (m, 2H, Ar–H), 7.14 (t, *J* = 7.7 Hz, 1H, Ar–H), 6.91 (d, *J* = 8.8 Hz, 3H, Ar–H), 6.62 (d, *J* = 7.3 Hz, 1H, Ar–H), 4.42 (d, *J* = 11.8 Hz, 1H, CHCO), 4.05–4.10 (m, 1H, NCH), 3.81 (dd, *J* = 9.4, 4.2, 1H, NCHCH), 3.73 (d, *J* = 10.2 Hz, 1H, NCH_2(a)_), 3.72 (s, 3H, OCH_3_), 3.36 (d, *J* = 10.2 Hz, 1H, NCH_2(b)_), 3.07–2.94 (m, 2H, SCH_2_), 1.83 (s, 3H, CH_3_); ^13^C-NMR (176 MHz, DMSO_-_*d*_6_): δ(ppm) = 190.28 (CO), 178.65 (CO), 158.33, 142.53, 142.15, 140.84, 137.97, 131.23, 129.66, 129.32, 129.10, 128.80, 128.23, 125.22, 123.31, 120.80, 120.35, 114.14, 109.32, 74.56, 73.79, 64.18, 55.06 (OCH_3_), 53.63, 49.62, 35.86, 11.08 (CH_3_); IR (KBr, cm^–1^) ν_max_ = 3245, 2915, 2836, 1733, 1665, 1615, 1597, 1539, 1513, 1503, 1470, 1394, 1328, 1248, 1177, 1118, 1070, 1032, 979, 935, 872, 830, 807, 764, 752, 695, 658, 606, 564, 542; [Anal. Calcd. for C_31_H_28_N_4_O_3_S: C, 69.38; H, 5.26; N, 10.44; Found: C, 69.29; H, 5.32; N, 10.48]; LC/MS (ESI, *m/z*): found 537.3 [M + H]^+^, exact mass 536.19 for C_31_H_28_N_4_O_3_S.

#### 7'-(4-Chlorophenyl)-6'-(5-methyl-1-phenyl-1H-pyrazole-4-carbonyl)-1',6',7',7a'-tetrahydro-3'H-spiro[indoline-3,5'-pyrrolo[1,2-c]thiazol]-2-one (8f)


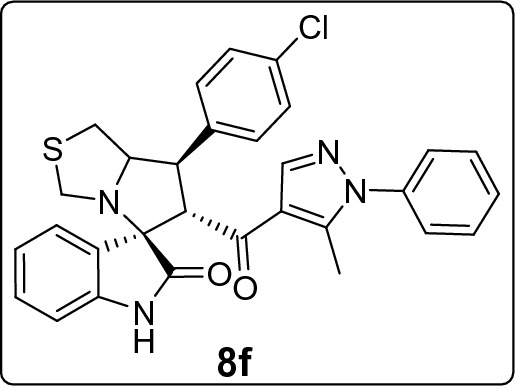
Following the general procedure (**GP1**), chalcone **5f.** (81 mg, 0.25 mmol), isatin (**6)** (47 mg, 0.25 mmol) and thioproline (**7**) (50 mg, 0.37 mmol) in methanol (20 mL) were reacted to yield solid compound **8f**; Yield (103 mg, 76%); m.p: 122–123 ºC; ^1^H-NMR (400 MHz, DMSO_-_*d*_6_): δ(ppm) = 10.47 (s, 1H, NH), 7.88 (s, 1H, Ar–H), 7.57 (d, *J* = 8.6 Hz, 2H, Ar–H), 7.54–7.46 (m, 3H, Ar–H), 7.44 (d, *J* = 7.6 Hz, 1H, Ar–H), 7.42–7.39 (m, 2H, Ar–H), 7.35–7.31 (m, 2H, Ar–H), 7.14 (t, *J* = 7.7 Hz, 1H, Ar–H), 6.92 (t, *J* = 7.0 Hz, 1H, Ar–H), 6.62 (d, *J* = 7.2 Hz, 1H, Ar–H), 4.46 (d, *J* = 11.7 Hz, 1H, CHCO), 4.09 (m, 1H, NCH), 3.86 (dd, *J* = 11.7, 9.5 Hz, 1H, NCHCH), 3.72 (d, *J* = 10.1 Hz, 1H, NCH_2(a)_), 3.36 (d, *J* = 10.1 Hz, 1H, NCH_2(b)_), 3.06 – 2.97 (m, 2H, SCH_2_), 1.81 (s, 3H, CH_3_); ^13^C-NMR (176 MHz, DMSO_-_*d*_6_): δ(ppm) = 190.27 (CO), 178.64 (CO), 142.66, 142.24, 140.99, 138.53, 138.01, 131.80, 130.18, 129.85, 129.42, 128.92, 128.75, 128.21, 125.30, 123.24, 120.96, 120.25, 109.47, 74.40, 73.86, 64.16, 53.63, 49.64, 35.80, 11.09 (CH_3_); IR (KBr, cm^–1^) ν_max_ = 3233, 2922, 2851, 1730, 1667, 1617, 1597, 1537, 1503, 1492, 1470, 1454, 1395, 1329, 1231, 1179, 1089, 1013, 933, 807, 764, 683, 695, 658, 607, 537; [Anal. Calcd. for C_30_H_25_ClN_4_O_2_S: C, 66.60; H, 4.66; N, 10.36; Found: C, 66.68; H, 5.71; N, 10.24]; LC/MS (ESI, *m/z*): found 541.2 [M(_35_Cl) + H]^+^, 543.2 [M(_37_Cl) + H]^+^_;_ exact mass 540.14 for C_30_H_25_ClN_4_O_2_S.

#### 6'-(5-Methyl-1-phenyl-1*H*-pyrazole-4-carbonyl)-7'-((*E*)-styryl)-1',6',7',7a'-tetrahydro-3'*H*-spiro[indoline-3,5'-pyrrolo[1,2-c]thiazol]-2-one (8 g)


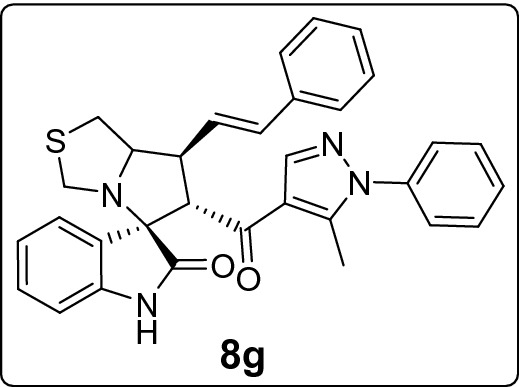
Following the general procedure (**GP1**), chalcone **5 g** (79 mg, 0.25 mmol), isatin (**6)** (47 mg, 0.25 mmol) and thioproline (**7**) (50 mg, 0.37 mmol) in methanol (20 mL) were reacted to yield solid compound **8g**; Yield (127 mg, 95%); m.p: 144–145 ºC; ^1^H-NMR (400 MHz, DMSO_-_*d*_6_): δ(ppm) = 10.40 (s, 1H, NH), 7.89 (s, 1H, Ar–H), 7.55–7.51 (m, 2H, Ar–H), 7.50–7.47 (m, 1H, Ar–H), 7.47–7.44 (m, 2H, Ar–H), 7.40 (d, *J* = 7.1 Hz, 1H, Ar–H), 7.38–7.37 (m, 1H, Ar–H), 7.36–7.35 (m, 1H, Ar–H), 7.34–7.30 (m, 2H, Ar–H), 7.22 (t, *J* = 7.3 Hz, 1H, Ar–H), 7.14 (t, *J* = 7.7 Hz, 1H, Ar–H), 6.92 (t, *J* = 7.5 Hz, 1H, Ar–H), 6.46 (d, *J* = 15.9, 1H, CH = CH), 6.42 (d, *J* = 6.8 Hz, 1H, Ar–H), 6.44 (dd, *J* = 15.9, 8.3 Hz, 1H, CH = CH), 4.26 (d, *J* = 11.5 Hz, 1H, CHCO), 4.06–4.00 (m, 1H, NCH), 3.73 (d, *J* = 10.4 Hz, 1H, NCH_2(a)_), 3.45 (dd, *J* = 12.4, 8.8 Hz, 1H, NCHCH), 3.35 (d, *J* = 10.4 Hz, 1H, NCH_2(b)_), 3.13 (dd, *J* = 11.5, 6.3 Hz, 1H, SCH_2_), 1.84 (s, 3H, CH_3_); ^13^C-NMR (176 MHz, DMSO_-_*d*_6_): δ(ppm) = 190.55 (CO), 178.76 (CO), 142.49, 142.18, 141.09, 138.06, 136.72, 132.28, 129.70, 129.36, 128.82, 128.79, 128.61, 128.29, 127.50, 126.27, 125.27, 123.26, 120.76, 120.46, 109.34, 74.12, 72.39, 62.52, 54.10, 49.06, 36.00, 11.04 (CH_3_); IR (KBr, cm^–1^) ν_max_ = 3240, 3057, 3025, 2921, 2851, 1734, 1668, 1617, 1597, 1539, 1502, 1470, 1394, 1328, 1228, 1179, 1116, 1072, 966, 933, 807, 751, 722, 693, 659, 603, 536, 487; [Anal. Calcd. for C_32_H_28_N_4_O_2_S: C, 72.16; H, 5.30; N, 10.52; Found: C, 72.29; H, 5.17; N, 10.47]; LC/MS (ESI, *m/z*): found 533.3 [M + H]^+^_;_ exact mass 532.19 for C_32_H_28_N_4_O_2_S.

#### 7'-(2,4-Dichlorophenyl)-6'-(5-methyl-1-phenyl-1*H*-pyrazole-4-carbonyl)-1',6',7',7a'-tetrahydro-3'*H*-spiro[indoline-3,5'-pyrrolo[1,2-c]thiazol]-2-one (8h)


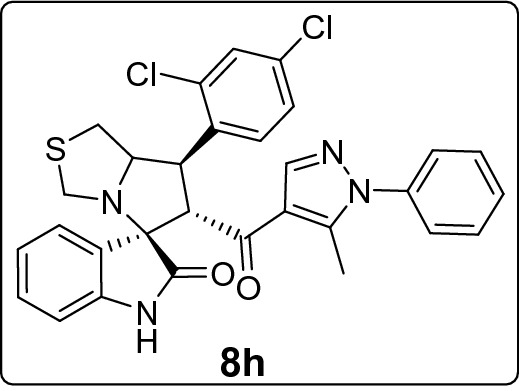
Following the general procedure (**GP1**), chalcone **5 h** (89 mg, 0.25 mmol), isatin (**6)** (47 mg, 0.25 mmol) and thioproline (**7**) (50 mg, 0.37 mmol) in methanol (20 mL) were reacted to yield solid compound **8h**; Yield (139 mg, 96%); m.p: 141–142 ºC; ^1^H-NMR (400 MHz, DMSO_-_*d*_6_): δ(ppm) = 10.50 (s, 1H, NH), 7.91 (s, 1H, Ar–H), 7.88 (d, *J* = 8.6 Hz, 1H, Ar–H), 7.65 (d, *J* = 2.2 Hz, 1H, Ar–H), 7.55–7.41 (m, 5H, Ar–H), 7.35–7.30 (m, 2H, Ar–H), 7.15 (t, *J* = 7.7 Hz, 1H, Ar–H), 6.94 (t, *J* = 7.6 Hz, 1H, Ar–H), 6.63 (d, *J* = 8.2 Hz, 1H, Ar–H), 4.62 (d, *J* = 11.4 Hz, 1H, CHCO), 4.48 (dd, *J* = 11.5, 9.2 Hz, 1H, NCHCH), 4.07–4.00 (m, 1H, NCH), 3.74 (d, *J* = 10.4 Hz, 1H, NCH_2(a)_), 3.38 (d, *J* = 10.4 Hz, 1H, NCH_2(b)_), 3.08–2.97 (m, 2H, SCH_2_), 1.80 (s, 3H, CH_3_); ^13^C-NMR (176 MHz, DMSO_-_*d*_6_): δ(ppm) = 190.06 (CO), 178.36 (CO), 142.55, 142.33, 141.03, 137.96, 136.02, 134.78, 132.24, 130.48, 129.88, 129.35, 129.04, 128.84, 128.01, 127.90, 125.23, 122.92, 120.96, 120.04, 109.47, 74.80, 73.87, 63.46, 54.01, 44.90, 35.47, 11.03 (CH_3_); IR (KBr, cm^–1^) ν_max_ = 3243, 3059, 2923, 2850, 1734, 1668, 1617, 1597, 1539, 1503, 1470, 1398, 1378, 1329, 1281, 1231, 1179, 1104, 1070, 1046, 979, 934, 863, 807, 764, 752, 694, 658, 603, 561, 488; [Anal. Calcd. for C_30_H_24_Cl_2_N_4_O_2_S: C, 62.61; H, 4.20; N, 9.74; Found: C, 62.53; H, 4.15; N, 9.59]; LC/MS (ESI, *m/z*): found 575.2 [M(_35_Cl) + H]^+^, 577.2 [M(_37_Cl) + H]^+^_;_ exact mass 574.10 for C_30_H_24_Cl_2_N_4_O_2_S.

#### 7'-(4-Fluorophenyl)-6'-(5-methyl-1-phenyl-1*H*-pyrazole-4-carbonyl)-1',6',7',7a'-tetrahydro-3'*H*-spiro[indoline-3,5'-pyrrolo[1,2-c]thiazol]-2-one (8i)


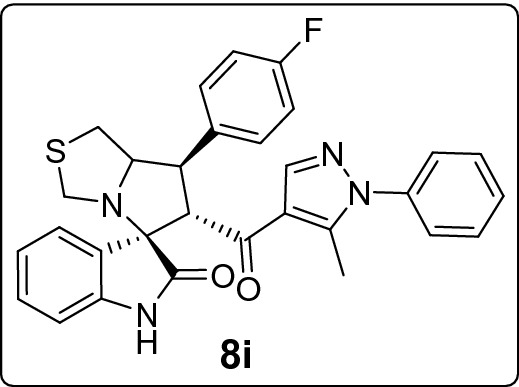
Following the general procedure (**GP1**), chalcone **5i** (77 mg, 0.25 mmol), isatin (**6)** (47 mg, 0.25 mmol) and thioproline (**7**) (50 mg, 0.37 mmol) in methanol (20 mL) were reacted to yield solid compound **8i**; Yield (106 mg, 81%); m.p: 136–137 ºC; ^1^H-NMR (400 MHz, DMSO_-_*d*_6_): δ(ppm) = 10.46 (s, 1H, NH), 7.88 (s, 1H, Ar–H), 7.57 (dd, *J* = 8.7, 5.5 Hz, 2H, Ar–H), 7.54–7.43 (m, 4H, Ar–H), 7.33 (d, *J* = 7.9 Hz, 2H, Ar–H), 7.21–7.12 (m, 3H, Ar–H), 6.92 (t, *J* = 7.5 Hz, 1H, Ar–H), 6.62 (d, *J* = 7.7 Hz, 1H, Ar–H), 4.45 (d, *J* = 11.7 Hz, 1H, CHCO), 4.13–4.05 (m, 1H, NCH), 3.87 (dd, *J* = 11.7, 9.4 Hz, 1H, NCHCH), 3.73 (d, *J* = 10.1 Hz, 1H, NCH_2(a)_), 3.37 (d, *J* = 10.1 Hz, 1H, NCH_2(b)_), 3.07–2.96 (m, 2H, SCH_2_), 1.81 (s, 3H, CH_3_); ^13^C-NMR (176 MHz, DMSO_-_*d*_6_): δ(ppm) = 190.29 (CO), 178.64 (CO), 162.05 & 160.67 (C_1_-F, *J*_C-F_ = 243.06 Hz), 142.61, 142.22, 140.95, 138.00, 135.64 & 135.62 (C_4_-F, *J*_C-F_ = 2.99 Hz), 130.13, 130.08, 129.79 & 129.39 (C_3_-F, *J*_C-F_ = 8.10 Hz), 128.88, 128.21, 125.27, 123.27, 120.90, 120.28, 115.57 & 115.45 (C_2_-F, *J*_C-F_ = 21.12 Hz), 109.41, 74.51, 73.82, 64.26, 53.61, 49.50, 35.80, 11.07 (CH_3_); IR (KBr, cm^–1^) ν_max_ = 3248, 3061, 2925, 1729, 1666, 1617, 1598, 1538, 1510, 1470, 1394, 1329, 1225, 1159, 1117, 1071, 1012, 934, 835, 807, 752, 694, 659, 606, 537; [Anal. Calcd. for C_30_H_25_FN_4_O_2_S: C, 68.68; H, 4.80; N, 10.68; Found: C, 68.54; H, 4.91; N, 10.63]; LC/MS (ESI, *m/z*): found 525.3 [M + H]^+^_;_ exact mass 524.17 for C_30_H_25_FN_4_O_2_S.

#### 7'-(3-Fluorophenyl)-6'-(5-methyl-1-phenyl-1*H*-pyrazole-4-carbonyl)-1',6',7',7a'-tetrahydro-3'*H*-spiro[indoline-3,5'-pyrrolo[1,2-c]thiazol]-2-one (8i)


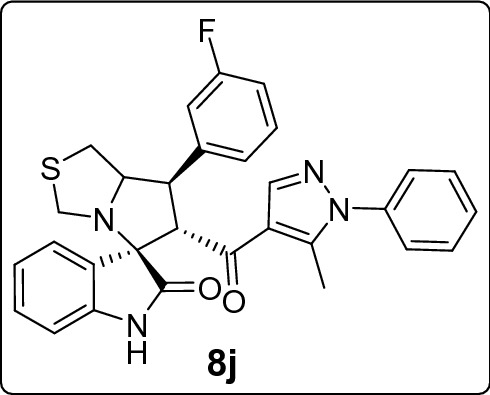
Following the general procedure (**GP1**), chalcone **5j** (77 mg, 0.25 mmol), isatin (**6)** (47 mg, 0.25 mmol) and thioproline (**7**) (50 mg, 0.37 mmol) in methanol (20 mL) were reacted to yield solid compound **8j**; Yield (126 mg, 96%); m.p: 125–126 ºC; ^1^H-NMR (400 MHz, DMSO_-_*d*_6_): δ(ppm) = 10.47 (s, 1H, NH), 7.92 (s, 1H, Ar–H), 7.54–7.49 (m, 2H, Ar–H), 7.49–7.43 (m, 2H, Ar–H), 7.42 (t, *J* = 2.2 Hz, 1H, Ar–H), 7.39–7.38 (m, 2H, Ar–H), 7.34–7.33 (m, 1H, Ar–H), 7.33–7.32 (m, 1H, Ar–H), 7.14 (t, *J* = 7.7 Hz, 1H, Ar–H), 7.11–7.05 (m, 1H, Ar–H), 6.92 (t, *J* = 7.6 Hz, 1H, Ar–H), 6.61 (d, *J* = 7.7 Hz, 1H, Ar–H), 4.50 (d, *J* = 11.6 Hz, 1H, CHCO), 4.12 (dt, *J* = 9.5, 4.7 Hz, 1H, NCH), 3.89 (dd, *J* = 11.7, 9.4 Hz, 1H, NCHCH), 3.72 (d, *J* = 10.1 Hz, 1H, NCH_2(a)_), 3.36 (d, *J* = 10.1 Hz, 1H, NCH_2(b)_), 3.04 (d, *J* = 4.7 Hz, 2H, SCH_2_), 1.80 (s, 3H, CH_3_); ^13^C-NMR (176 MHz, DMSO_-_*d*_6_): δ(ppm) = 190.24 (CO), 178.46 (CO), 163.05 & 161.66 (C_1_-F, *J*_C-F_ = 243.58 Hz), 142.55, 142.49 & 142.45 (C_3_-F, *J*_C-F_ = 7.22 Hz), 142.22, 141.04, 137.98, 130.64 & 130.59 (C_5_-F, *J*_C-F_ = 8.27 Hz), 129.75, 129.33, 128.81, 128.15, 125.23, 124.30 & 124.28 (C_4_-F, *J*_C-F_ = 2.46 Hz), 123.19, 120.83, 120.18, 115.12 & 115.00 (C_6_-F, *J*_C-F_ = 21.29 Hz), 114.01 & 113.89 (C_2_-F, *J*_C-F_ = 20.94 Hz), 109.34, 74.29, 73.77, 64.00, 53.49, 49.79, 35.73, 11.01 (CH_3_); IR (KBr, cm^–1^) ν_max_ = 3245, 3059, 2911, 2837, 1734, 1667, 1615, 1589, 1537, 1503, 1470, 1453, 1395, 1328, 1255, 1231, 1179, 1148, 1116, 1073, 981, 934, 809, 752, 693, 659, 599, 520; [Anal. Calcd. for C_30_H_25_FN_4_O_2_S: C, 68.68; H, 4.80; N, 10.68; Found: C, 68.59; H, 4.87; N, 10.57]; LC/MS (ESI, *m/z*): found 525.3 [M + H]^+^_;_ exact mass 524.17 for C_30_H_25_FN_4_O_2_S.

#### 6'-(5-Methyl-1-phenyl-1*H*-pyrazole-4-carbonyl)-7'-(4-(trifluoromethyl)phenyl)-1',6',7',7a'-tetrahydro-3'*H*-spiro[indoline-3,5'-pyrrolo[1,2-c]thiazol]-2-one (8k)


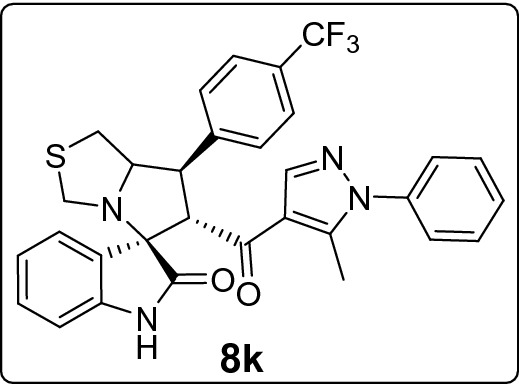
Following the general procedure (**GP1**), chalcone **5k** (89 mg, 0.25 mmol), isatin (**6)** (47 mg, 0.25 mmol) and thioproline (**7**) (50 mg, 0.37 mmol) in methanol (20 mL) were reacted to yield solid compound **8k**; Yield (140 mg, 98%); m.p: 138–139 ºC; ^1^H-NMR (400 MHz, DMSO_-_*d*_6_): δ(ppm) = 10.49 (s, 1H, NH), 7.89 (s, 1H, Ar–H), 7.79 (d, *J* = 8.2 Hz, 2H, Ar–H), 7.72 (d, *J* = 8.2 Hz, 2H, Ar–H), 7.54–7.49 (m, 2H, Ar–H), 7.49–7.44 (m, 2H, Ar–H), 7.35–7.30 (m, 2H, Ar–H), 7.15 (t, *J* = 7.7 Hz, 1H, Ar–H), 6.93 (t, *J* = 7.6 Hz, 1H, Ar–H), 6.62 (d, *J* = 7.8 Hz, 1H, Ar–H), 4.54 (d, *J* = 11.6 Hz, 1H, CHCO), 4.15 (dt, *J* = 9.4, 4.7 Hz, 1H, NCH), 3.97 (dd, *J* = 11.7, 9.4 Hz, 1H, NCHCH), 3.74 (d, *J* = 10.1 Hz, 1H, NCH_2(a)_), 3.38 (d, *J* = 10.1 Hz, 1H, NCH_2(b)_), 3.04 (d, *J* = 4.8 Hz, 2H, SCH_2_), 1.80 (s, 3H CH_3_); ^13^C-NMR (176 MHz, DMSO_-_*d*_6_): δ(ppm) = 190.16 (CO), 178.49 (CO), 144.39, 142.63, 142.25, 140.99, 137.97, 129.85, 129.37, 129.23, 128.87, 128.14, [125.62, 125.60, 125.58, 125.56 (CF_3_)], 125.25, 125.10, 123.55, 123.14, 120.92, 120.11, 109.43, 74.31, 73.84, 64.12, 53.57, 49.95, 35.73, 11.03 (CH_3_); IR (KBr, cm^–1^) ν_max_ = 3244, 2925, 2853, 1734, 1668, 1617, 1598, 1538, 1503, 1470, 1393, 1287, 1231, 1165, 1068, 1017, 934, 808, 752, 723, 694, 672, 604, 531; [Anal. Calcd. for C_31_H_25_F_3_N_4_O_2_S: C, 64.80; H, 4.39; N, 9.75; Found: C, 64.86; H, 4.45; N, 9.68]; LC/MS (ESI, *m/z*): found 575.3 [M + H]^+^_;_ exact mass 574.17 for C_31_H_25_F_3_N_4_O_2_S.

#### 6'-(5-Methyl-1-phenyl-1*H*-pyrazole-4-carbonyl)-7'-(pyridin-2-yl)-3',6',7',7a'-tetrahydro-1'*H*-spiro[indoline-3,5'-pyrrolo[1,2-c]thiazol]-2-one (8l)


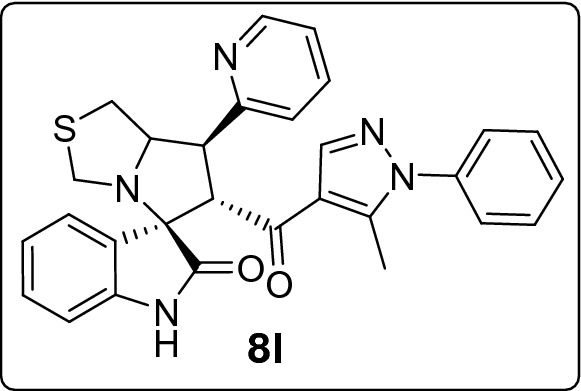
Following the general procedure (**GP1**), chalcone **5l** (73 mg, 0.25 mmol), isatin (**6)** (47 mg, 0.25 mmol) and thioproline (**7**) (50 mg, 0.37 mmol) in methanol (20 mL) were reacted to yield solid compound **8l**; Yield (123 mg, 96%); m.p: 135–136 ºC; ^1^H-NMR (400 MHz, DMSO_-_*d*_6_): δ(ppm) = 10.45 (s, 1H, NH), 8.59 (ddd, *J* = 4.8, 1.9, 0.9 Hz, 1H, Ar–H), 7.80 (s, 1H, Ar–H), 7.76 (td, *J* = 7.7, 1.8 Hz, 1H, Ar–H), 7.53–7.43 (m, 5H, Ar–H), 7.37–7.31 (m, 2H, Ar–H), 7.27 (ddd, *J* = 7.5, 4.8, 1.1 Hz, 1H, Ar–H), 7.14 (td, *J* = 7.7, 1.3 Hz, 1H, Ar–H), 6.92 (td, *J* = 7.6, 1.1 Hz, 1H, Ar–H), 6.65–6.59 (dd, *J* = 7.6, 1.2 Hz, 1H, Ar–H), 4.83 (d, *J* = 11.6 Hz, 1H, CHCO), 4.19 (ddd, *J* = 9.2, 6.5, 2.6 Hz, 1H, NCH), 4.02 (dd, *J* = 11.5, 9.3 Hz, 1H, NCHCH), 3.72 (d, *J* = 10.3 Hz, 1H, NCH_2(a)_), 3.34 (d, *J* = 10.3 Hz, 1H, NCH_2(b)_), 3.16 (dd, *J* = 11.5, 2.6 Hz, 1H, SCH_2(a)_), 3.05 (dd, *J* = 11.5, 6.5 Hz, 1H, SCH_2(b)_), 1.79 (s, 3H, CH_3_); ^13^C-NMR (176 MHz, DMSO_-_*d*_6_): δ(ppm) = 190.78 (CO), 178.83 (CO), 158.28, 149.52, 142.60, 142.27, 140.74, 138.02, 136.98, 129.79, 129.40, 128.88, 128.19, 125.28, 124.18, 123.37, 122.53, 120.87, 120.13, 109.40, 73.83, 73.41, 62.65, 53.93, 51.79, 36.14, 11.05 (CH_3_); IR (KBr, cm^–1^) ν_max_ = 3249, 3058, 3012, 2924, 2841, 1733, 1664, 1617, 1591, 1569, 1537, 1502, 1470, 1438, 1395, 1328, 1285, 1268, 1222, 1181, 1116, 1072, 995, 980, 934, 877, 858, 811, 761, 751, 723, 695, 681, 668, 659, 599, 538; [Anal. Calcd. for C_29_H_25_N_5_O_2_S: C, 68.62; H, 4.96; N, 13.80; Found: C, 68.49; H, 5.07; N, 13.87]; LC/MS (ESI, *m/z*): found 508.2 [M + H]^+^_;_ exact mass 507.17 for C_29_H_25_N_5_O_2_S.

#### 7'-(3-Bromophenyl)-6'-(5-methyl-1-phenyl-1*H*-pyrazole-4-carbonyl)-3',6',7',7a'-tetrahydro-1'*H*-spiro[indoline-3,5'-pyrrolo[1,2-c]thiazol]-2-one (8m)


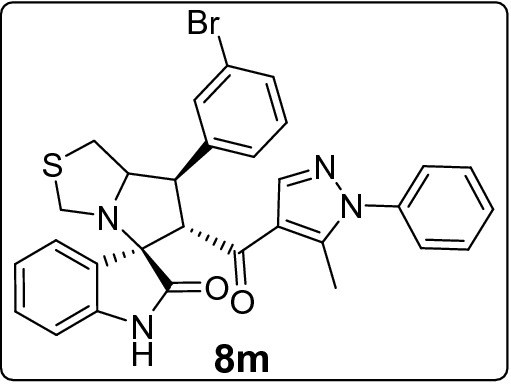
Following the general procedure (**GP1**), chalcone **5m** (92 mg, 0.25 mmol), isatin (**6)** (47 mg, 0.25 mmol) and thioproline (**7**) (50 mg, 0.37 mmol) in methanol (20 mL) were reacted to yield solid compound **8m**; Yield (143 mg, 98%); m.p: 132–133 ºC; ^1^H-NMR (400 MHz, DMSO_-_*d*_6_): δ(ppm) = 10.47 (s, 1H, NH), 7.91 (s, 1H, Ar–H), 7.75 (t, *J* = 1.8 Hz, 1H, Ar–H), 7.57 (d, *J* = 7.7 Hz, 1H, Ar–H), 7.54–7.49 (m, 2H, Ar–H), 7.49–7.42 (m, 3H, Ar–H), 7.36–7.30 (m, 3H, Ar–H), 7.14 (t, *J* = 7.7 Hz, 1H, Ar–H), 6.92 (t, *J* = 7.6 Hz, 1H, Ar–H), 6.61 (d, *J* = 7.8 Hz, 1H, Ar–H), 4.49 (d, *J* = 11.7 Hz, 1H, CHCO), 4.12 (dt, *J* = 9.4, 4.7 Hz, 1H, NCH), 3.87 (dd, *J* = 11.7, 9.4 Hz, 1H, NCHCH), 3.72 (d, *J* = 10.1 Hz, 1H, NCH_2(a)_), 3.36 (d, *J* = 10.1 Hz, 1H, NCH_2(b)_), 3.08–2.98 (m, 2H, SCH_2_), 1.80 (s, 3H, CH_3_); ^13^C-NMR (176 MHz, DMSO_-_*d*_6_): δ(ppm) = 190.25 (CO), 178.45 (CO), 142.57, 142.39, 142.22, 141.04, 137.98, 131.15, 130.92, 130.09, 129.77, 129.34, 128.83, 128.14, 127.24, 125.24, 123.18, 122.00, 120.85, 120.15, 109.35, 74.29, 73.78, 64.13, 53.49, 49.73, 35.73, 11.02 (CH_3_); IR (KBr, cm^–1^) ν_max_ = 3245, 3056, 2911, 2838, 1732, 1667, 1617, 1595, 1536, 1502, 1470, 1393, 1328, 1223, 1179, 1116, 1073, 934, 809, 752, 725, 693, 659, 598; [Anal. Calcd. for C_30_H_25_BrN_4_O_2_S: C, 61.54; H, 4.30; N, 9.57; Found: C, 61.67; H, 4.41; N, 9.55]; LC/MS (ESI, *m/z*): found 585.2 [M(_79_Br) + H]^+^, 587.2 [M(_81_Br) + H]^+^_;_ exact mass 584.09 for C_30_H_25_BrN_4_O_2_S.

#### 6'-(5-Methyl-1-phenyl-1*H*-pyrazole-4-carbonyl)-7'-(4-nitrophenyl)-3',6',7',7a'-tetrahydro-1'*H*-spiro[indoline-3,5'-pyrrolo[1,2-c]thiazol]-2-one (8n)


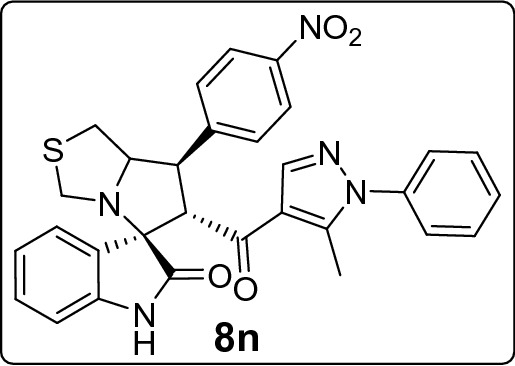
Following the general procedure (**GP1**), chalcone **5n** (83 mg, 0.25 mmol), isatin (**6)** (47 mg, 0.25 mmol) and thioproline (**7**) (50 mg, 0.37 mmol) in methanol (20 mL) were reacted to yield solid compound **8n**; Yield (135 mg, 98%); m.p: 142–143 ºC; ^1^H-NMR (400 MHz, DMSO_-_*d*_6_): δ(ppm) = 10.50 (s, 1H, NH), 8.21 (d, *J* = 8.7 Hz, 2H, Ar–H), 7.91–7.84 (m, 3H, Ar–H), 7.54–7.49 (m, 2H, Ar–H), 7.49–7.44 (m, 2H, Ar–H), 7.35–7.30 (m, 2H, Ar–H), 7.15 (t, *J* = 7.7 Hz, 1H, Ar–H), 6.93 (t, *J* = 7.6 Hz, 1H, Ar–H), 6.62 (d, *J* = 7.6 Hz, 1H, Ar–H), 4.56 (d, *J* = 11.6 Hz, 1H, CHCO), 4.20–4.12 (m, 1H, NCH), 4.08–4.01 (d, *J* = 11.6, 9.6 Hz, 1H, NCHCH), 3.74 (d, *J* = 10.1 Hz, 1H,NCH_2(a)_), 3.38 (d, *J* = 10.1 Hz, 1H NCH_2(b)_), 3.05 (d, *J* = 4.7 Hz, 2H, SCH_2_), 1.79 (s, 3H, CH_3_); ^13^C-NMR (176 MHz, DMSO_-_*d*_6_): δ(ppm) = 190.08 (CO), 178.40 (CO), 147.51, 146.73, 142.64, 142.27, 141.02, 137.96, 129.90, 129.75, 129.38, 128.88, 128.12, 125.25, 123.81, 123.07, 120.94, 120.04, 109.45, 74.18, 73.86, 64.21, 53.53, 49.92, 35.69, 11.01 (CH_3_); IR (KBr, cm^–1^) ν_max_ = 3247, 3076, 2920, 2839, 1733, 1666, 1617, 1597, 1533, 1520, 1502, 1470, 1394, 1347, 1284, 1231, 1180, 1110, 1071, 1012, 934, 839, 808, 763, 750, 724, 695, 682, 658, 536; [Anal. Calcd. for C_30_H_25_N_5_O_4_S: C, 65.32; H, 4.57; N, 12.70; Found: C, 65.23; H, 4.52; N, 12.63]; LC/MS (ESI, *m/z*): found 552.2 [M + H]^+^_;_ exact mass 551.16 for C_30_H_25_N_5_O_4_S.

#### 6'-(5-Methyl-1-phenyl-1*H*-pyrazole-4-carbonyl)-7'-(3-nitrophenyl)-3',6',7',7a'-tetrahydro-1'*H*-spiro[indoline-3,5'-pyrrolo[1,2-c]thiazol]-2-one (8o)


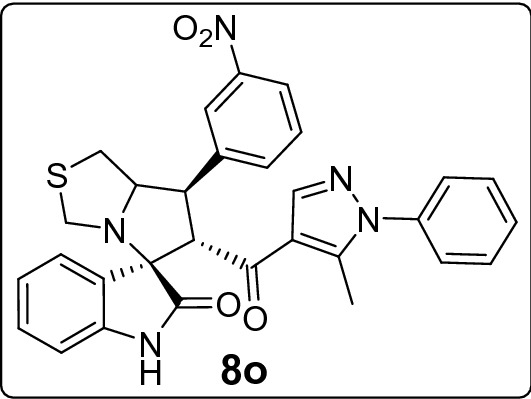
Following the general procedure (**GP1**), chalcone **5o** (83 mg, 0.25 mmol), isatin (**6)** (47 mg, 0.25 mmol) and thioproline (**7**) (50 mg, 0.37 mmol) in methanol (20 mL) were reacted to yield solid compound **8o**; Yield (132 mg, 96%); m.p: 146–147 ºC; ^1^H-NMR (400 MHz, DMSO_-_*d*_6_): δ(ppm) = 10.49 (s, 1H, NH), 8.38 (t, *J* = 2.0 Hz, 1H, Ar–H), 8.13 (ddd, *J* = 8.2, 2.3, 1.0 Hz, 1H, Ar–H), 8.06 (d, *J* = 7.7 Hz, 1H, Ar–H), 7.90 (s, 1H, Ar–H), 7.67 (t, *J* = 8.0 Hz, 1H, Ar–H), 7.55–7.46 (m, 4H, Ar–H), 7.34–7.29 (m, 2H, Ar–H), 7.18–7.11 (m, 1H, Ar–H), 6.93 (t, *J* = 7.6 Hz, 1H, Ar–H), 6.61 (d, *J* = 7.9 Hz, 1H, Ar–H), 4.55 (d, *J* = 11.6 Hz, 1H, CHCO), 4.20 (ddd, *J* = 9.5, 6.0, 3.6 Hz, 1H, NCH), 4.05 (dd, *J* = 11.5, 9.4 Hz, 1H, NCHCH), 3.74 (d, *J* = 10.1 Hz, 1H, NCH_2(a)_), 3.38 (d, *J* = 10.1 Hz, 1H, NCH_2(b)_), 3.10–2.98 (m, 2H, SCH_2_), 1.78 (s, 3H, CH_3_); ^13^C-NMR (176 MHz, DMSO_-_*d*_6_): δ(ppm) = 190.32 (CO), 178.49 (CO), 148.13, 142.63, 142.30, 141.84, 141.12, 138.01, 135.19, 130.33, 129.92, 129.42, 128.92, 128.20, 125.29, 123.42, 123.16, 122.29, 120.99, 120.10, 109.46, 74.21, 73.91, 64.47, 53.57, 49.68, 35.75, 11.01 (CH_3_); IR (KBr, cm^–1^) ν_max_ = 3215, 3081, 2912, 1733, 1666, 1617, 1597, 1529, 1502, 1470, 1395, 1349, 1283, 1229, 1179, 1116, 1074, 936, 832, 806, 753, 724, 695, 683, 659, 598; [Anal. Calcd. for C_30_H_25_N_5_O_4_S: C, 65.32; H, 4.57; N, 12.70; Found: C, 65.39; H, 4.71; N, 12.59]; LC/MS (ESI, *m/z*): found 552.2 [M + H]^+^_;_ exact mass 551.16 for C_30_H_25_N_5_O_4_S.

#### 6'-(5-Methyl-1-phenyl-1*H*-pyrazole-4-carbonyl)-7'-(p-tolyl)-1',6',7',7a'-tetrahydro-3'*H*-spiro[indoline-3,5'-pyrrolo[1,2-c]thiazol]-2-one (8p)


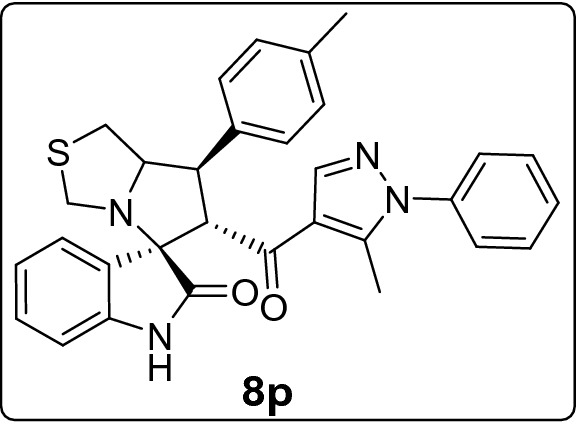
Following the general procedure (**GP1**), chalcone **5p** (76 mg, 0.25 mmol), isatin (**6)** (47 mg, 0.25 mmol) and thioproline (**7**) (50 mg, 0.37 mmol) in methanol (20 mL) were reacted to yield solid compound **8p**; Yield (127 mg, 98%); m.p: 134–135 ºC; ^1^H-NMR (700 MHz, DMSO_-_*d*_6_): δ(ppm) = 10.47 (s, 1H, NH), 7.86 (s, 1H, Ar–H), 7.53–7.49 (m, 2H, Ar–H), 7.48–7.43 (m, 2H, Ar–H), 7.39 (d, *J* = 6.3 Hz, 2H, Ar–H), 7.33 (d, *J* = 7.6 Hz, 2H, Ar–H), 7.15 (dd, *J* = 8.2, 7.2 Hz, 3H, Ar–H), 6.92 (t, *J* = 7.6 Hz, 1H, Ar–H), 6.62 (d, *J* = 7.8 Hz, 1H, Ar–H), 4.45 (d, *J* = 11.8 Hz, 1H, CHCO), 4.0–4.09 (m, 1H, NCH), 3.80 (dd, *J* = 11.8, 9.4 Hz, 1H, NCHCH), 3.72 (d, *J* = 10.2 Hz, 1H, NCH_2(a)_), 3.35 (d, *J* = 10.2 Hz, 1H, NCH_2(b)_), 3.03–2.96 (m, 2H, SCH_2_), 2.26 (s, 3H, CH_3_), 1.82 (s, 3H, CH_3_); ^13^C-NMR (176 MHz, DMSO_-_*d*_6_): δ(ppm) = 190.25 (CO), 178.65 (CO), 142.54, 142.17, 140.84, 137.97, 136.40, 136.22, 129.68, 129.33, 128.81, 128.21, 127.95, 125.22, 123.31, 120.83, 120.33, 109.34, 74.61, 73.79, 64.09, 53.59, 49.99, 35.86, 20.64 (CH_3_), 11.08 (CH_3_); IR (KBr, cm^–1^) ν_max_ = 3249, 3054, 3020, 2919, 1733, 1666, 1617, 1597, 1537, 1503, 1471, 1394, 1328, 1382, 1227, 1179, 1115, 1071, 933, 805, 753, 727, 695, 659, 606, 534; [Anal. Calcd. for C_31_H_28_N_4_O_2_S: C, 71.51; H, 5.42; N, 10.76; Found: C, 71.62; H, 5.37; N, 10.65]; LC/MS (ESI, *m/z*): found 521.3 [M + H]^+^, exact mass 520.19 for C_31_H_28_N_4_O_2_S.

### Anticancer evaluation

#### Determination of antiproliferative activity by the MTT (3-(4,5-dimethylthiazol-2-yl)-2,5-diphenyltetrazolium bromide) assay

Human tumor cell lines A2780 [ovary, adenocarcinoma, accession number HTL98008, obtained from Interlab Cell Line Collection (ICLC), Genova, Italy], A549 (lung, carcinoma, HTL03001, ICLC), MDA-MB-453 (breast, carcinoma, HTL01013, ICLC), and HepG2 (liver, carcinoma, HB-8065, American Type Culture Collection, Rockville, MA, USA ) were plated at 1.4 × 10^4^/ml, 0.65 × 10^4^/mL, 4.7 × 10^4^/mL, and 1.0 × 10^4^/mL, respectively, in 180 μL complete medium (RPMI 1640 for A2780 and A549 and DMEM for MDA-MB-453 and HepG2) into flat-bottomed 96-well microtiter plates. After 6–8 h cells were treated with 20 μL containing five 1:10 fold concentrations of our compounds diluted in FCS containing 3% DMSO. As normal non-cancer cell targets we used peripheral blood lymphocytes obtained from three healthy volunteers, isolated by gradient centrifugation (Lympholyte-H Cell Separation Media, EuroClone, Pero, MI, Italy) and stimulated with 1% phytohemagglutinin and 100 U/ml recombinant IL-2. One week after stimulation cells were ready for use at 15,000/well.

In order to evaluate the effect of our compounds on the antiproliferative activity of doxorubicin (a strong inducer of apoptosis) in a second series of experiments, A549 cells were treated with 0.2 μM compounds for 6 h and then with doxorubicin (1:5) fold concentrations starting from 1 μM for 72 h.

In both cases, after 72 h treatment, the MTT assay was applied as described elsewhere ^62^. The IC_50_ values were calculated from the analysis of single concentration–response curves, each final value is the mean of 5–6 experiments. IC_100_ and IC_75_ were extrapolated from the mean concentration–response curves.

#### Determination of apoptosis by Annexin-V assay

The induction of apoptosis was evaluated in A549 cells characterized by wild-type p53 and MDM2 proteins. They were plated at the opportune concentration (see above) into 25 cm^2^ flasks in 10 mL complete medium. After 6–8 h the compounds were added at their specific IC_75_s, as previously calculated by the MTT assay.

All floating and adherent cells were harvested three days later, washed with normal saline buffer, and apoptosis determined by double staining with Annexin-V-FITC and propidium iodide (PI) (rh AnnexinV/FITC Kit, Bender MedSystem GmbH, Vienna, Austria). Briefly, 0.5 × 10^6^ cells were resuspended in binding buffer 1× and stained with 5 μL of Annexin-V-FITC. After 10 min incubation 10 μL of PI were added and samples incubated for 5 min. Samples were immediately analyzed using a Flow Cytometer (FACScan, BD Biosciences, Milano, Italy) with dedicated software.

#### Western blot

A549 cells were treated with different concentrations of the molecules as indicated. Cell extracts were prepared as described^63^. Ten-20 μg of total proteins were resolved on 4–15% Mini protean TGX precast gels (Bio-Rad) and transferred to nitrocellulose membrane using 1704158 Trans-Blot Turbo Mini NC Transfer Packs (Bio-Rad) in a Trans-Blot Turbo apparatus (Bio-Rad). Membranes were blocked with 5% non-fat dry milk in 0.1% Tween-20 in PBS for 1 h, then incubated for 1 h at room temperature or overnight at 4 °C with the appropriate primary antibody. The following antibodies were employed: anti-p53 (DO-1, sc-126, Santa Cruz Biotechnology, 1:20.000), anti-p21 (2946, Cell Signaling, 1:2000), anti-MDM2 (SMP14, sc-965, Santa Cruz Biotechnology, 1:200), anti β-actin (AC-74, Sigma-Aldrich, 1:30.000); secondary anti-mouse IgG peroxidase conjugate (A9044, Sigma–Aldrich, 1:10.000) and secondary anti-rabbit peroxidase conjugate (A9169, Sigma-Aldrich, 1: 12.000). Detection was carried out with ECL FAST PICO (ECL-1002, Immunological Sciences, Roma, Italy). Chemiluminescence was analysed by Alliance LD, UVITEC Cambridge (Cambridge, UK).

### Docking

Docking simulations were performed employing MOE 2016.0802^61^. The studied compounds were built in silico and subjected to energy minimization using the MMFF94x force field at a gradient of 0.01 RMSD. Coordinates for MDM2 was downloaded from RCSB PDB (PDB ID: 5LAW^39^, handled with MOE 2016.0802). Unwanted amino acid chains, solvents, and ligands were eliminated. The preparation procedure was conducted employing “Structure Preparation” module with the default settings. The molecular docking simulations were conducted employing ‘Triangle Matcher’ as the placement method and ‘London dG’ scoring for calculating Gibbs energy for binding.

### Data analysis and statistics

Student ‘t’ test was used for the analysis of data.

## Conclusion

This study portrays pharmacophoric hybridization design, multicomponent synthesis, molecular modeling studies and biological evaluation of new series of pyrazole-spirooxindoles as direct MDM2 inhibitors. Results demonstrated that the general antiproliferative pattern of the studied 7’-substituted 6'-(pyrazole-4-carbonyl)tetrahydro-3'*H*-spiro[indoline-3,5'-pyrrolo[1,2-c]thiazol]-2-one echoed the parent spirooxindoline architecture’s intrinsic antitumor potential justifying the adopted design rational. The 7'-(2,4-dichlorophenyl) substitution **(8h)** conferred the highest anticancer potency to the scaffold against A2780 and HepG2 cells, whereas the 3-bromophenyl (**8m)** and the 4-(trifluoromethyl)phenyl **(8k)** moieties were the optimized substituents for efficient antiproliferative activities against A549 and MDA-MB-453 cells, respectively. On the other hand, installing pyridin-2-yl **(8l)** and furan-2yl **(8b)** on the spiro ring was detrimental to anticancer potencies against all the screened cell lines. Further experiments showed that **8h** and **8j** potentiated doxorubicin anticancer potential by more than 25% in combinations. Western blot analysis revealed a dose-dependent down regulation of MDM2 in A549 cells after treatment with that **8k** and **8m**. Docking simulations showed that the promising derivative** (8k** and **8m)** accommodated readily into the MDM2 active site posing various interactions with the key amino acid residue**s.**

## Supplementary Information

Supplementary Information.

## Data Availability

Data are available from the corresponding authors upon reasonable request.

## References

[1] W. H. Organization. *WHO Report on Cancer: Setting Priorities, Investing Wisely and Providing Care for All*. (2020).

[2] Reed JC (1999). Dysregulation of apoptosis in cancer. J. Clin. Oncol..

[3] Wong RS (2011). Apoptosis in cancer: From pathogenesis to treatment. J. Exp. Clin. Cancer Res..

[4] Pistritto G, Trisciuoglio D, Ceci C, Garufi A, D'Orazi G (2016). Apoptosis as anticancer mechanism: Function and dysfunction of its modulators and targeted therapeutic strategies. Aging (Albany N.Y.).

[5] Fridman JS, Lowe SW (2003). Control of apoptosis by p53. Oncogene.

[6] Vazquez A, Bond EE, Levine AJ, Bond GL (2008). The genetics of the p53 pathway, apoptosis and cancer therapy. Nat. Rev. Drug Discov..

[7] Khoo KH, Verma CS, Lane DP (2014). Drugging the p53 pathway: Understanding the route to clinical efficacy. Nat. Rev. Drug Discov..

[8] Villunger A (2003). p53-and drug-induced apoptotic responses mediated by BH3-only proteins puma and noxa. Science.

[9] Chen L (2005). Differential targeting of prosurvival Bcl-2 proteins by their BH3-only ligands allows complementary apoptotic function. Mol. Cell.

[10] Mihara M (2003). p53 has a direct apoptogenic role at the mitochondria. Mol. Cell.

[11] Chipuk JE (2004). Direct activation of Bax by p53 mediates mitochondrial membrane permeabilization and apoptosis. Science.

[12] Tovar C (2013). MDM2 small-molecule antagonist RG7112 activates p53 signaling and regresses human tumors in preclinical cancer models. Cancer Res..

[13] Lujambio A (2013). Non-cell-autonomous tumor suppression by p53. Cell.

[14] Schuler M, Bossy-Wetzel E, Goldstein JC, Fitzgerald P, Green DR (2000). p53 induces apoptosis by caspase activation through mitochondrial cytochrome c release. J. Biol. Chem..

[15] Oliner JD (1993). Oncoprotein MDM2 conceals the activation domain of tumour suppressor p53. Nature.

[16] Haupt Y, Maya R, Kazaz A, Oren M (1997). Mdm2 promotes the rapid degradation of p53. Nature.

[17] Kubbutat MH, Jones SN, Vousden KH (1997). Regulation of p53 stability by Mdm2. Nature.

[18] Midgley CA, Lane DP (1997). p53 protein stability in tumour cells is not determined by mutation but is dependent on Mdm2 binding. Oncogene.

[19] Ganguli G, Abecassis J, Wasylyk B (2000). MDM2 induces hyperplasia and premalignant lesions when expressed in the basal layer of the epidermis. EMBO J..

[20] Eymin B, Gazzeri S, Brambilla C, Brambilla E (2002). Mdm2 overexpression and p14ARF inactivation are two mutually exclusive events in primary human lung tumors. Oncogene.

[21] Polsky D (2001). HDM2 protein overexpression, but not gene amplification, is related to tumorigenesis of cutaneous melanoma. Cancer Res..

[22] Oliner J, Kinzler KW, Meltzer P, George DL, Vogelstein B (1992). Amplification of a gene encoding a p53-associated protein in human sarcomas. Nature.

[23] Anifowose A, Agbowuro AA, Yang X, Wang B (2020). Anticancer Strategies by Upregulating P53 through Inhibition of its Ubiquitination by MDM2. Med. Chem. Res..

[24] Beloglazkina A, Zyk N, Majouga A, Beloglazkina E (2020). Recent small-molecule inhibitors of the p53–MDM2 protein–protein interaction. Molecules.

[25] Riedinger C, McDonnell JM (2009). Inhibitors of MDM2 and MDMX: A structural perspective. Future Med. Chem..

[26] Millard M, Pathania D, Grande F, Xu S, Neamati N (2011). Small-molecule inhibitors of p53-MDM2 interaction: The 2006–2010 update. Curr. Pharm. Des..

[27] Vassilev LT (2007). MDM2 inhibitors for cancer therapy. Trends Mol. Med..

[28] Khoury K, Popowicz GM, Holak TA, Dömling A (2011). The p53-MDM2/MDMX axis–A chemotype perspective. MedChemComm.

[29] Wang S, Zhao Y, Aguilar A, Bernard D, Yang C-Y (2017). Targeting the MDM2–p53 protein–protein interaction for new cancer therapy: Progress and challenges. Cold Spring Harb. Perspect. Med..

[30] Lane DP, Cheok CF, Lain S (2010). p53-based cancer therapy. Cold Spring Harb. Perspect. Biol..

[31] Popowicz GM, Dömling A, Holak TA (2011). The structure-based design of MDM2/MDMX–p53 inhibitors gets serious. Angew. Chem. Int. Ed..

[32] Dickens, M. P., Fitzgerald, R. & Fischer, P. M. *Seminar on Cancer Biology*. 10–18 (Elsevier). 10.1016/j.semcancer.2009.10.003 19897042

[33] Ding K (2005). Structure-based design of potent non-peptide MDM2 inhibitors. J. Am. Chem. Soc..

[34] Ding K (2006). Structure-based design of spiro-oxindoles as potent, specific small-molecule inhibitors of the MDM2−p53 interaction. J. Med. Chem..

[35] Wang S (2014). SAR405838: An optimized inhibitor of MDM2–p53 interaction that induces complete and durable tumor regression. Cancer Res..

[36] Zhang Z (2014). Discovery of potent and orally active p53-MDM2 inhibitors RO5353 and RO2468 for potential clinical development. ACS Med. Chem. Lett..

[37] Nakamaru, K., Seki, T., Tazaki, K. & Tse, A. (AACR, 2015).

[38] Popowicz GM (2010). Structures of low molecular weight inhibitors bound to MDMX and MDM2 reveal new approaches for p53-MDMX/MDM2 antagonist drug discovery. Cell Cycle.

[39] Gollner A (2016). Discovery of novel spiro [3 H-indole-3, 2′-pyrrolidin]-2 (1 H)-one compounds as chemically stable and orally active inhibitors of the MDM2–p53 interaction. J. Med. Chem..

[40] Chapeau EA (2017). Resistance mechanisms to TP53-MDM2 inhibition identified by in vivo piggyBac transposon mutagenesis screen in an Arf−/− mouse model. Proc. Natl. Acad. Sci..

[41] Vogelstein B (2013). Cancer genome landscapes. Science.

[42] Long J (2010). Multiple distinct molecular mechanisms influence sensitivity and resistance to MDM2 inhibitors in adult acute myelogenous leukemia. Blood J. Am. Soc. Hematol..

[43] París R, Henry RE, Stephens SJ, McBryde M, Espinosa JM (2008). Multiple p53-independent gene silencing mechanisms define the cellular response to p53 activation. Cell Cycle.

[44] Aziz YMA (2021). Design, synthesis, chemical and biochemical insights into novel hybrid spirooxindole-based p53-MDM2 inhibitors with potential Bcl2 signaling attenuation. Front. Chem..

[45] Lotfy G (2021). Molecular hybridization design and synthesis of novel spirooxindole-based MDM2 inhibitors endowed with BCL2 signaling attenuation; A step towards the next generation p53 activators. Bioorg. Chem..

[46] Al-Majid AM (2021). Stereoselective synthesis of the di-spirooxindole analogs based oxindole and cyclohexanone moieties as potential anticancer agents. Molecules.

[47] Xie X (2021). Design and organocatalytic synthesis of spirooxindole–cyclopentene–isoxazole hybrids as novel MDM2–p53 inhibitors. Org. Chem. Front..

[48] Zhou L-M, Qu R-Y, Yang G-F (2020). An overview of spirooxindole as a promising scaffold for novel drug discovery. Expert Opin. Drug Discov..

[49] Islam MS (2020). Synthesis, anticancer activity, and molecular modeling of new halogenated spiro [pyrrolidine-thiazolo-oxindoles] derivatives. Appl. Sci..

[50] Barakat A (2019). Design and synthesis of new substituted spirooxindoles as potential inhibitors of the MDM2–p53 interaction. Bioorg. Chem..

[51] Islam MS (2019). Synthesis of new thiazolo-pyrrolidine–(spirooxindole) tethered to 3-acylindole as anticancer agents. Bioorg. Chem..

[52] Yu, B. & Liu, H.-M. *Targeting Protein–Protein Interactions by Small Molecules*. 213–237 (Springer, 2018).

[53] Aguilar, A. *et al.* Discovery of 4-((3′ R, 4′ S, 5′ R)-6 ″-chloro-4′-(3-chloro-2-fluorophenyl)-1′-ethyl-2 ″-oxodispiro [cyclohexane-1, 2′-pyrrolidine-3′, 3 ″-indoline]-5′-carboxamido) bicyclo [2.2. 2] octane-1-carboxylic acid (AA-115/APG-115): A potent and orally active murine double minute 2 (MDM2) inhibitor in clinical development. *J. Med. Chem.* **60**, 2819–2839 (2017). 10.1021/acs.jmedchem.6b01665 PMC5394527 28339198

[54] Yu, B., Zheng, Y.-C., Shi, X.-J., Qi, P.-P. & Liu, H.-M. Natural product-derived spirooxindole fragments serve as privileged substructures for discovery of new anticancer agents. *Anti-Cancer Agents Med. Chem. (Formerly Curr. Med. Chem.-Anti-Cancer Agents)* **16**, 1315–1324 (2016). 10.2174/1871520615666151102093825 26522954

[55] Gomha SM, Farghaly TA, Sayed AR, Abdalla MM (2016). Synthesis of pyrazolyl-pyrazoles and pyrazolyl-[1, 2, 4]-triazolo [3, 4-d][1, 5] benzothiazepines as p53 activators using hydrazonoyl chlorides. J. Heterocycl. Chem..

[56] Furet P (2016). Discovery of a novel class of highly potent inhibitors of the p53–MDM2 interaction by structure-based design starting from a conformational argument. Bioorg. Med. Chem. Lett..

[57] Christner SM (2015). In vitro cytotoxicity and in vivo efficacy, pharmacokinetics, and metabolism of pyrazole-based small molecule inhibitors of MDM2/4–p53 interaction. Cancer Chemother. Pharmacol..

[58] Hu C, Gao Y, Du W (2016). Design, synthesis, and biological evaluation of pyrazole derivatives. Chem. Biol. Drug Des..

[59] Hyun S-Y, Jang Y-J (2015). p53 activates G1 checkpoint following DNA damage by doxorubicin during transient mitotic arrest. Oncotarget.

[60] Lüpertz R, Wätjen W, Kahl R, Chovolou Y (2010). Dose-and time-dependent effects of doxorubicin on cytotoxicity, cell cycle and apoptotic cell death in human colon cancer cells. Toxicology.

[61] Heck RF (1968). Acylation, methylation, and carboxyalkylation of olefins by Group VIII metal derivatives. J. Am. Chem. Soc..

[62] Oliveri V (2013). New cyclodextrin-bearing 8-hydroxyquinoline ligands as multifunctional molecules. Eur. J. Chem..

[63] Foggetti G (2019). Autophagy induced by SAHA affects mutant P53 degradation and cancer cell survival. Biosci. Rep..

