# Supplementary material for: Optimized spirooxindole-pyrazole hybrids targeting the p53-MDM2 interplay induce apoptosis and synergize with doxorubicin in A549 cells
Source: Sci Rep. 2023 May 8;13:7441. doi: 10.1038/s41598-023-31209-3 (PMC10167355; doi:10.1038/s41598-023-31209-3)
Supplement: Supplementary file 1 — Supplementary Information. [file 41598_2023_31209_MOESM1_ESM.docx]

**New spirooxindole-pyrazole hybrids targeting the p53-MDM2 interplay as direct MDM2 inhibitors: Design, synthesis, evaluation and Doxorubicin chemosensitization studies**

Mohammad Shahidul Islam,^[a]^ Abdullah Mohammed Al-Majid,^[a]^ Essam Nageh Sholkamy,^[b]^ Assem Barakat, ^[a,*]^ Maurizio Viale,^c^ Paola Menichini,^d^ Andrea Speciale,^d^ Fabrizio Loiacono,^e^ Mohammad Azam,^[a]^ Ved Prakash Verma,^[f]^ Sammer Yousuf,^[g]^ Mohamed Teleb^[h, *]^

^[a]^ Department of Chemistry, College of Science, King Saud University, P.O. Box 2455, Riyadh 11451, Saudi Arabia. Emails: mislam@ksu.edu.sa (M.S.I.); amajid@ksu.edu.sa (A.M.A-M.); [essam_92003@yahoo.com](mailto:essam_92003@yahoo.com) (E.N.S.); [mhashim@ksu.edu.sa](mailto:mhashim@ksu.edu.sa) (M.A.)

^[b]^ Department of Botany and Microbiology, College of Science, King Saud University, P.O. Box 2455, Riyadh, 11451, Saudi Arabia.

^[c]^ U.O.C. Bioterapie, IRCCS Ospedale Policlinico San Martino, Largo R. Benzi 10, 16132 Genova, Italy; Email: [maurizio.viale@hsanmartino.it](mailto:maurizio.viale@hsanmartino.it)

^[d]^ U.O.C. Mutagenesi e Prevenzione Oncologica, IRCCS Ospedale Policlinico San Martino, Largo R. Benzi 10, 16132 Genova, Italy; Email: [paola.menichini@hsanmartino.it](mailto:paola.menichini@hsanmartino.it); [andrea.speciale@hsanmartino.it](mailto:andrea.speciale@hsanmartino.it)

^[e]^ U.O.C. Immunologia, IRCCS Ospedale Policlinico San Martino, Largo R. Benzi 10, 16132 Genova, Italy; Email: [fabrizio.loiacono@hsanmartino.it](mailto:fabrizio.loiacono@hsanmartino.it)

^[f]^ Department of Chemistry, Banasthali Vidyapith, Banasthali-304022, Rajasthan, India. ([vedprakash079@gmail.com](mailto:vedprakash079@gmail.com));

^[g]^ H.E.J. Research Institute of Chemistry, International Centre for Chemical and Biological Sciences, University of Karachi, Karachi 75270, Pakistan; Email: [dr.sammer.yousuf@gmail.com](mailto:dr.sammer.yousuf@gmail.com) (S.Y.)

^[h]^ Department of Pharmaceutical Chemistry, Faculty of Pharmacy, Alexandria University, Alexandria 21521, Egypt. mohamedtelebismail@gmail.com (M.T.).

* Corresponding author: [ambarakat@ksu.edu.sa](mailto:ambarakat@ksu.edu.sa); Tel.: +966-11467-5901; Fax: +966-11467-5992 (A.B.); mohamedtelebismail@gmail.com (M.T.).

Table of Contents

[1 Expermental part 2](#_Toc119401664)

[1.1 General information 2](#_Toc119401665)

[1.2 NMR Spectra of Spiro compounds 8a-p 3](#_Toc119401666)

[1.3 2D NMR analysis for compound 8a 19](#_Toc119401667)

[1.4 LCMS for compound-8a-p 24](#_Toc119401668)

[1.5 X-ray Crystal structure of 8p 29](#_Toc119401669)

[1.6 Structural Features: 30](#_Toc119401670)

[1.7 Crystal Packing: 31](#_Toc119401671)

1.8 Table 3 32

1.9 Dot plost apoptosis 33

2 Immunoblot..................................................................................................................40

# Expermental part

## General information

Phenyhydrazine, acetylacetone, isatin, thioproline, DMF-DMA, NaOH were purchased from Aldrich and used as received. All the substituted aromatic aldehydes were purchased from Aldrich and used as it is. Solvents were used as received when experiments were conducted in air. Flash chromatography was performed on 100-200 mesh silica gel. ^1^H and ^13^C Nuclear Magnetic Resonance (NMR) spectra were recorded on JEOL-400 MHz and JEOL-700 MHz spectrometers at ambient temperature in CDCl_3_ & DMSO-*d*_6_ which were purchased from Sigma Aldrich. Chemical shifts (ppm) are referenced to the residual solvent peak. Coupling constants, *J*, are given in hertz. Abbreviations used in the designation of the signals: s = singlet, d = doublet, dd = doublet of doublets, ddd = doublet of doublet of doublets, dt = doublet of triplets, t = triplet, td = triplet of doublets, m = multiplet. All melting points were measured on a Gallenkamp melting point apparatus in open glass capillaries and are uncorrected. IR Spectra were measured as KBr pellets on a Nicolet 6700 FT-IR spectrophotometer.

## NMR Spectra of Spiro compounds 8a-p

### ^1^H-NMR and ^13^C-NMR for compound-8a

### ^1^H-NMR and ^13^C-NMR for compound-8b

### ^1^H-NMR and ^13^C-NMR for compound-8c

### ^1^H-NMR and ^13^C-NMR for compound-8d

### ^1^H-NMR and ^13^C-NMR for compound-8e

### ^1^H-NMR and ^13^C-NMR for compound-8f

### ^1^H-NMR and ^13^C-NMR for compound-8g

### ^1^H-NMR and ^13^C-NMR for compound-8h

### ^1^H-NMR and ^13^C-NMR for compound-8i

### ^1^H-NMR and ^13^C-NMR for compound-8j

### ^1^H-NMR and ^13^C-NMR for compound-8k

### ^1^H-NMR and ^13^C-NMR for compound-8l

### ^1^H-NMR and ^13^C-NMR for compound-8m

### ^1^H-NMR and ^13^C-NMR for compound-8n

### ^1^H-NMR and ^13^C-NMR for compound-8o

### ^1^H-NMR and ^13^C-NMR for compound-8p

## 2D NMR analysis for compound 8a

Spiro-oxindole **8a** has been characterized by NMR, LCMS and IR spectral analysis. The structure of **8a** has been further established with the help of 2D NMRs (COSY, DEPT, HMQC and HMBC) (**Figure 1**).

Figure 1. Structure of compound 8a

### DEPT-135 NMR analysis:

DEPT-135 spectra of compound **8a** (**Figure 2**) reveals that, there are four +ve signals at δ = 74.74, 64.21, 50.45 and 11.17 ppm and three -ve signals at δ = 53.76 and 36.01 ppm in the aliphatic region. Appearance of those four +ve signals indicate the presence of three CH carbon (C_4_, C_2_, C_3_) bearing one proton each and one CH_3_ carbon (C_7_), while the presence of two -ve signals suggest that, there are two CH_2_ carbons (C_5_, C_6_) bearing even number of protons. The ^13^C and DEPT-135 spectra comparison observation we found that the signal appeared at δ = 74.02 ppm in ^13^C-NMR has been disappeared from the DEPT-135 spectra indicating quaternary carbons C_1_ (**Figure 2**).

### HMQC analysis

HMQC analysis gave us clear picture of specific carbon and proton association. In HMQC spectra we observed that C_1_ carbon does not have any proton association to itself, but C_4_, C_2_ and C_3_ carbons bearing single proton each of them H_3_, H_1_, and H_2_ respectively, while C_7_ carbon attached to 3 equivalent protons (H_8_). However, C_5_ and C_6_ these two carbons are attached to two protons each (C_6_-H_6,7_; C_5_-H_4,5_) (**Figure 3**) which was further proven from DEPT analysis as there are four +ve and two -ve signals of carbons each of them bearing uneven and even number of protons respectively (**Figure 2**) that confirmed the structure of **8a**.


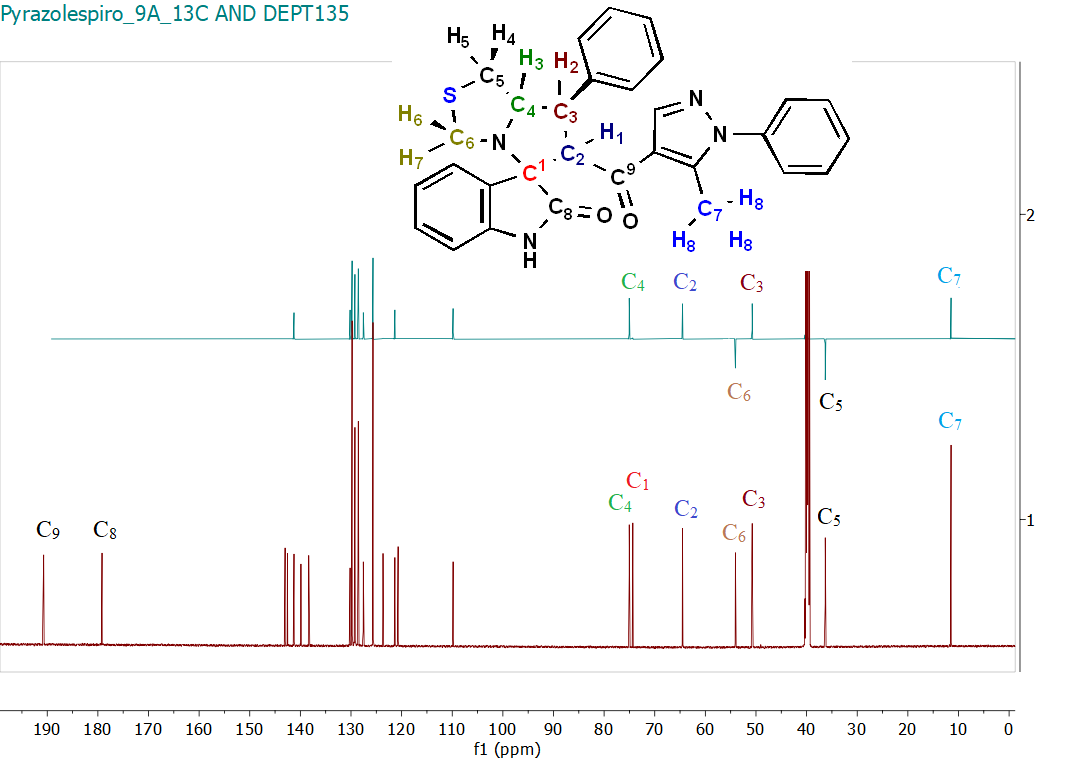
**Figure 2.** DEPT-135 NMR of compound **8a.**


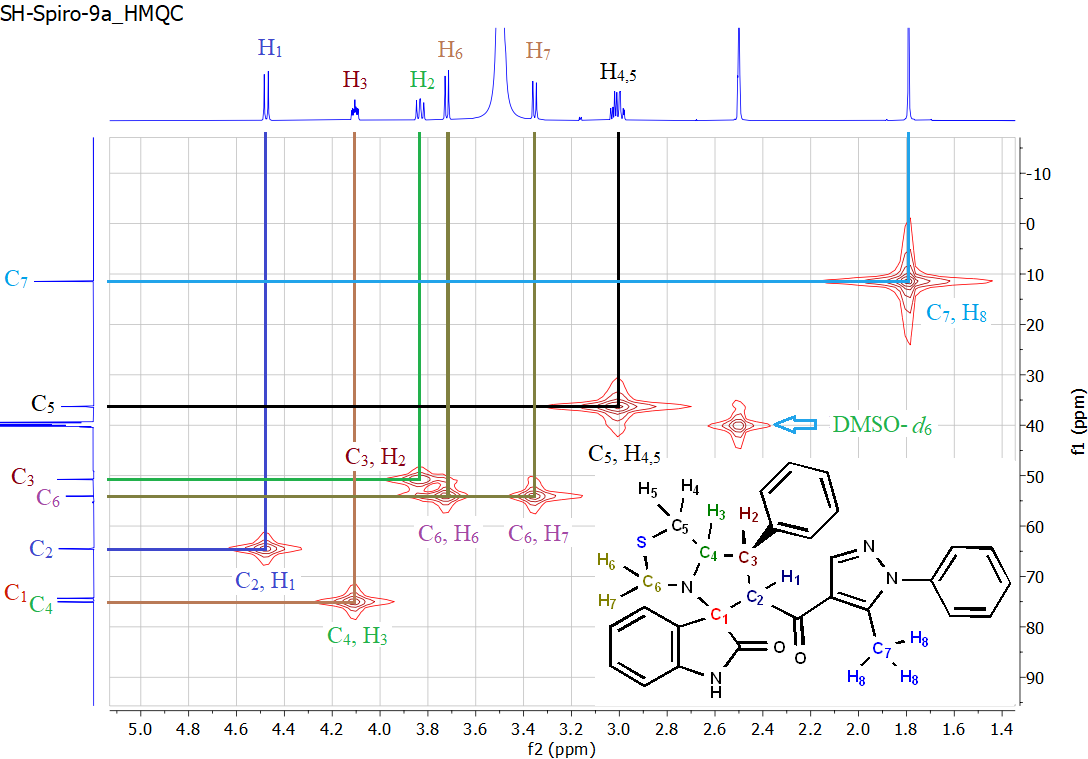


Figure 3. HMQC NMR of compound 8a.

### HMBC analysis

The HMBC spectra (**Figure 4**) reveals that, the H_1_ proton is having 1,3 and 1,2 C-H interaction with carbons C_8_=O, Ar-C (3), C_4_, and C_9_=O, C_1_, C_3_ respectively (shown in **Figure 3.5** blue arrows). The H_2_ proton is having 1,3 and 1,2 C-H interaction with carbons C_9_=O, Ar-C, C_1_, C_5_, and Ar-C, C_2_, C_4_ respectively (shown in **Figure 4** red arrows). The H_3_ proton is having 1,3 and 1,2 C-H interaction with carbons C_1_, C_6_, Ar-C and C_3_, C_5_ respectively (shown in **Figure 4** pink arrows). The H_4,5_ protons are having 1,3 and 1,2 C-H interaction with carbons C_3_, C_6_, and C_4_, respectively. The H_6_,_7_ protons are having only 1,3 C-H interaction with carbons C_1_, C_4_ and C_5_ (shown in **Figure 4** bright green arrows). The NH proton is having 1,3 and 1,2 C-H interaction with carbons C_1_, Ar-C (2) and Ar-C, C_8_=O respectively (shown in **Figure 4** dark green arrows).

Figure 4. HMBC shows 1,2 and 1,3 C-H interaction.

### ^1^H-^1^H-COSY NMR analysis:

After identifying specific carbon and proton association and their position from the above discussion of DEPT, HMQC and HMBC, finally ^1^H-^1^H-COSY NMR mapping analysis helps us to find the neighbouring protons association (H-C-C-H). From the COSY spectra (**Figure 6**) of spiro compound **8a**, it is clearly evident that the H_1_ and H_2_ are coupled to each other, while the proton H_2_ coupled with another protons H_3_. Proton H_3_ further coupled with other two protons H_4_ and H_5_. Protons H_6_ and H_7_ are also coupled with each other. Therefore, it can be concluded that, proton H_1_ and H_2_ are neighbour to each other and proton H_3_ is having three neighbouring protons H_2_, H_4_ and H_5_, while H_4_ and H_5_ are having only one neighbouring protons H_3_. It is also clear that the protons H_6_ and H_7_ are adjacent to each other attached to single carbon.


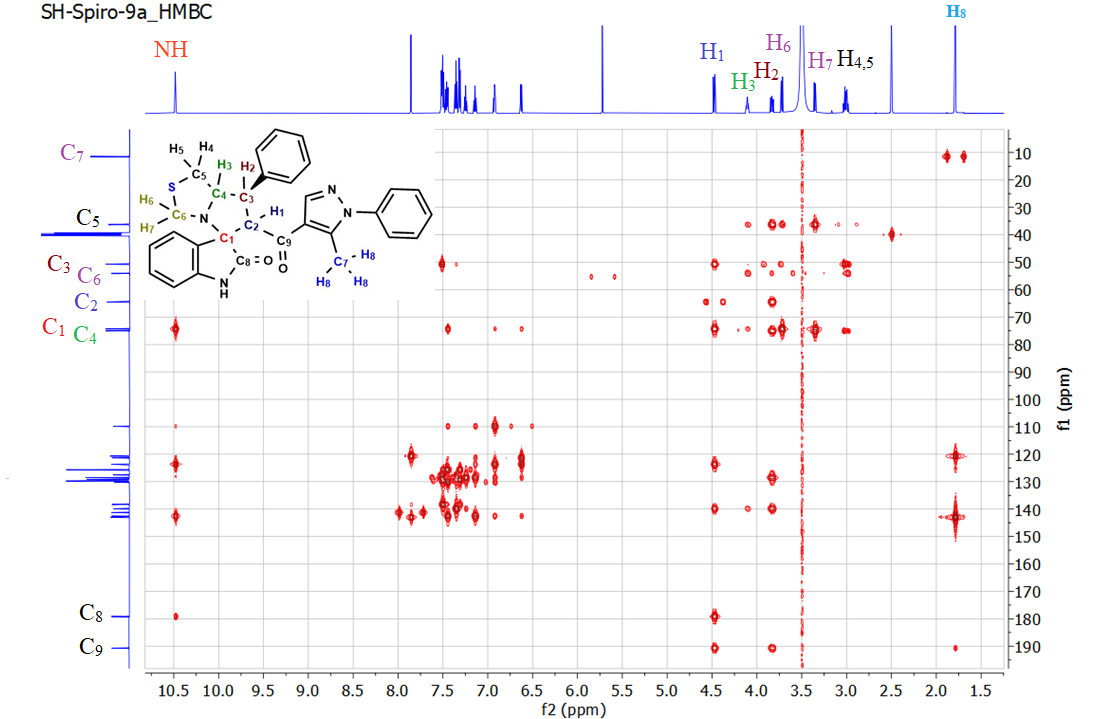


Figure 5. HMQC full spectrum of compound 8a.


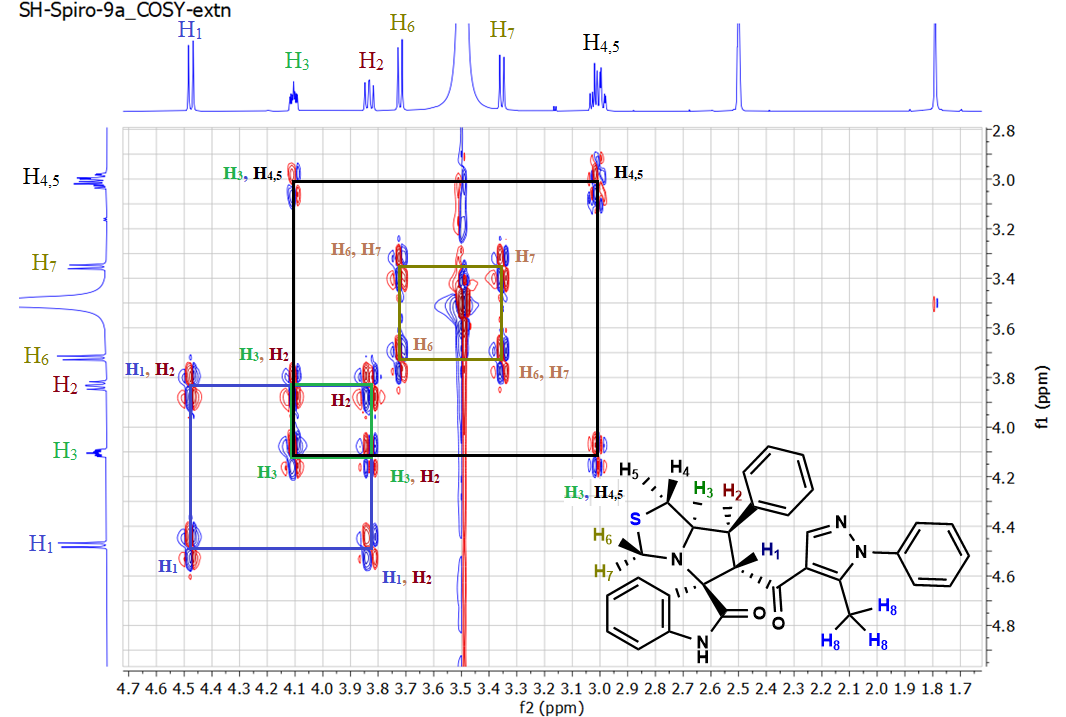


Figure 6: ^1^H-^1^H-COSY NMR of compound 8a

### ^1^H and ^13^C-NMR analysis of compound 8a

Finally, after 2D NMR analysis protons and carbons have been assigned in the ^1^H and ^13^C-NMR (**Figure 7 and 8**).


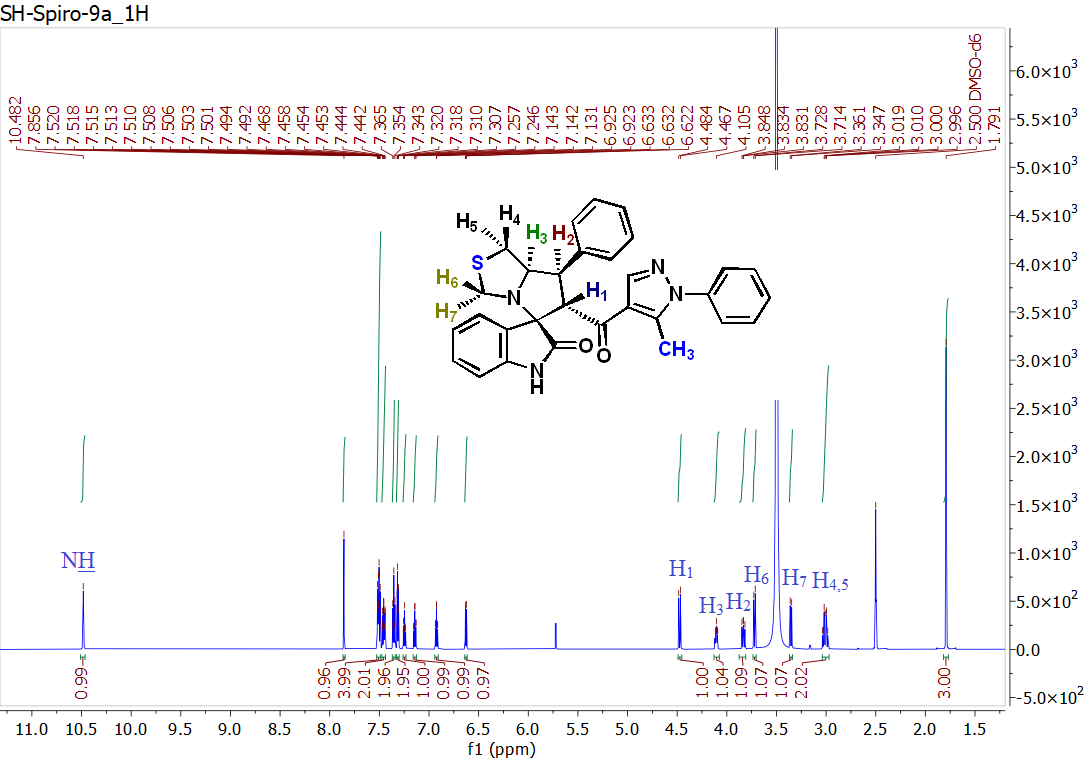


Figure 7. ^1^H-NMR of compound 8a


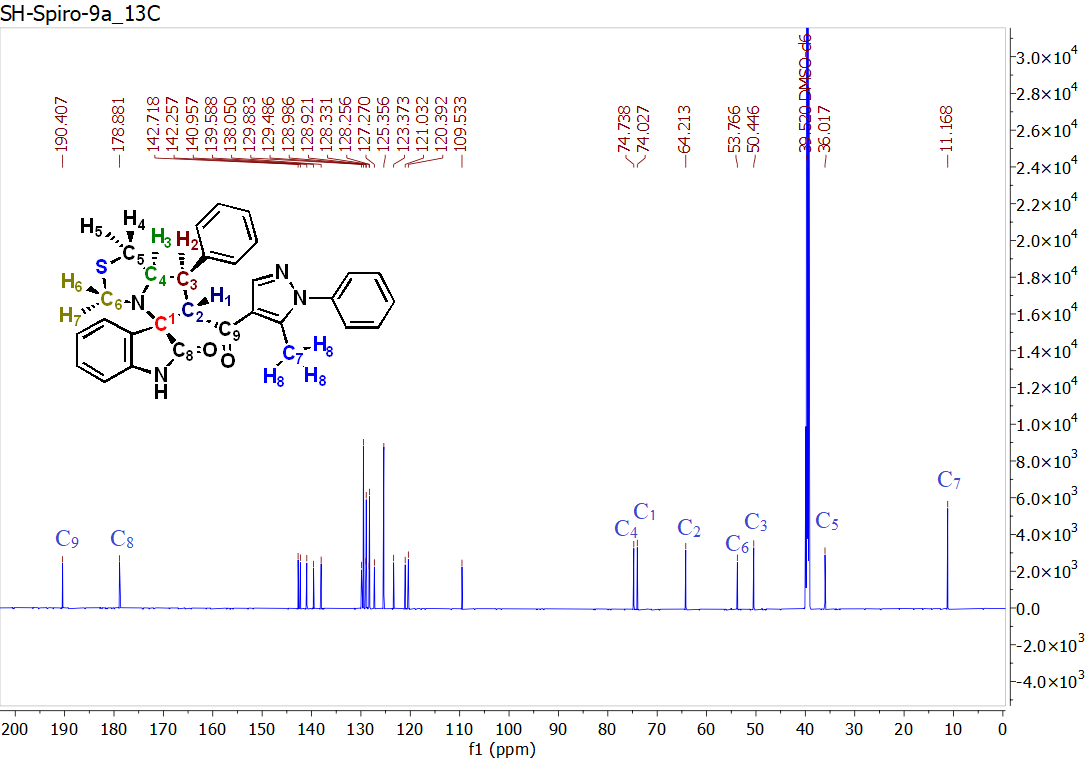


Figure 8. ^13^C-NMR of compound 8a

## LCMS for compound-8a-p

### LCMS for compound-8a


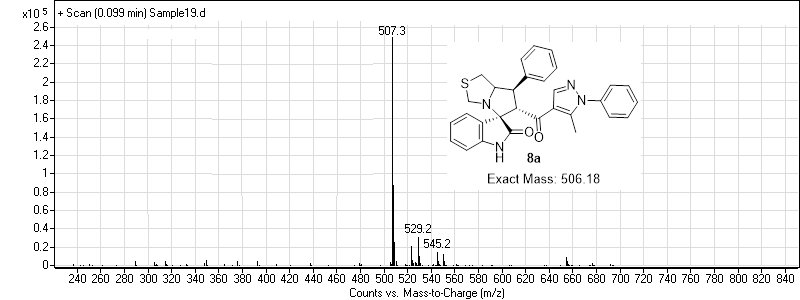


### LCMS for compound-8b


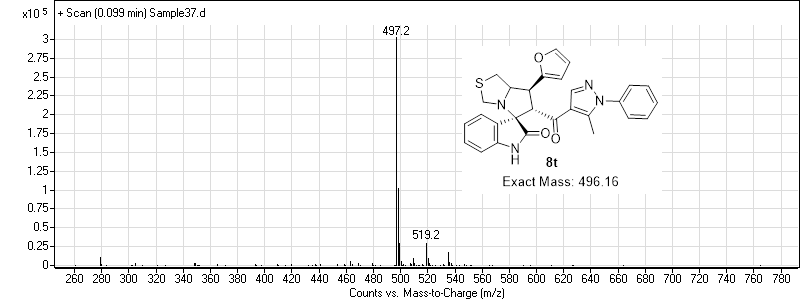


### LCMS for compound-8c


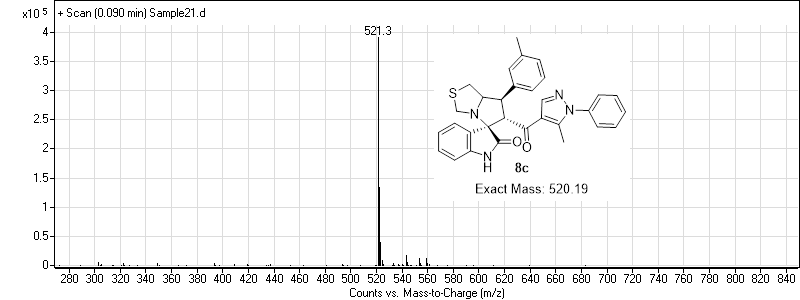


### LCMS for compound-8d


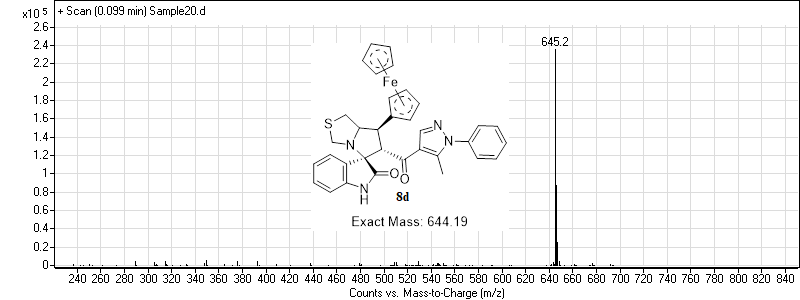


### LCMS for compound-8e


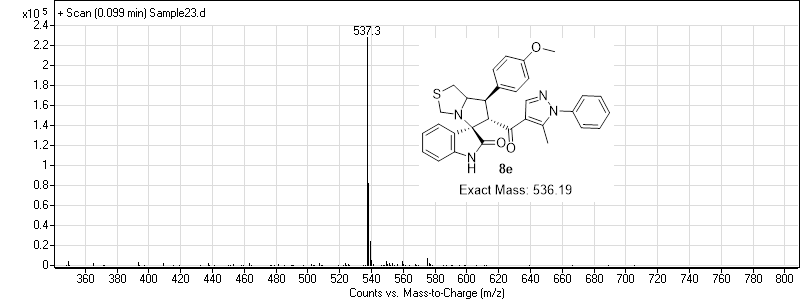


### LCMS for compound-8f


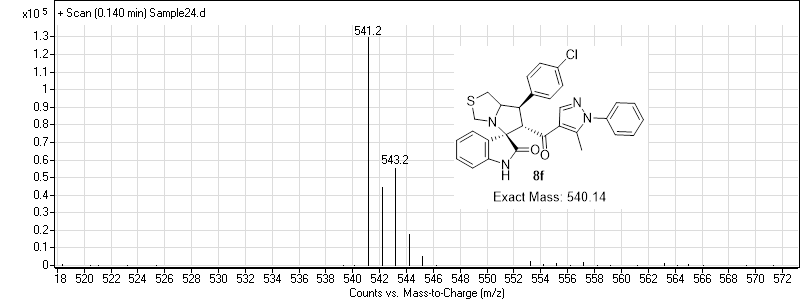


### LCMS for compound-8g


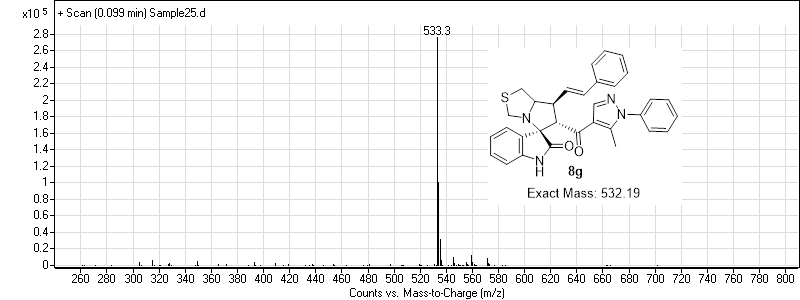


### LCMS for compound-8h


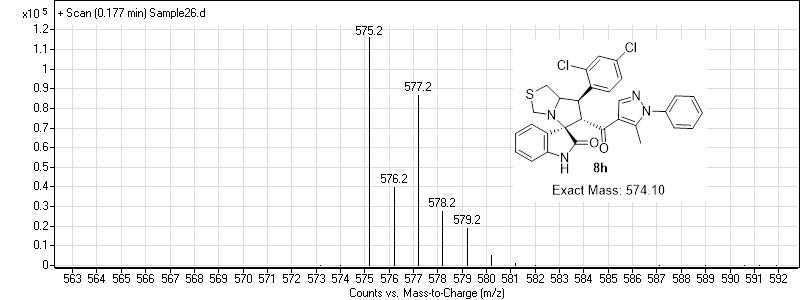


### LCMS for compound-8i


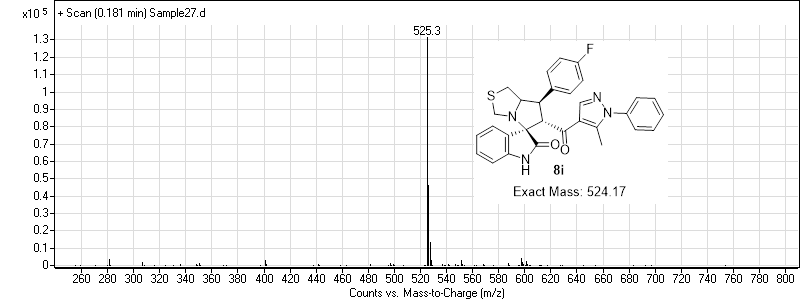


### LCMS for compound-8j


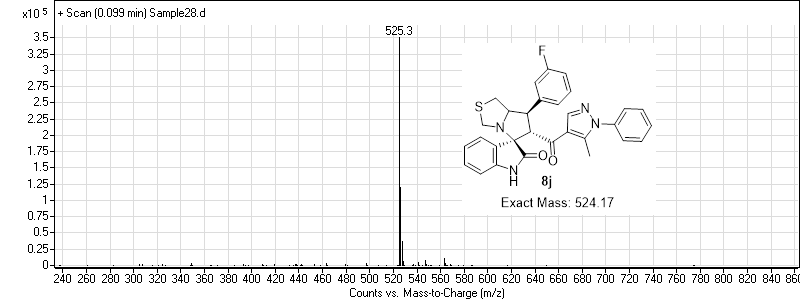


### LCMS for compound-8k


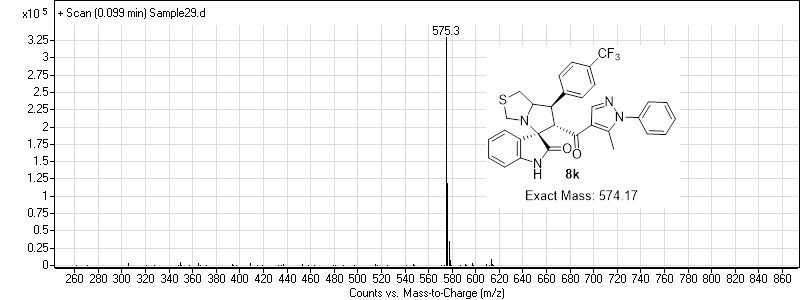


### LCMS for compound-8l


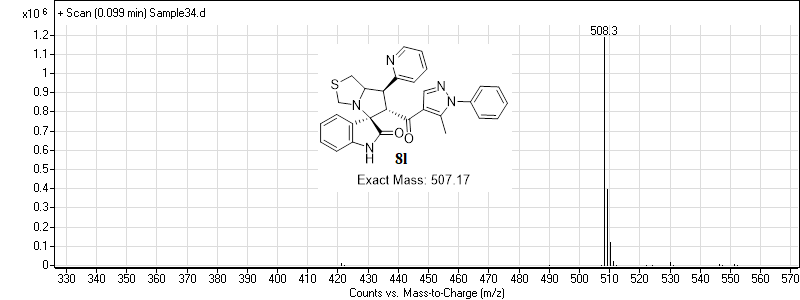


### LCMS for compound-8m


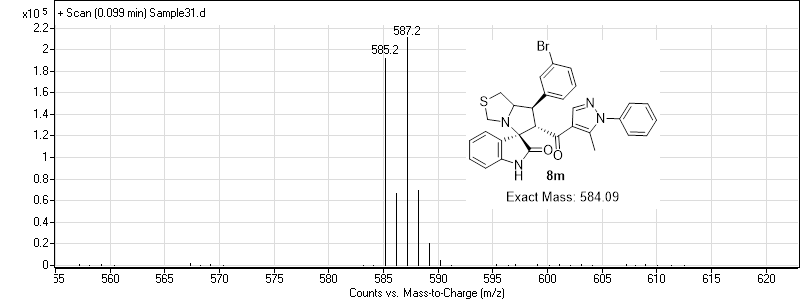


### LCMS for compound-8n


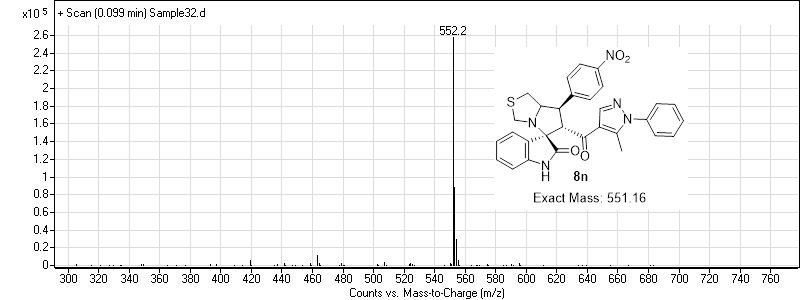


### LCMS for compound-8o


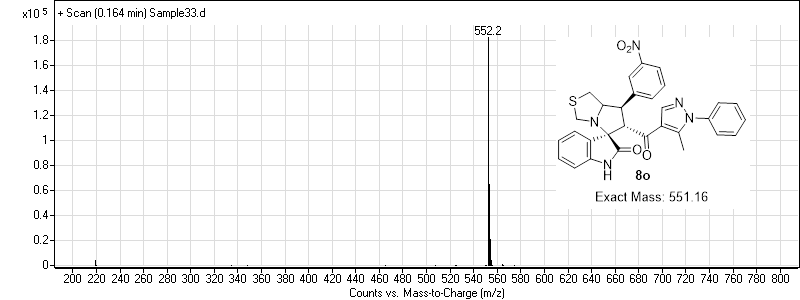


### LCMS for compound-8p


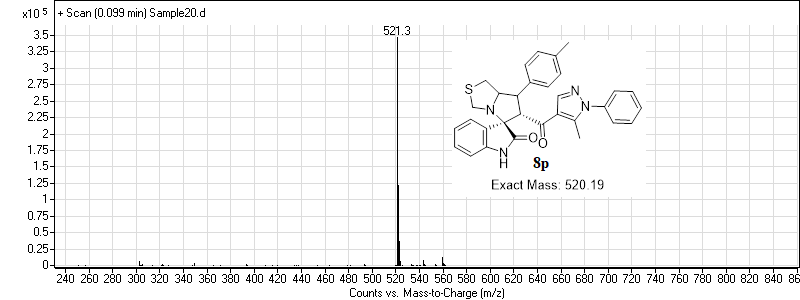


## X-ray Crystal structure of 8p

**Instrumentation:**

Plate-shaped crystal of the targeted molecule (**8p**) was mounted on a single crystal X-ray diffractometer. The crystal data were collected on Bruker APEX D8 Venture which is fitted with [PHOTON 100 detector](https://www.bruker.com/products/x-ray-diffraction-and-elemental-analysis/single-crystal-x-ray-diffraction/sc-xrd-components/sc-xrd-components/overview/sc-xrd-components/detectors.html) having CMOS technology with Cu Kα (λ = 1.54178 Å) radiation as an X-ray source. Reflection intensities were further integrated and reduced by Bruker SAINT Software (Bruker, 2004). The structure was solved by applying the direct method followed by final refinement *via* crystallographic packages SHELX 97 (Sheldrick, 1997) and SHELXL (Sheldrick 2015). The graphical overview of title compound was drawn by using *ORTEP3* (Farrugia, 2012), while inter-molecular hydrogen bond geometries and three-dimensional crystal packing were carried out through *PLATON* (Spek 2002) and *Mercury* 3.10.1 program, respectively (Macrae *et al*., 2008).

**Table S1:** Experimental crystallographic data of title compound (**8p**)

| **Properties** | Compound **8p** |
| --- | --- |
| CCDC No. | 2177096 |
| Molecular Formula | C_62_ H_56_ N_8_ O_4_ S_2_ |
| Formula weight | 1041.26 |
| Temperature | 104(2) K |
| Wavelength | 1.54178 A |
| Crystal system | Orthorhombic |
| Space group | *Pca2_1_* |
| Unit cell dimensions | a = 19.5088 (7) *α* =90° |
|  | b = 9.8600 (4) *β* = 90° |
|  | c = 27.6216(10) *γ* = 90° |
| Volume | 5313.2 (3) Å^3^ |
| Z | 4 |
| Density (calculated) | 1.302 g/cm^3^ |
| Absorption coefficient | 1.366 mm^-1^ |
| F(000) | 2192 |
| Crystal size | 0.230 x 0.090 x 0.080 mm |
| Theta range for data collection | 3.200 to 68.162° |
| Index ranges | -23<=h<=20  -11<=k<=11  -33<=l<=33 |
| Reflections collected | 37038 |
| Independent reflections | 9470 |
| Completeness to theta = 67.679° | 100.0 % |
| Refinement method | Full-matrix least-squares on F^2^ |
| Data / restraints / parameters | 9470 / 1 / 693 |
| Goodness-of-fit on F^2^ | 0.985 |
| Final R indices [I>2sigma(I)] | R1 = 0.0457  wR2 = 0.0905 |
| R indices (all data) | R1 = 0.0711  wR2 = 0.0992 |
| Extinction coefficient | 0.027(16) |
| Largest diff. peak, and hole | 0.233 and -0.256 e.Å-^3^ |

## Structural Features:





Figure 9. *ORTEP* view of compound 8p drawn at 50% probability level

The crystal data of title compound C_62_ H_56_ N_8_ O_4_ S_2_ were collected in orthorhombic spacegroup *Pca2_1_* having two independent molecules in an asymmetric unit. In molecular skeleton, a central pyrrolo[1,2-c]thiazol moiety (S1/N3/C12/C13/C21-C24) fused with an oxyindoline ring i.e. ring F (C25-C30) and G (O2/N4/C24-C26/C31) *via* C4-spiro atom. The thiazolidine ring of central moiety (S1/N3/C21-C23) adopts envelope geometry with puckering amplitude of *Q* = 0.503(4) Å and φ = 359.2(5) °, while pyrrolidine ring (N3/C12/C13/C21/C24) exist in twist conformation having puckering amplitude of 0.404 (5) Å and φ = 96.7 (6) °. The central ring was also substituted with ring D (C14-C20) at C13. The oxyindoline moiety is inclined at a dihedral angle of 30.37° with planar ring D (C14-C20) showing r.m.s deviation of 0.025 Å (C31), 0.023 Å (C24), and 0.012 Å (C27). The N-phenyl (C1-C6) substituted pyrazole moiety (N1/N2/C7-C9) also linked with the central ring *via* C10/O1 ketonic bridge at C12 (**Table-S1**).

## Crystal Packing:

Supramolecular features are dominated by conventional as well as non-conventional hydrogen bonds. Inside the crystal lattice molecules are associated *via* three C-H…O type intermolecular contacts including C29-H29…O4, C51-H51A…O1, and C60-H60…O2 (**Table-2**). In addition, conventional N8-H1A…S1, N4-H4…O4, and N4-H4…N7 interactions that interlinked neighboring molecules in an infinite chain running along *b* axis (**Figure 10**).


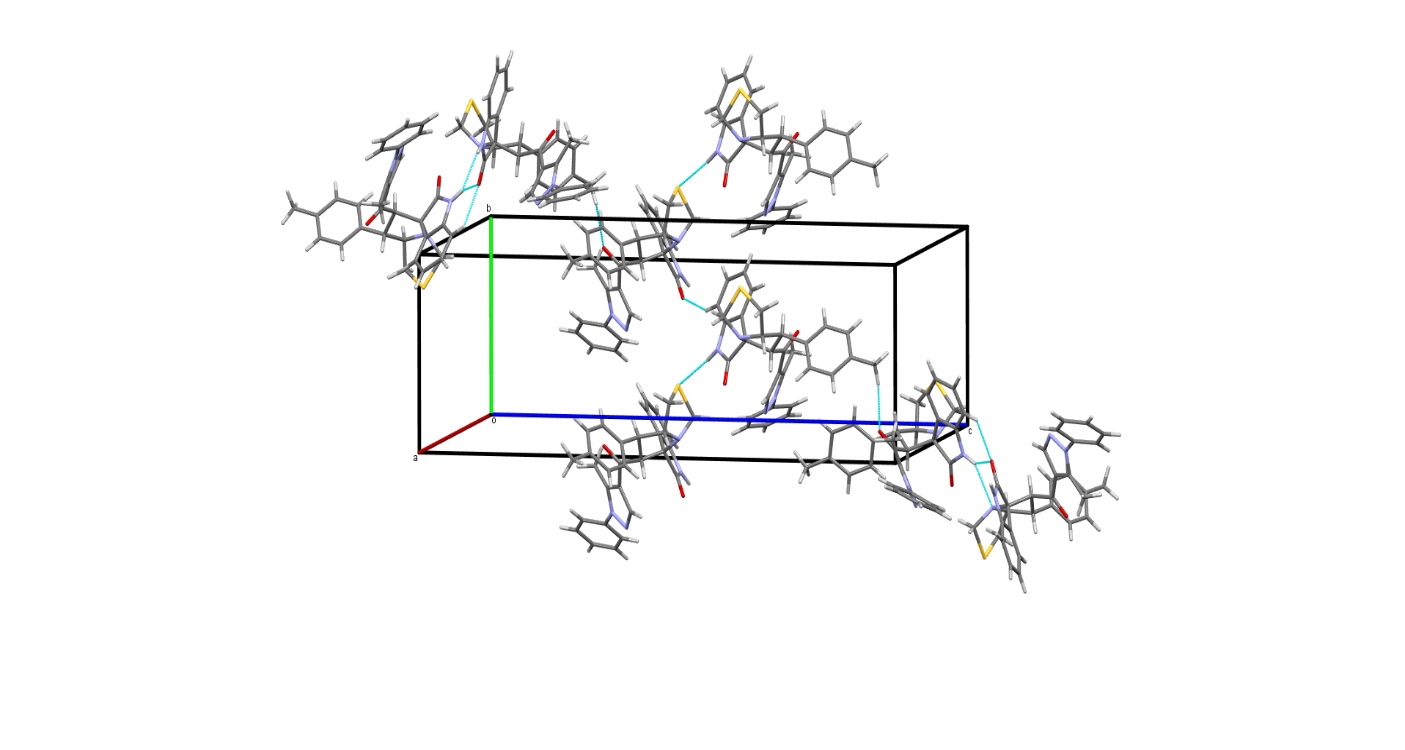


Figure 10.Crystal packing diagram of compound (8p)

### Table S2: Hydrogen bonding of compound (8p).

| D | H | A | D-H | H…A | D…A | D-H…A |
| --- | --- | --- | --- | --- | --- | --- |
| N8 | H1A | S1 | 0.97 (6) | 2.37 (6) | 3.320 (4) | 166 (5) |
| N4 | H4 | O4 | 0.88 | 2.34 | 2.999 (5) | 132 |
| N4 | H4 | N7 | 0.88 | 2.43 | 3.201 (5) | 146 |
| C29 | H29 | O4 | 0.95 | 2.28 | 3.026 (6) | 135 |
| C51 | H51A | O1 | 0.98 | 2.47 | 3.440 (7) | 169 |
| C60 | H60 | O2 | 0.95 | 2.38 | 3.224 (5) | 147 |

**Table S3.** Antiproliferative activity of doxorubicin (nM) against the panel of four human cancer cell lines

## Figure dot plots apoptosis

**
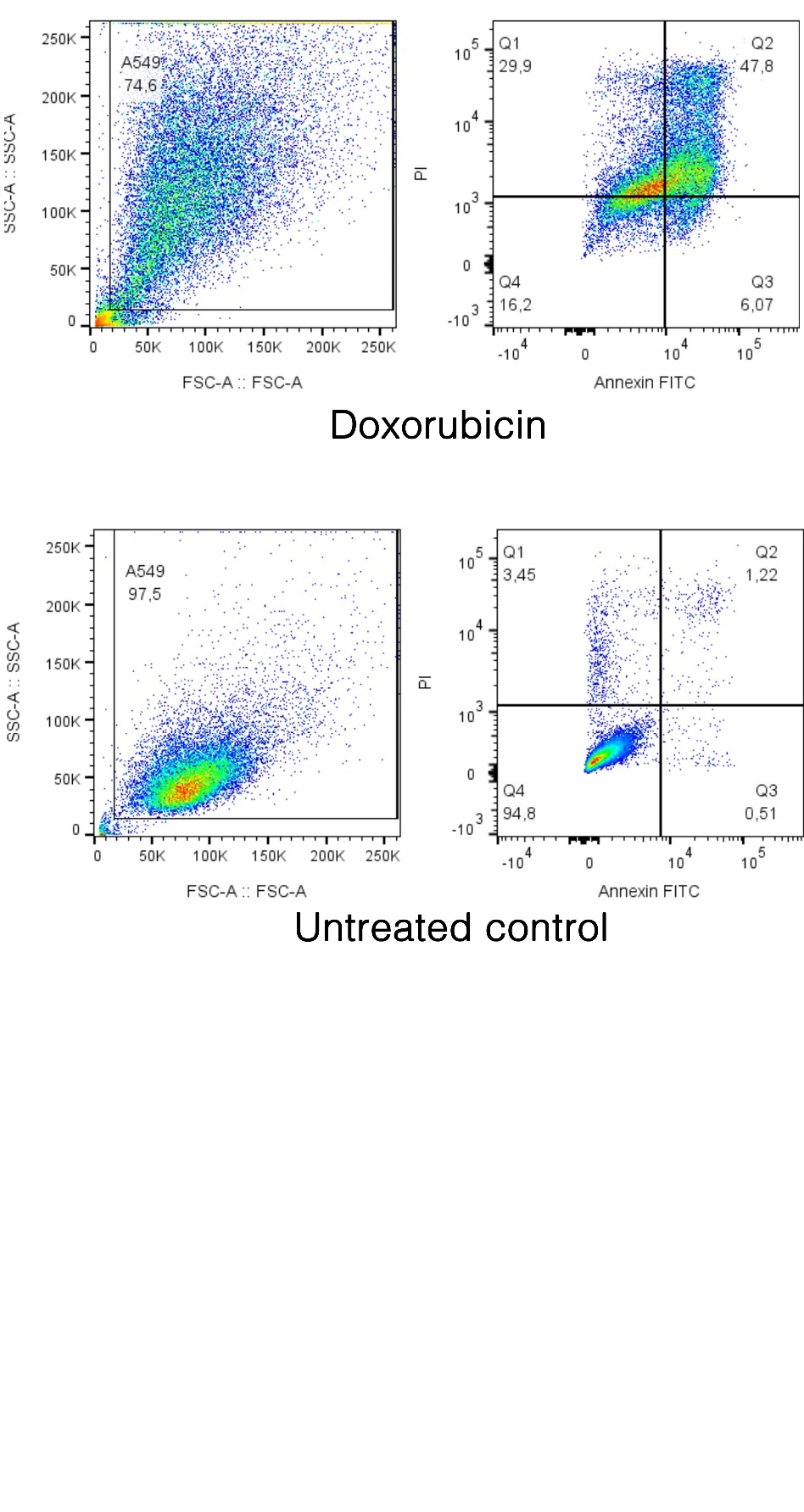
**

**
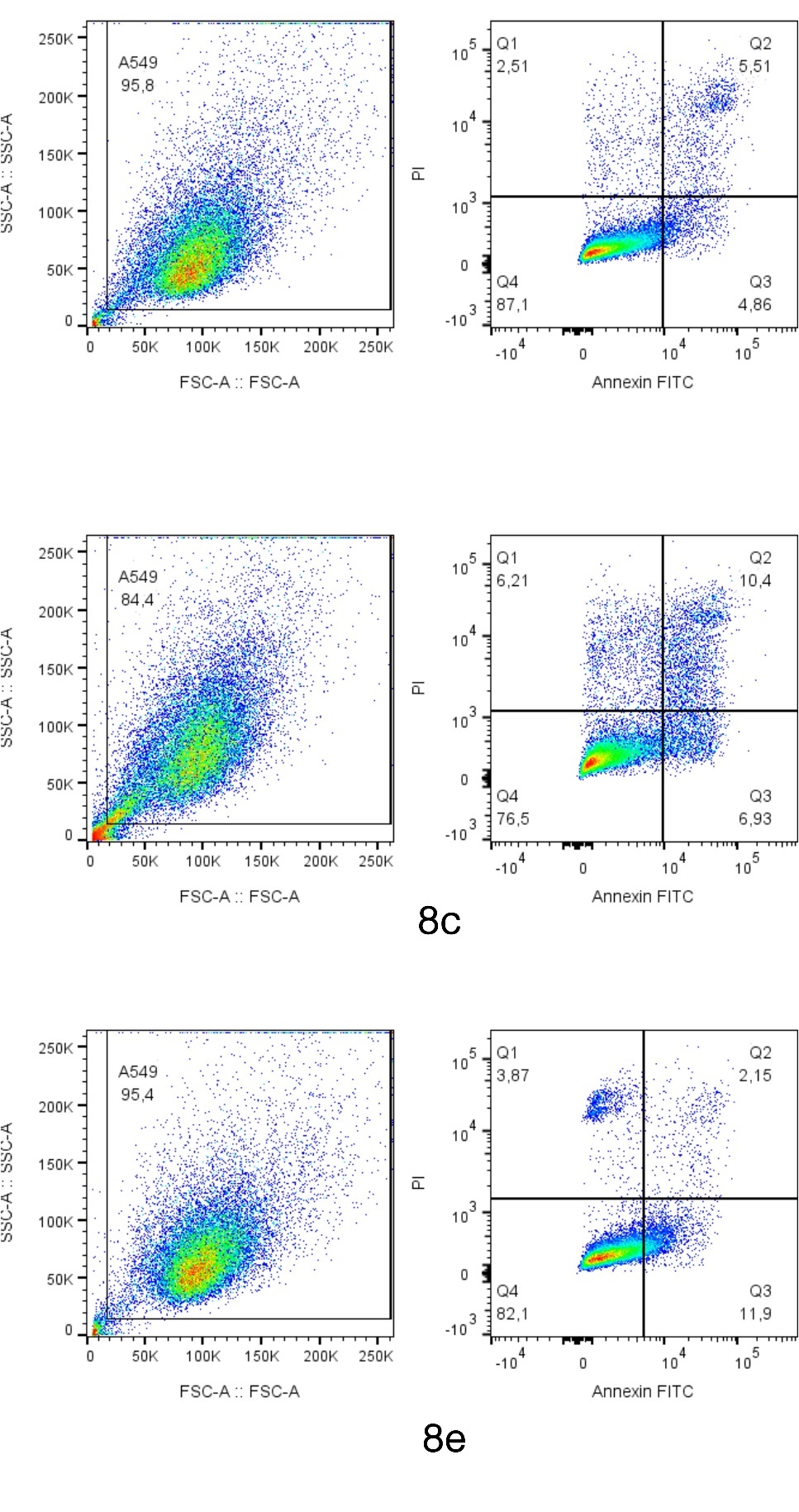
**

**
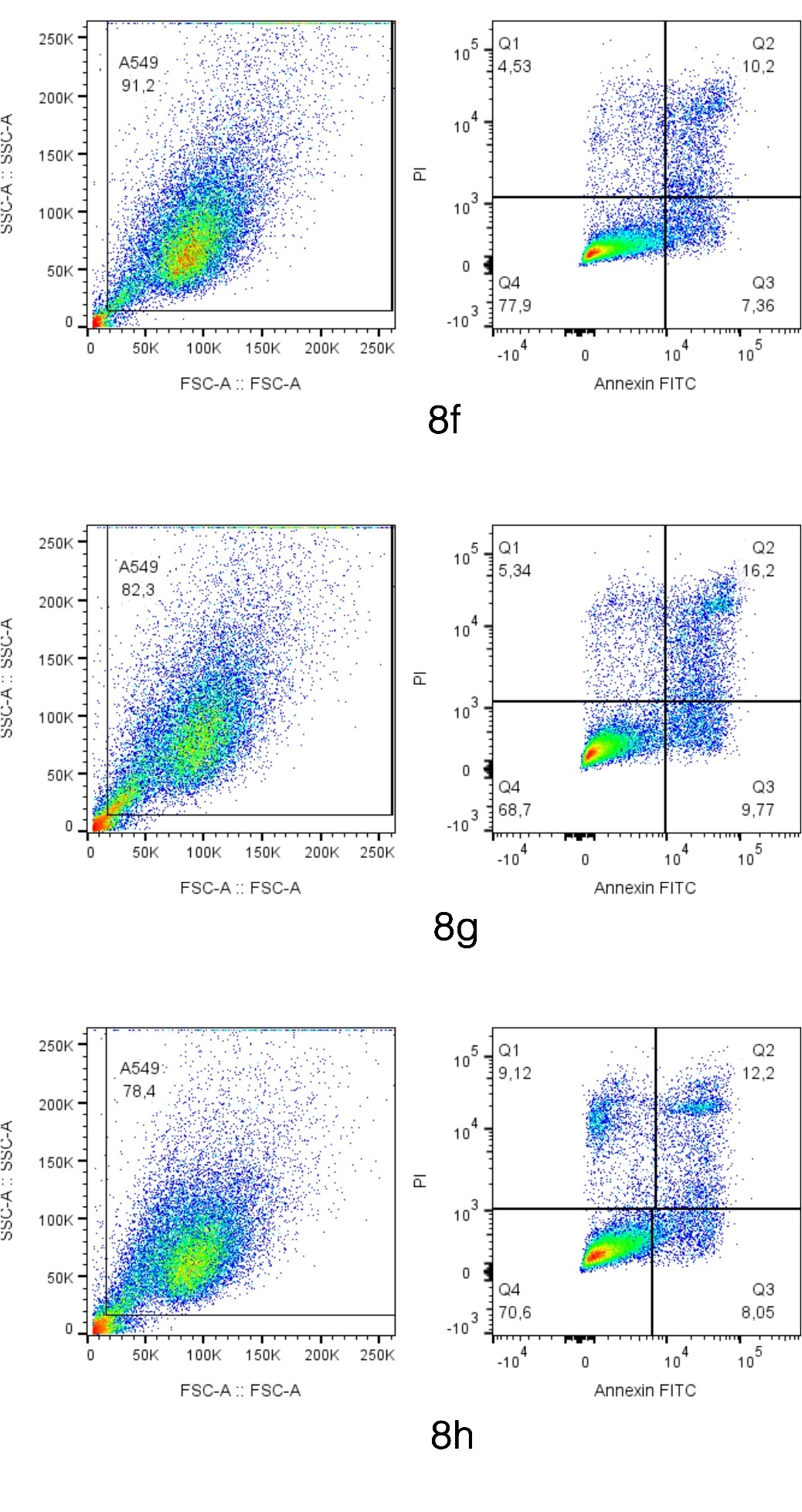
**

**
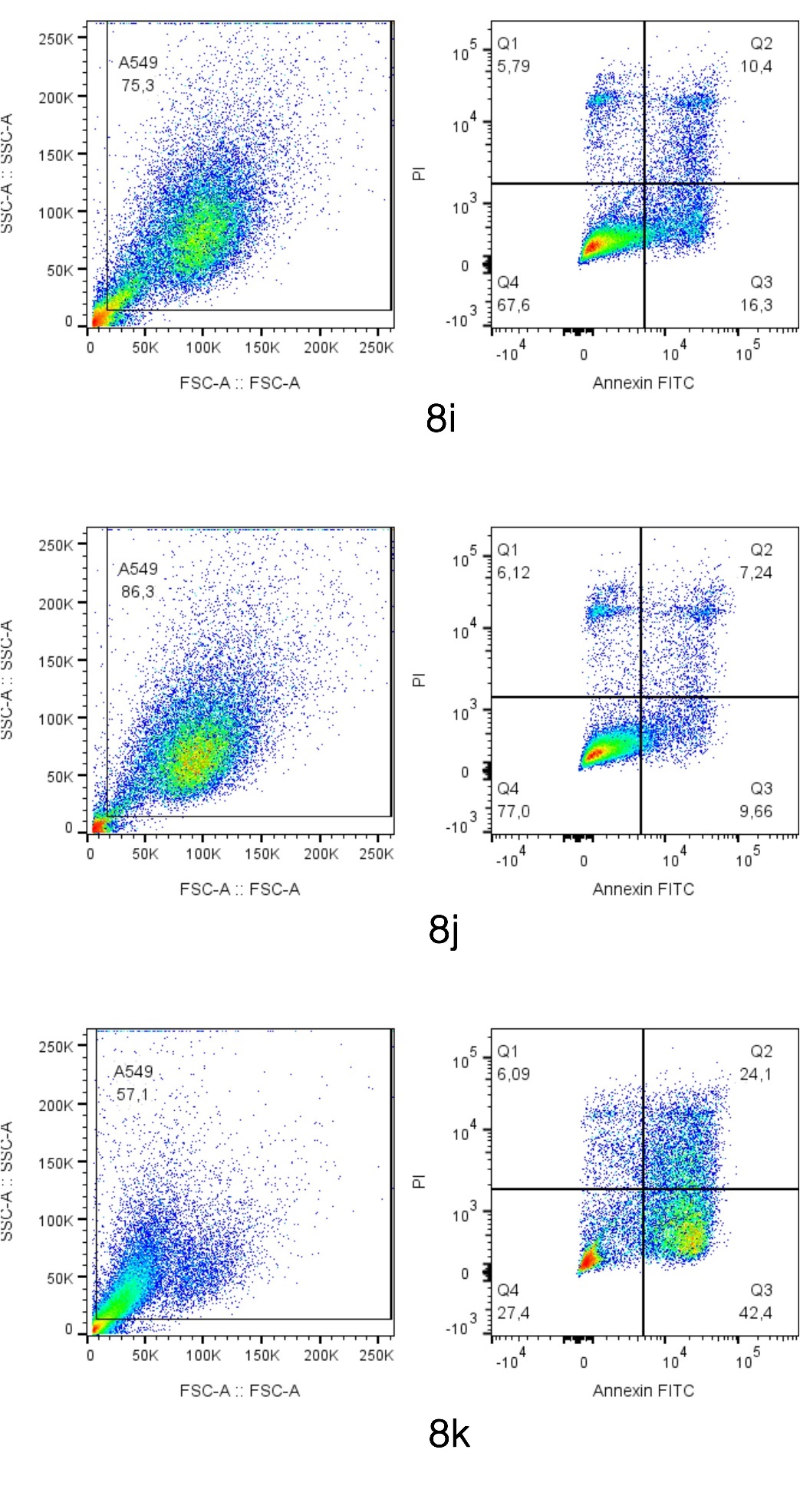
**

**
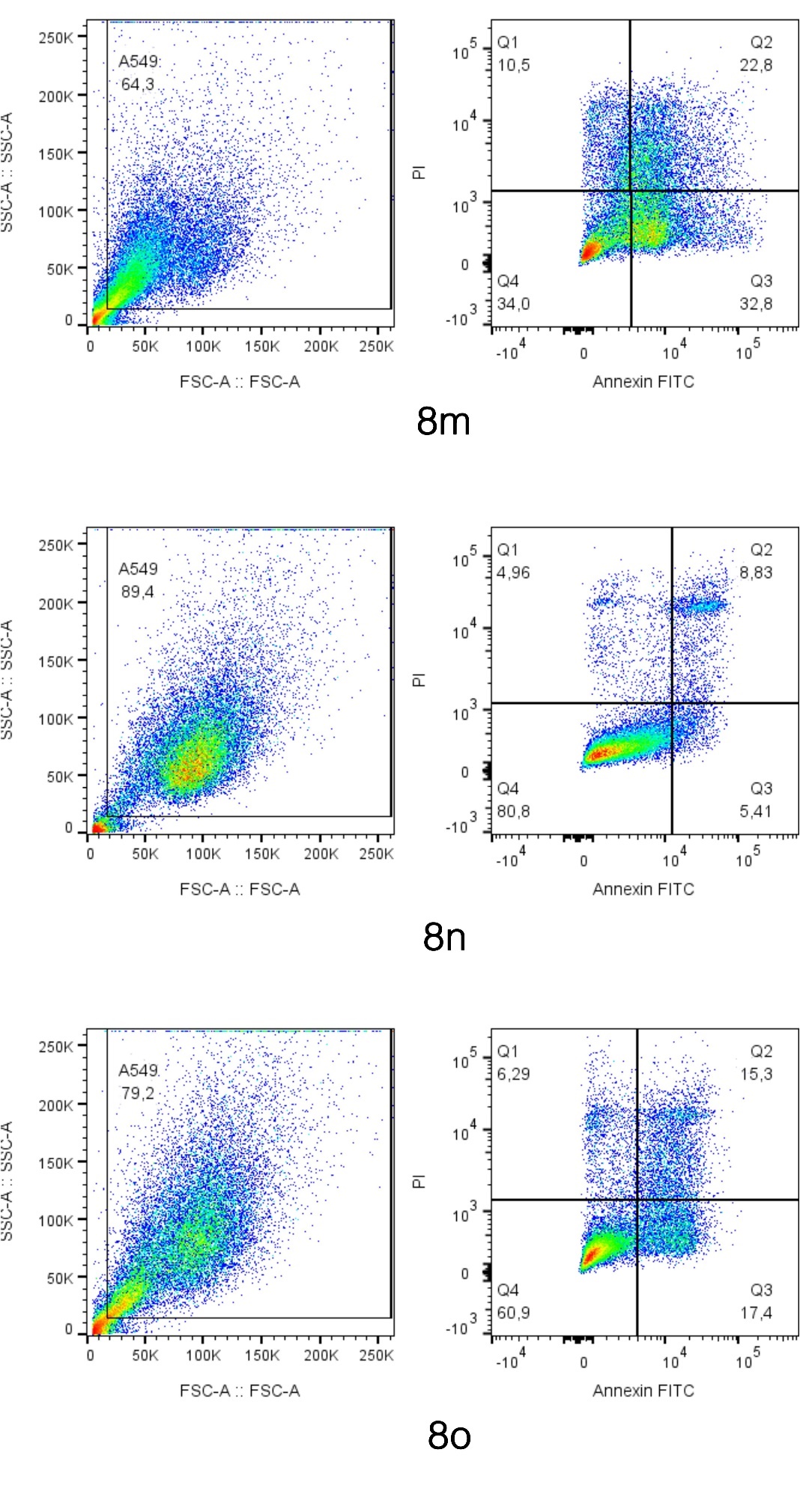
**

**
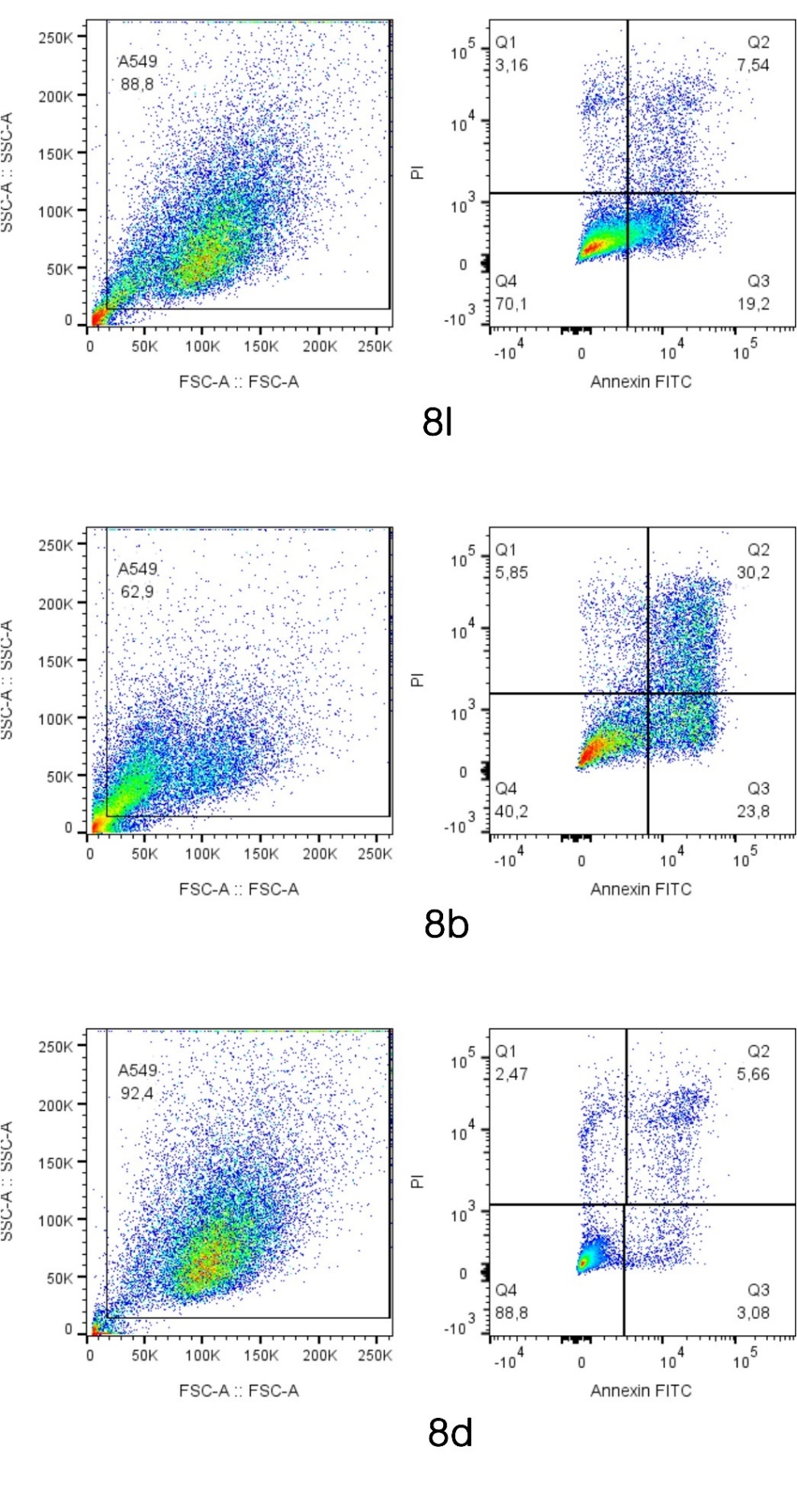
**

**Figure 1.7.** Plots show a rapresentative view of the effect of the tested molecules.

Note that the quadrant statistics are positioned based on the control samples and are similar but not equal to each other (releted to the specific controls of the experimental day). The different treatments are rapresented by the phisical parameters first followed by the annexin/PI assay. The phisical parameters already show the morfological alteration of cells after treatments.

**2. Original immunoblot**

**Figure S6 (**Supplementary information to Fig. 6. Uncropped original blots of Figure 6. A, the whole membrane has been cut in two parts (up & down) and incubated with different antibodies as indicated; B, merge with Molecular weight markers; C, a picture of the whole membrane following the last incubations has been taken for reference

**
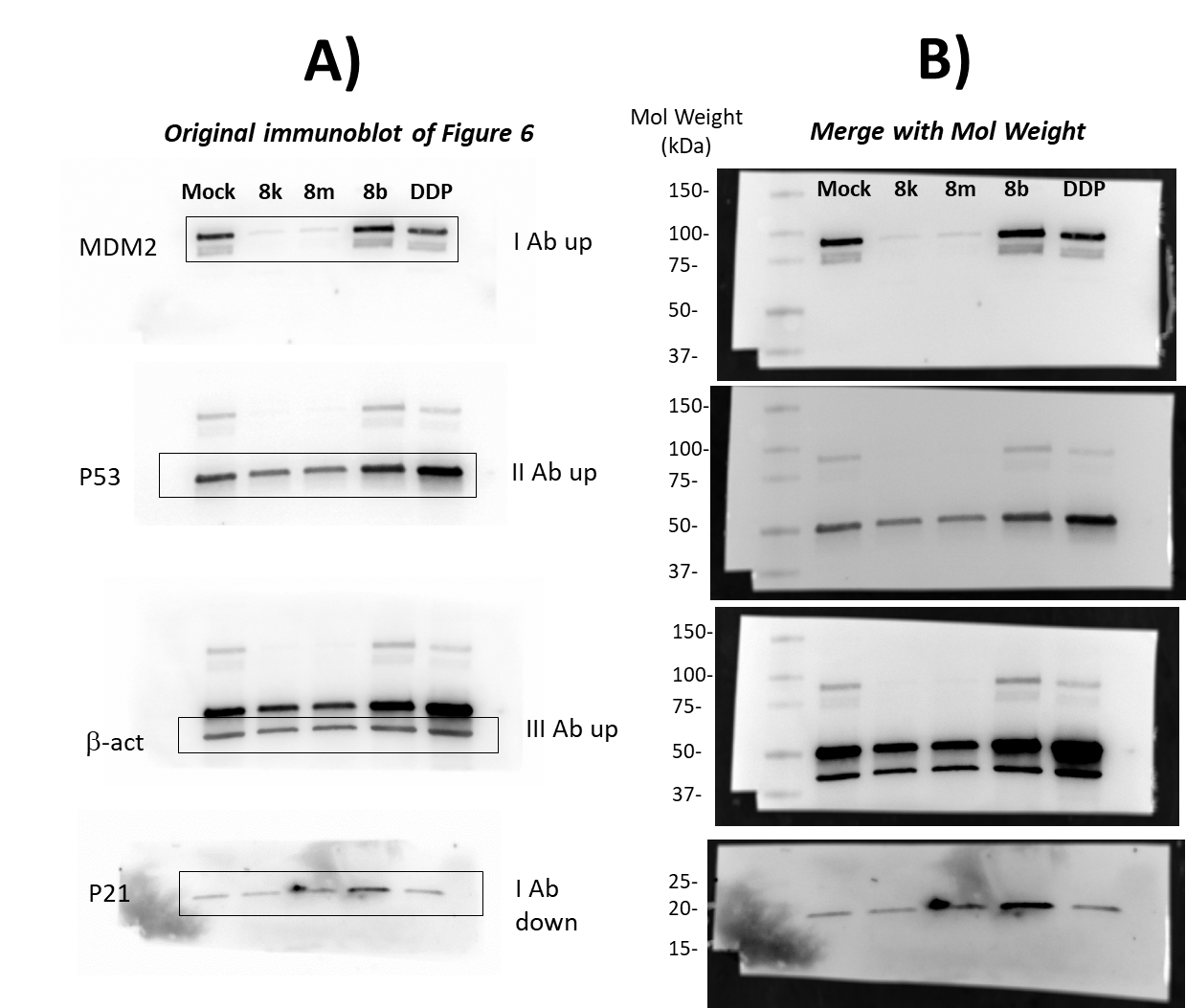
**

**
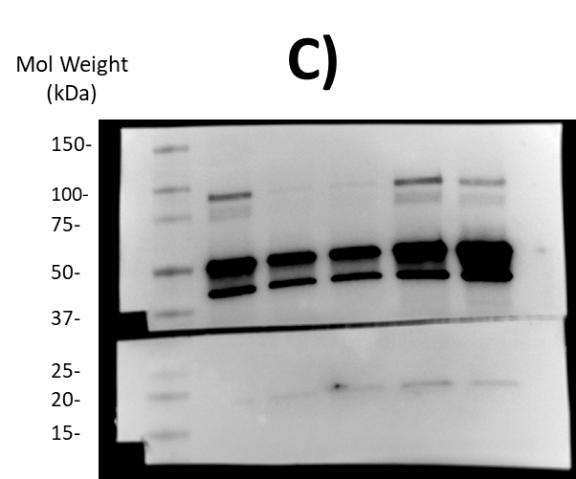
**

**Figure S7** (Supplementary information to Fig. 7. Uncropped original blots of Figure 7. A, the membrane has been incubated with different antibodies as indicated; B, merge with Molecular weight markers

**
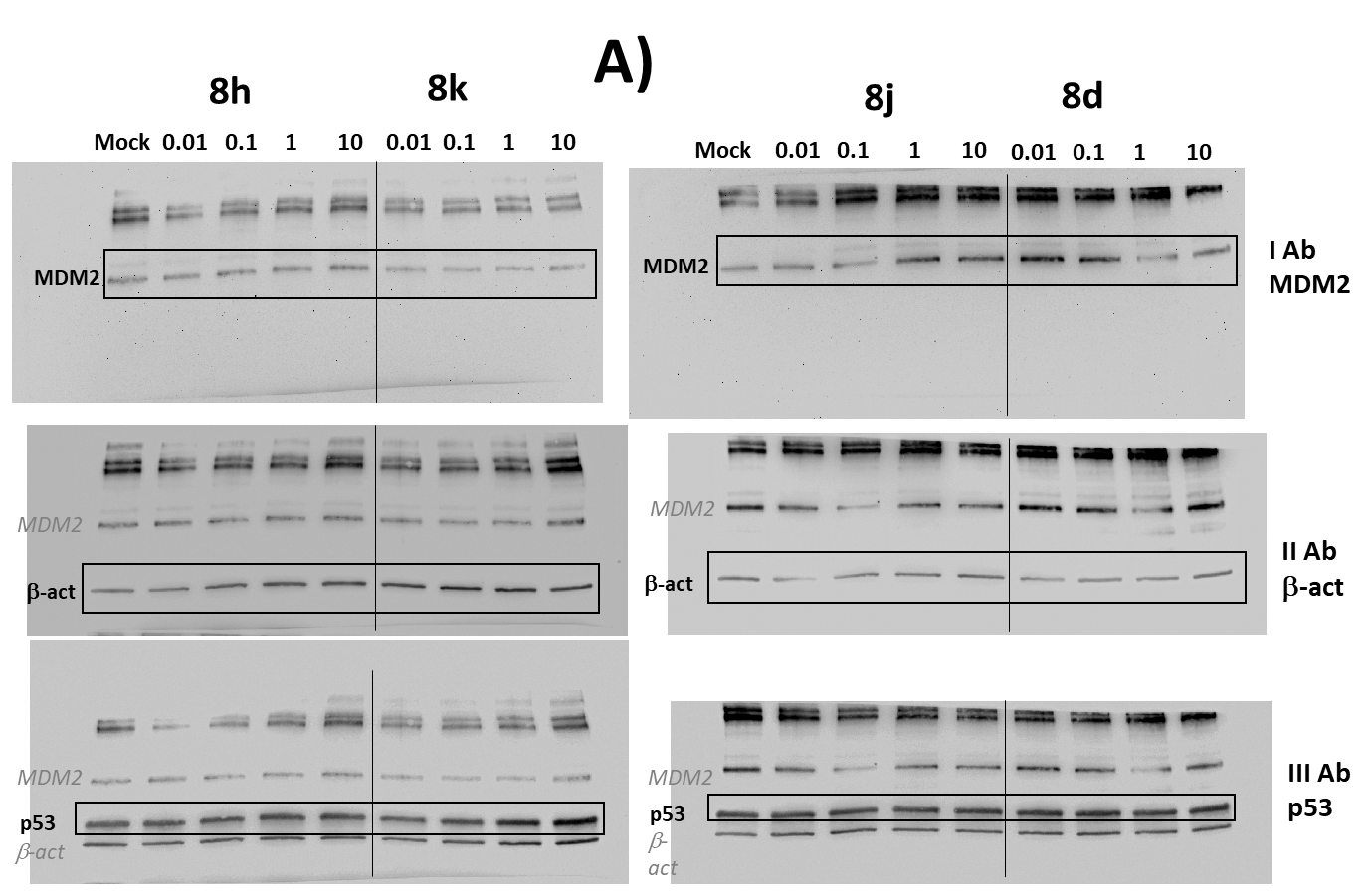
**

**
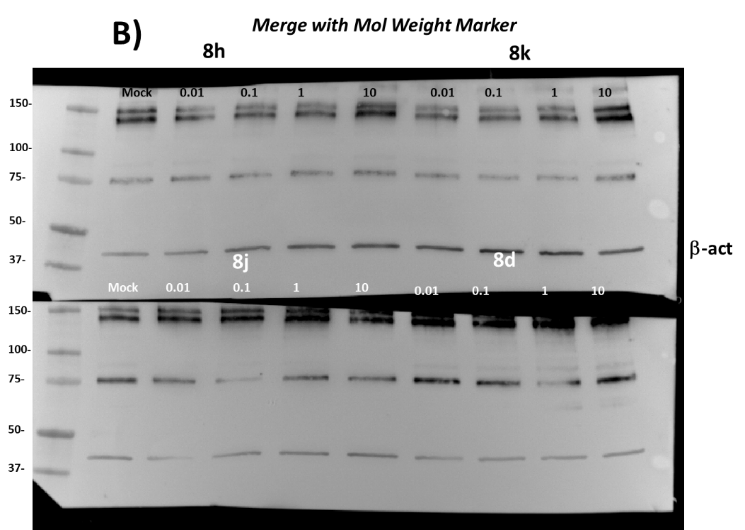
**

**Figure S8** (Supplementary information to Fig. 8. Uncropped original blots of Figure 8. A, the whole membrane has been cut in two parts (up & down) and incubated with different antibodies as indicated; B, merge with Molecular weight markers; C, a picture of the whole membrane following the last incubations has been taken for reference


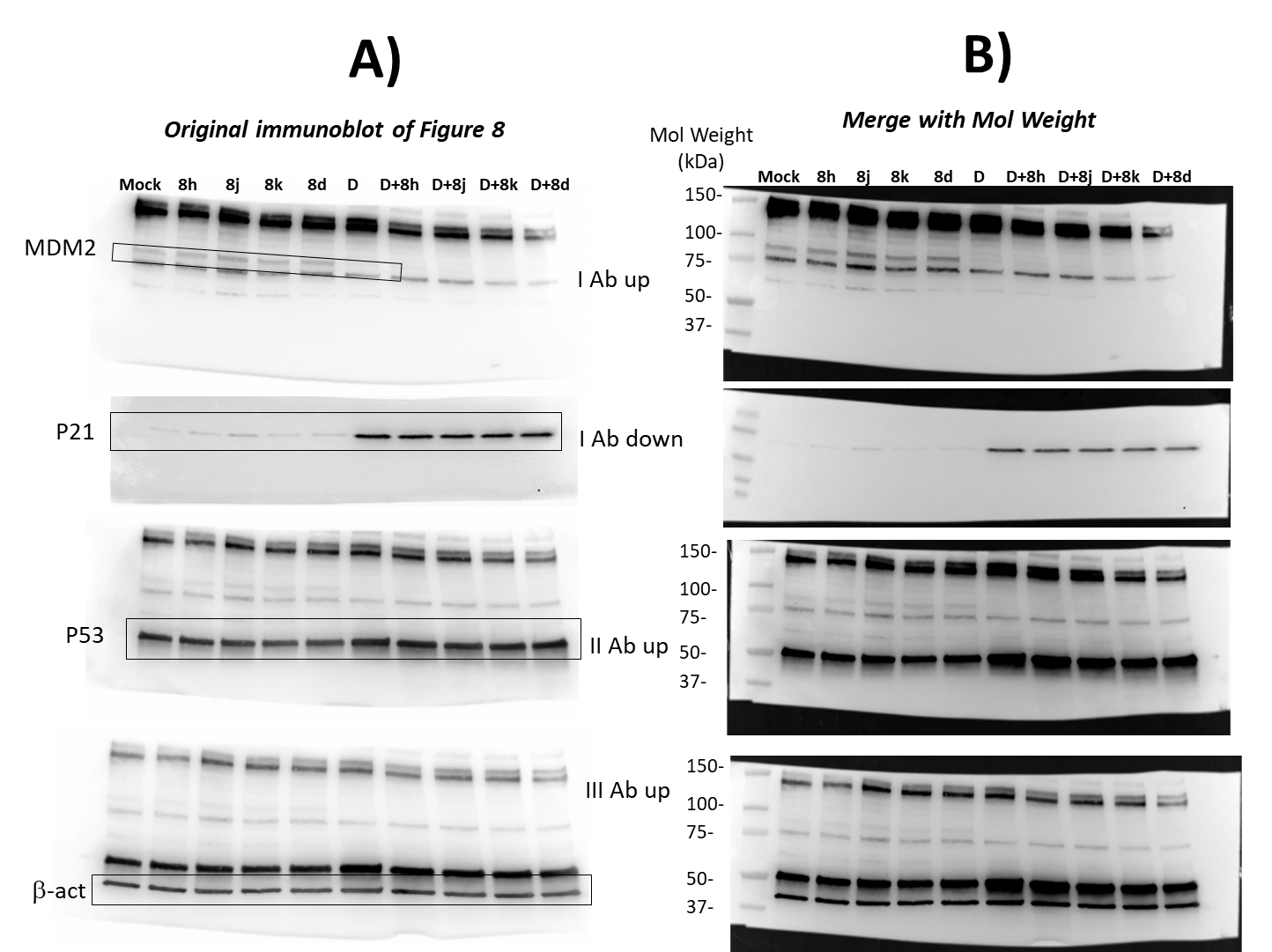


**
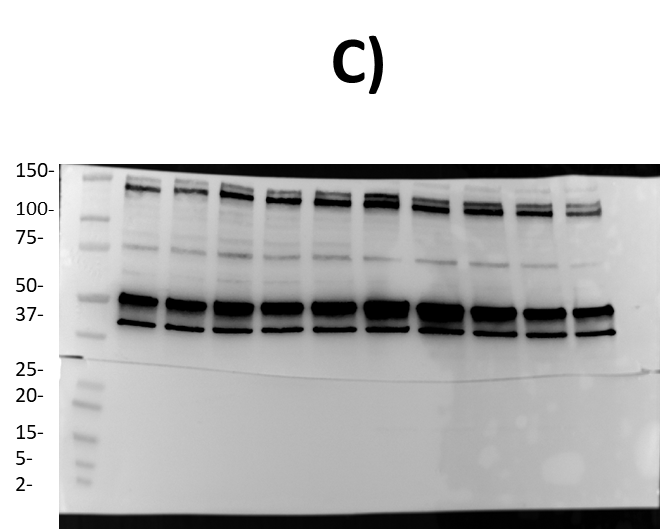
**
